# The role of the systemic inflammatory response in predicting outcomes in patients with operable cancer: Systematic review and meta-analysis

**DOI:** 10.1038/s41598-017-16955-5

**Published:** 2017-12-01

**Authors:** Ross D. Dolan, Jason Lim, Stephen T. McSorley, Paul G. Horgan, Donald C. McMillan

**Affiliations:** Academic Unit of Surgery, School of Medicine, University of Glasgow, Glasgow Royal Infirmary, Glasgow, UK

## Abstract

Cancer remains a leading causes of death worldwide and an elevated systemic inflammatory response (SIR) is associated with reduced survival in patients with operable cancer. This review aims to examine the evidence for the role of systemic inflammation based prognostic scores in patients with operable cancers. A wide-ranging literature review using targeted medical subject headings for human studies in English was carried out in the MEDLINE, EMBASE, and CDSR databases until the end of 2016. The SIR has independent prognostic value, across tumour types and geographical locations. In particular neutrophil lymphocyte ratio (NLR) (n = 158), platelet lymphocyte ratio (PLR) (n = 68), lymphocyte monocyte ratio (LMR) (n = 21) and Glasgow Prognostic Score/ modified Glasgow Prognostic Score (GPS/mGPS) (n = 60) were consistently validated. On meta-analysis there was a significant relationship between elevated NLR and overall survival (OS) (p < 0.00001)/ cancer specific survival (CSS) (p < 0.00001), between elevated LMR and OS (p < 0.00001)/CSS (p < 0.00001), and elevated PLR and OS (p < 0.00001)/CSS (p = 0.005). There was also a significant relationship between elevated GPS/mGPS and OS (p < 0.00001)/CSS (p < 0.00001). These results consolidate the prognostic value of the NLR, PLR, LMR and GPS/mGPS in patients with resectable cancers. This is particularly true for the NLR/GPS/mGPS which should form part of the routine preoperative and postoperative workup.

## Introduction

Cancer remains one of the leading causes of mortality worldwide and is responsible for 8.8 million deaths per year^1^. Overall, it has been estimated that one in three people will develop cancer in their lifetime, and one in four will die from it^2,3^. Indeed, in the UK alone it is estimated that 150,000 people die because of cancer each year^1,3^. Such a large burden of disease accounts for a significant proportion of the healthcare budgets of the UK, US and worldwide medical care^1,3,4^.

Four cancers: lung, colorectal, breast and prostate, account for approximately half of all new cases and deaths^2^. For a range of solid organ malignancies including colorectal, lung, breast and prostate cancers, definitive local therapy in the form of surgical resection remains the cornerstone of treatment^2^.

The genetic composition of many different types of cancer has been widely reported, however there is also increasing evidence that the host inflammatory response plays an important role in the development and progression of cancer^3,5–7^. In 2010 Roxburgh and McMillan published the first comprehensive review of the role of the systemic inflammatory response in predicting survival in patients with primary operable cancer^2^. They identified 80 studies where the systemic inflammatory response was related to either overall, and cancer specific survival^2^. However the majority of studies used singular markers of the inflammatory response such as CRP, albumin neutrophil, lymphocyte and platelet counts, indeed just 18 studies reported combined prognostic scores to improve prediction of survival^2^. These included eight that reported the prognostic value of the GPS, and nine studies that reported the prognostic value of NLR. While these studies reported a significant relationship between the systemic inflammatory response and survival there were variable thresholds used for the single or combined markers resulting in considerable variability in the magnitude of the effect reported^2^.

However, since this review there has been a marked increase in the number of studies reporting the prognostic value of combined scoring systems based on the systemic inflammatory response. The majority reported have principally been ratios of components of the white cell count such as the neutrophil lymphocyte ratio (NLR), platelet lymphocyte ratio (PLR), lymphocyte monocyte ratio (LMR) but also acute phase proteins such as C-reactive protein/albumin ratio (CAR). Another approach is to combine scores of the acute phase proteins such as GPS/mGPS^3,8,9^. The presence of an elevated systemic inflammatory response as shown by the presence of circulating white cells and acute phase proteins is an important unifying host characteristic in patients with cancer. The prognostic ability of the combined scores has been widely reported and there have been reviews of NLR^9,10^ and mGPS^11^ and in advanced cancer^12^. The present review is the first since 2010 to focus on primarily operable cancer and to include all recognised systemic inflammation based prognostic scores. This will rationalise the evidence for the role of systemic inflammation based prognostic scores in patients with primary operable cancers.

## Methods

This systematic review and meta-analysis of published literature was undertaken according to a pre-defined protocol described in the PRISMA-P statement and in a similar fashion to that recently reported with advanced inoperable cancer^12^. The primary outcome was to assess the prognostic value of the validated combined scores of the systemic inflammatory response (NLR, PLR, LMR, GPS and mGPS) in patients with primary operable cancer.

This was carried out by a wide-ranging literature search to identify studies carried out up to December 2016. Medical subject heading (MeSH) terms(Cancer, GPS, Glasgow Prognostic Score, mGPS, modified Glasgow Prognostic Score, NLR, Neutrophil Lymphocyte Ratio, LMR, Leucocyte Monocyte Ratio, PLR, Platelet Lymphocyte Ratio), were used in the US National Library of Medicine (MEDLINE), the Excerpta Medica database (EMBASE) and the Cochrane Database of Systematic Reviews (CDSR) to identify articles.

On completion of the online search, the title and abstract of each identified study was examined for relevance. Studies not in cancer patients, studies not available in English and those published in abstract form only were excluded. Where there were multiple publications from the same cohort the most recent paper was included. Full texts were obtained for all studies deemed potentially relevant. Once further exclusions outlined below were carried out, the bibliographies of all included articles were subsequently hand searched to identify any additional studies.

Only articles that reported survival analysis and gave hazard ratios (HR) with associated confidence intervals were included in the final meta-analysis. Articles reporting survival analysis in relative risk (RR) and odds ratio (OR) were also included but not in the meta-analysis. Studies that did not follow the majority of other studies in terms of score or ratio direction interpretation were excluded from the final meta-analysis. Studies with patients who had chemotherapy and/or radiotherapy before or after surgery were also included.

### Statistics

The HRs and 95% CIs were directly retrieved from the article. If several estimates were reported for the same marker, the multivariate estimate was used in preference to the univariate analysis. Data was assessed for heterogeneity using the I^2^ statistic and χ^2^ test interpreted using the guidance from the Cochrane Handbook for Systematic Reviews of Interventions^13^. The degrees of heterogeneity were defined as minimal between 0% and 30%, moderate between 30% and 50%, substantial between 50% and 80% and considerable between 80% and 100%. Given the likely differences in methodology of the studies included, meta-analysis was performed using the random- effects (DerSimonian – Laird method) model. The Z test was used to assess the overall impact of systemic inflammation based scores on overall and cancer specific survival. All P values were 2-sided and P < 0.05 were considered statistical significant. Evidence of publication bias was evaluated using visual inspection of funnel plots. All analyses were performed using Review Manager (RevMan) [Computer program]. Version 5.3. Copenhagen: The Nordic Cochrane Centre, the Cochrane Collaboration, 2014.

## Results

### Study selection process

**Figure 1:**
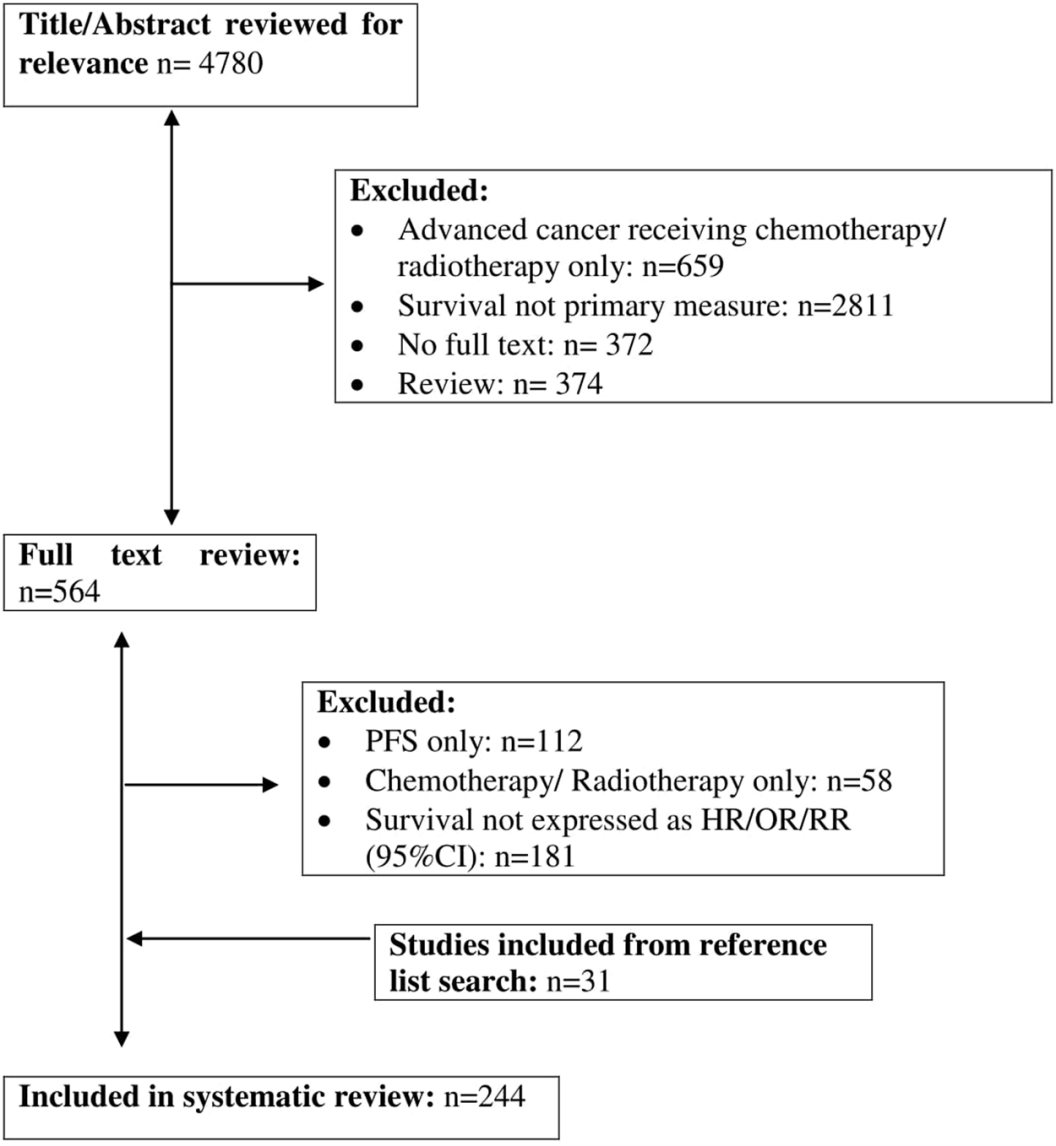
PRISMA flowchart demonstrating study selection.

The study selection process is summarised in Fig. 1. Initial search strategy identified 4780 articles whose titles and abstracts were reviewed. Articles were excluded if the treatment regime was chemotherapy/radiotherapy only (n = 659), where survival was not the primary outcome measure (n = 2811), full articles were not available (n = 372), and those that were a systematic review/meta-analysis (n = 374).

This led to a review of the full text of 564 articles. A further 351 articles were excluded if progression free survival (PFS) was the only outcome measured (n = 112), if the treatment regime was chemotherapy/radiotherapy only (n = 58) and if survival was not expressed as HR/OR/RR (95% CI; n = 181). The remaining 213 articles, had their bibliographies reviewed in a systematic manner and this identified a further 31 articles to be included in the final analysis leading to final figure of 244 articles considered in the present systematic review and meta-analysis.

### Studies of the prognostic value of Glasgow Prognostic Score (GPS) or modified Glasgow Prognostic Score (mGPS) in patients with primary operable cancer

Eighty articles with both overall survival (OS) and/or cancer specific survival (CSS) as their primary outcome measures were identified (Supplementary Table). This comprised data on 25,207 patients (9,361 deaths) reporting the significant prognostic value of GPS/mGPS in cohorts of patients with primary operable cancer (Supplementary Table). Seventy two studies were carried out in a retrospective manner while eight were prospective (Supplementary Table). Seventy two studies used multivariate and eight used univariate survival analysis (Supplementary Table).

**Figure 2:**
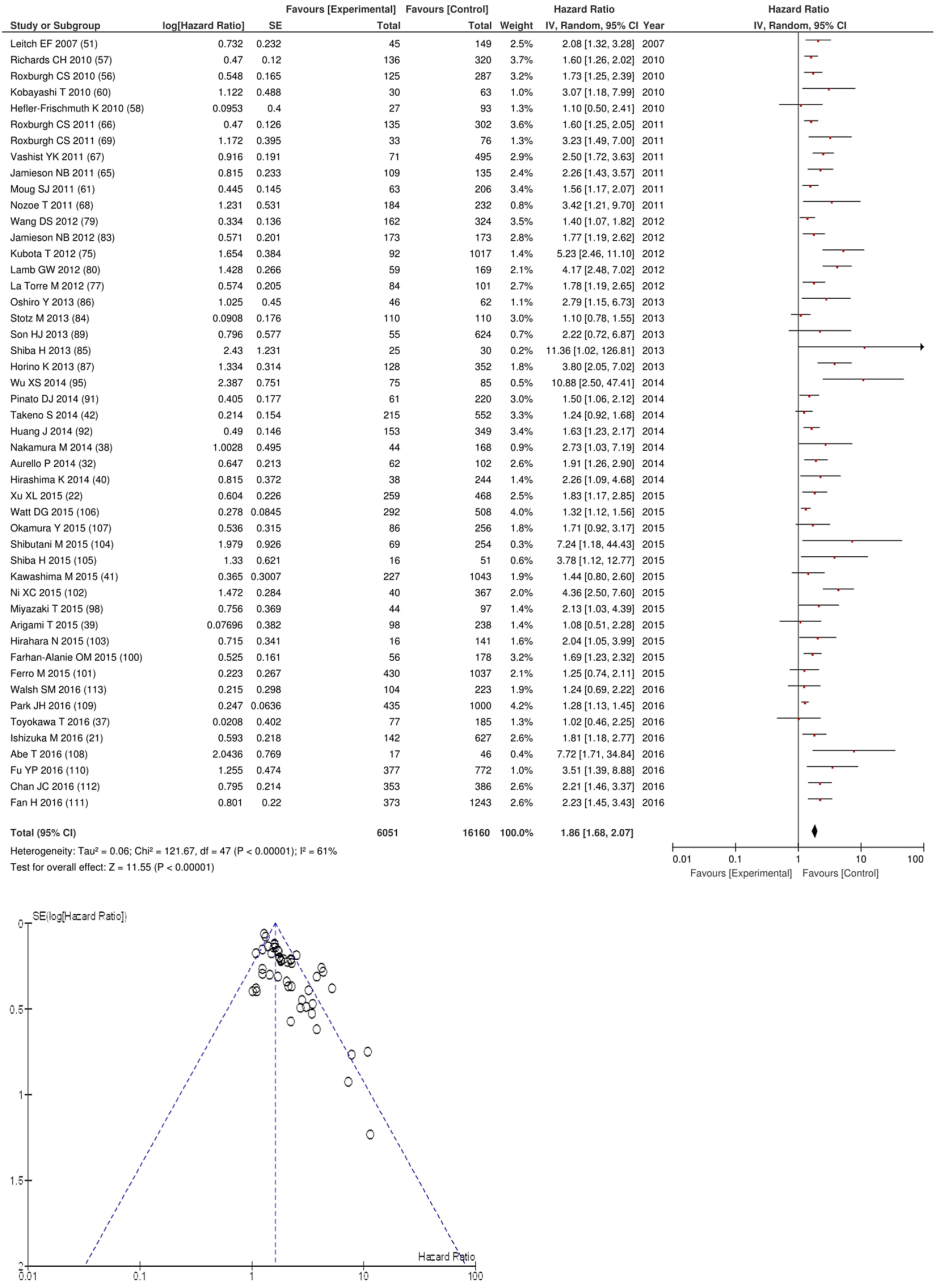
Forrest and Funnel Plot of Studies investigating the prognostic value of GPS/mGPS in terms of OS in an unselected cohort of patients with operable cancer.

After exclusion forty eight studies examined the relationship with overall survival including 16,160 patients (6,051 deaths), as the primary outcome measure. On meta-analysis there was a significant association between GPS/mGPS and overall survival (HR 1.86 95% CI 1.68–2.07, p < 0.00001) with a substantial degree of heterogeneity (I^2^ = 61%, Fig. 2). These included studies on colorectal (n = 12), oesophageal (n = 7), liver (n = 6), gastric (n = 6), pancreatic (n = 5), lung (n = 4), gallbladder (n = 2), colorectal liver metastases (n = 1), renal (n = 1), bladder (n = 1), cholangiocarcinoma (n = 1), oral (n = 1) and vulval cancers (n = 1).

**Figure 3:**
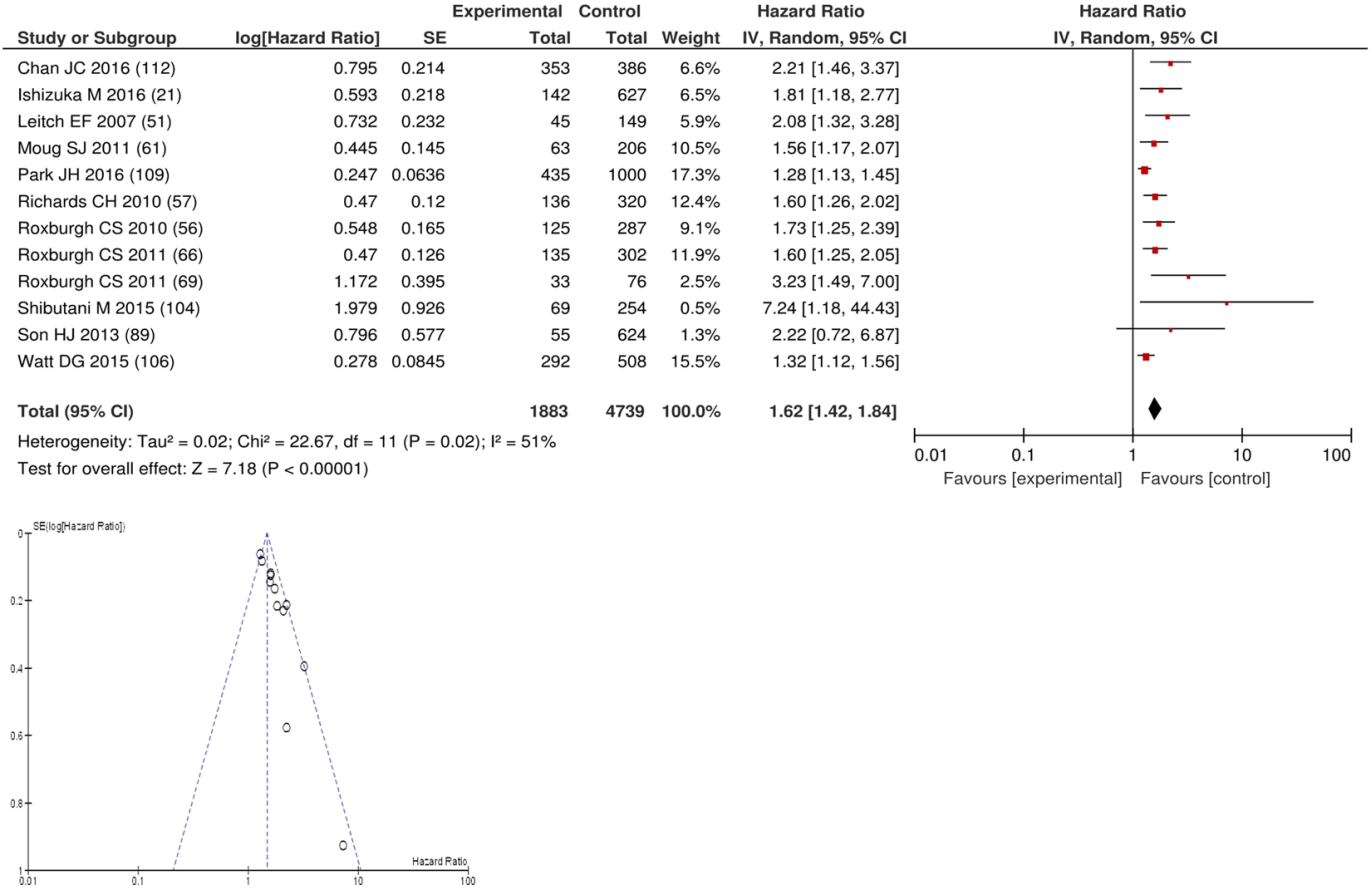
Forrest and Funnel Plot of Studies investigating the prognostic value of GPS/mGPS in terms of OS in patients with operable colorectal cancer.

On meta-analysis of those studies carried out in colorectal cancer (n = 12), including 4,739 patients (1,883 deaths), there was a significant association between elevated GPS/mGPS and overall survival (HR: 1.62 95% CI 1.42–1.84, p < 0.00001) with a substantial degree of heterogeneity (I^2^ = 51%, Fig. 3). These included studies carried out in the UK (n = 8), Japan (n = 2), Korea (n = 1) and Australia (n = 1). The proportion of patients who had an elevated GPS/mGPS was 60% in Australia, 39% in Japan, 37% in the UK and 21% in Korea.

**Figure 4:**
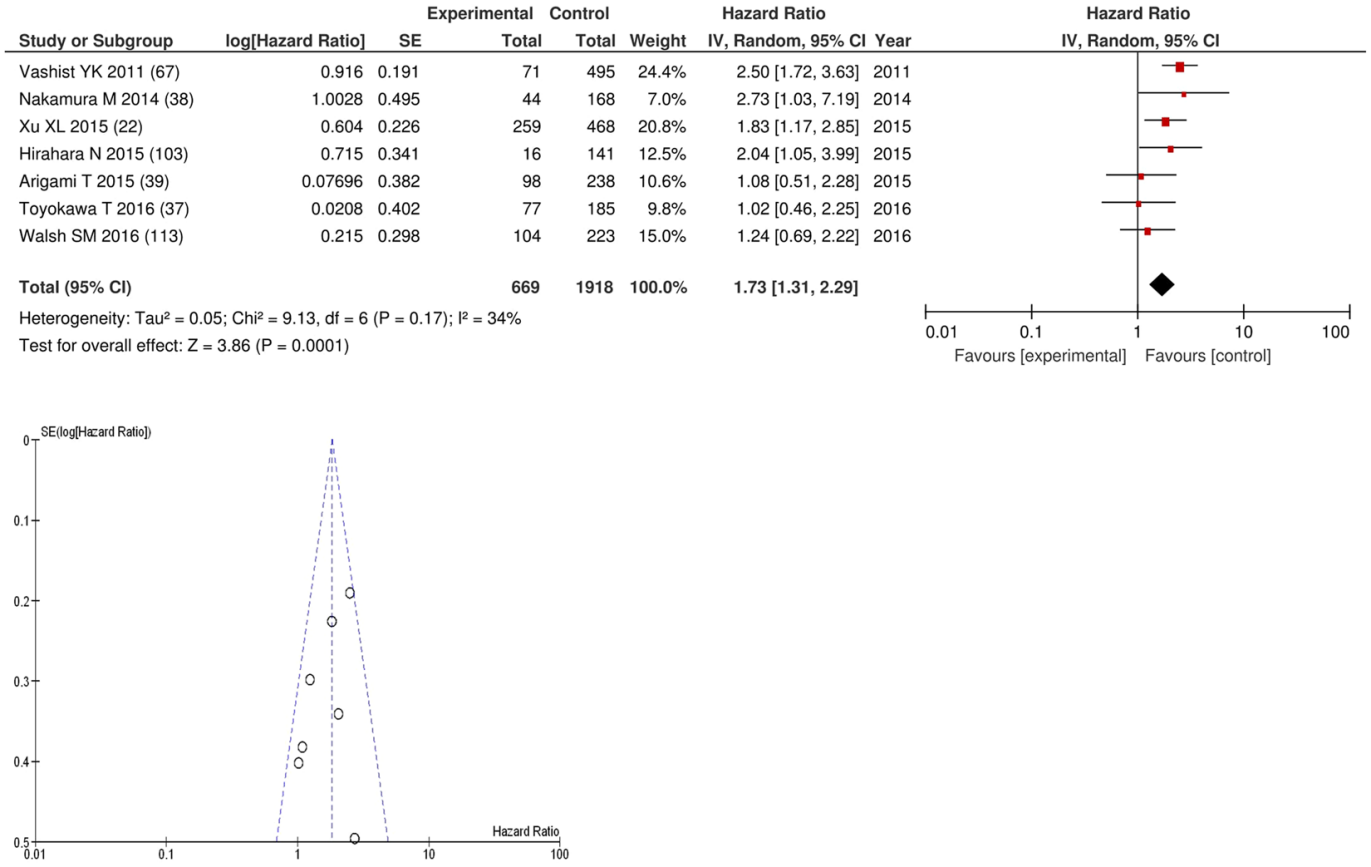
Forrest and Funnel Plot of Studies investigating the prognostic value of GPS/mGPS in terms of OS in patients with operable oesophageal cancer.

On meta-analysis of studies involving oesophageal cancer (n = 7), including 1,918 patients (669 deaths), there was a significant association between GPS/mGPS and overall survival (HR: 1.73 95% CI 1.31–2.29, p < 0.0001) with a minimal degree of heterogeneity (I^2^ = 34%, Fig. 4). These included studies carried out in Japan (n = 4), Germany (n = 1), China (n = 1) and Ireland (n = 1). The proportion of patients who had an elevated GPS/mGPS was 19% in Japan, 46% in Germany, 28% in China and 22% in Ireland.

**Figure 5:**
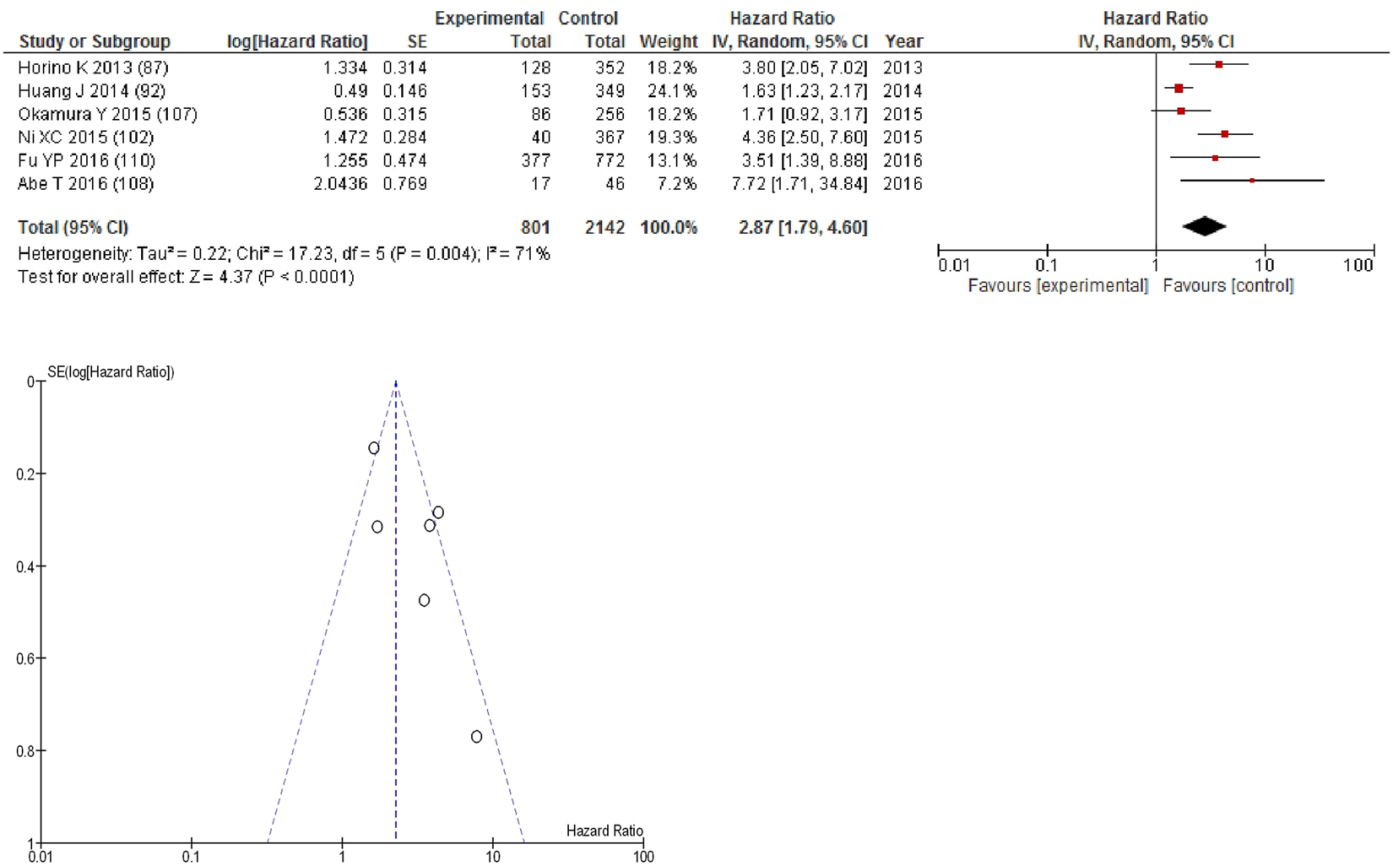
Forrest and Funnel Plot of Studies investigating the prognostic value of GPS/mGPS in terms of OS in patients with operable liver cancer.

On meta-analysis of studies involving liver cancer (n = 6), including 2,142 patients (801 deaths), there was a significant association between GPS/mGPS and overall survival (HR: 2.87 95% CI 1.79–4.60, p < 0.0001) with a substantial degree of heterogeneity (I^2^ = 71%, Fig. 5). These included studies carried out in Japan (n = 3) and China (n = 3). The proportion of patients who had an elevated GPS/mGPS was 20% in Japan and 12% in China.

**Figure 6:**
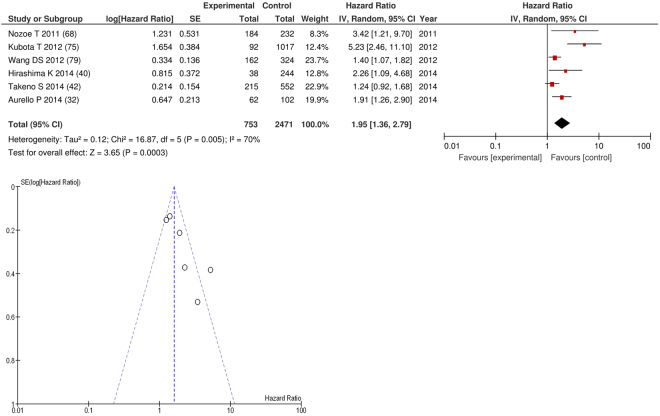
Forrest and Funnel Plot of Studies investigating the prognostic value of GPS/mGPS in terms of OS in patients with operable gastric cancer.

On meta-analysis of studies involving gastric cancer (n = 6), including 2,471 patients (753 deaths), there was a significant association between GPS/mGPS and overall survival (HR: 1.95 95% CI 1.36–2.79, p = 0.0003) with a substantial degree of heterogeneity (I^2^ = 70%, Fig. 6). These included studies carried out in Japan (n = 4), China (n = 1) and Italy (n = 1). The proportion of patients who had an elevated GPS/mGPS was, 30% in Japan, 23% in China and 52% in Italy.

**Figure 7:**
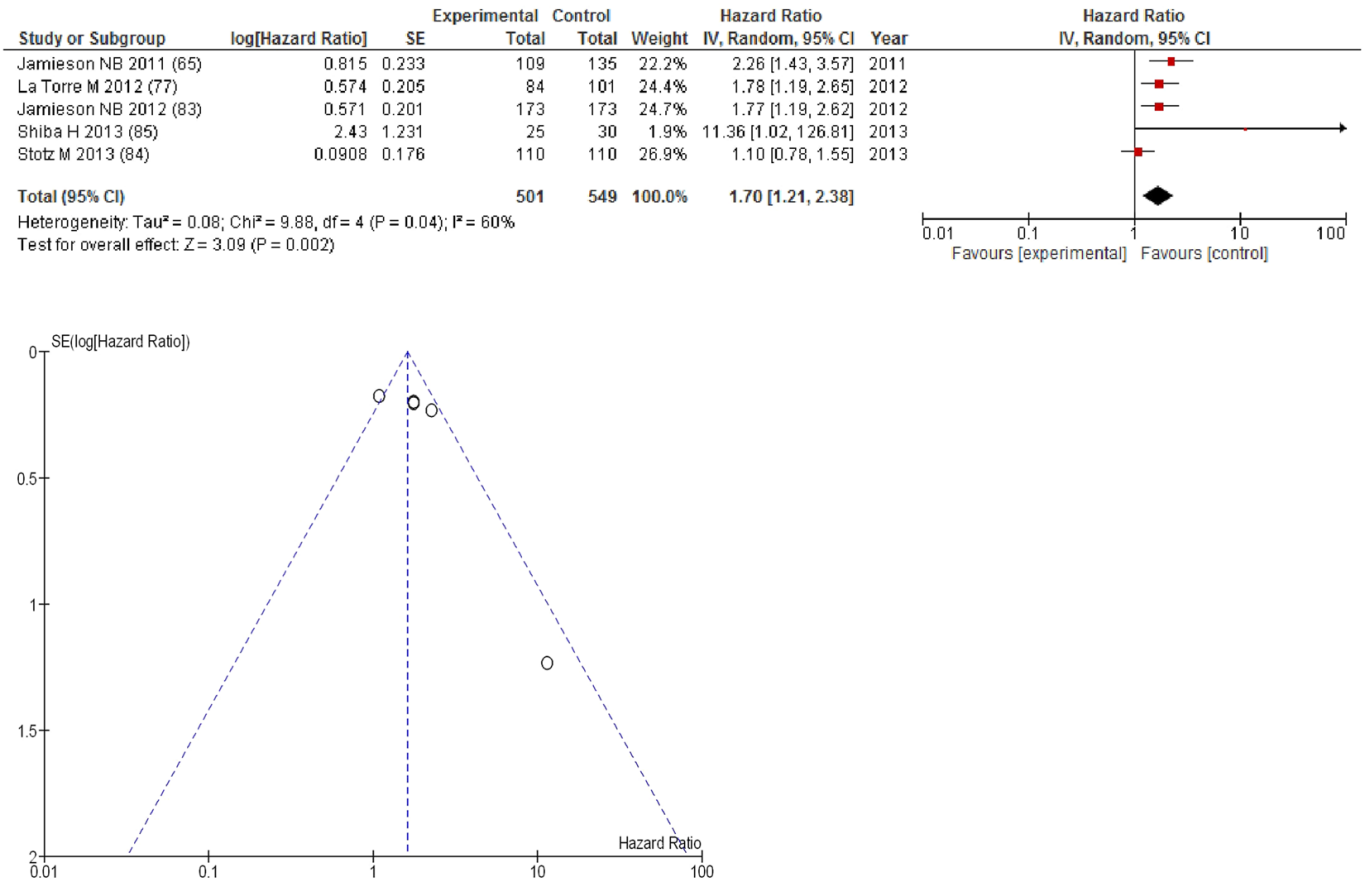
Forrest and Funnel Plot of Studies investigating the prognostic value of GPS/mGPS in terms of OS in patients with operable pancreatic cancer.

On meta-analysis those studies carried out in pancreatic cancer (n = 5), including 549 patients (501 deaths), there was a significant association between GPS/mGPS and overall survival (HR: 1.70 95% CI 1.21–2.38, p = 0.002) with a substantial degree of heterogeneity (I^2^ = 60%, Fig. 7). These included studies carried out in the UK (n = 2), Japan (n = 1), Italy (n = 1) and Austria (n = 1). The proportion of patients who had an elevated GPS/mGPS was 45% in the UK, 23% in Japan, 68% in Italy and 34% in Austria.

**Figure 8:**
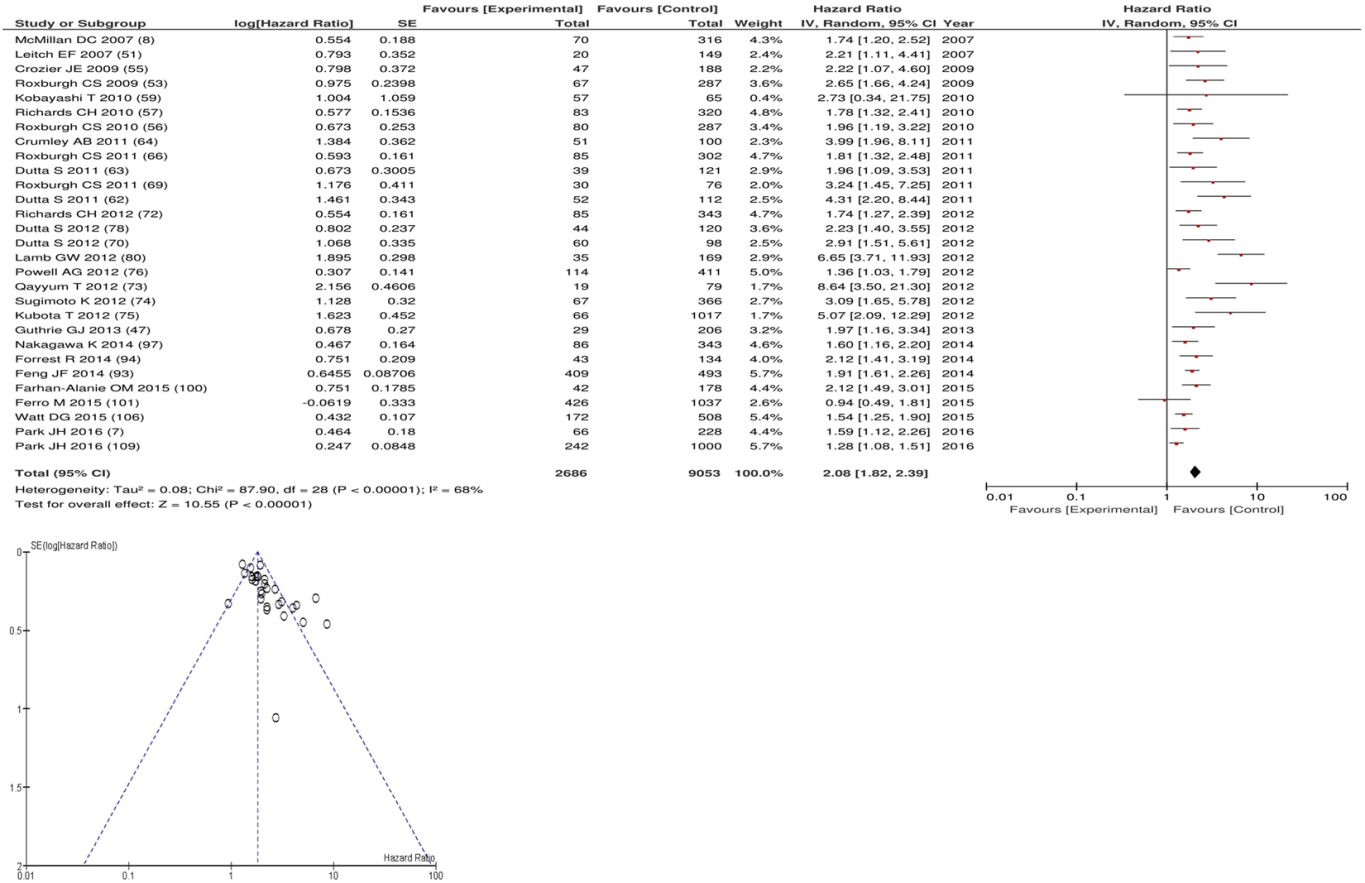
Forrest and Funnel Plot of Studies investigating the prognostic value of GPS/mGPS in terms of CSS in an unselected cohort of patients with operable cancer.

After exclusion twenty nine studies examined cancer specific survival (CSS) including 9,053 patients (2,686 deaths), as its primary outcome measure. On meta-analysis there was a significant association between GPS/mGPS and cancer specific survival (HR 2.08 95% CI 1.82–2.39, p < 0.00001) with a substantial degree of heterogeneity (I^2^ = 68%, Fig. 8). These included studies on colorectal (n = 16), oesophageal (n = 4), oesophago-gastric (n = 2), gastric (n = 2), renal cell (n = 2), colorectal liver metastases (n = 1), oral (n = 1) and bladder cancers (n = 1).

**Figure 9:**
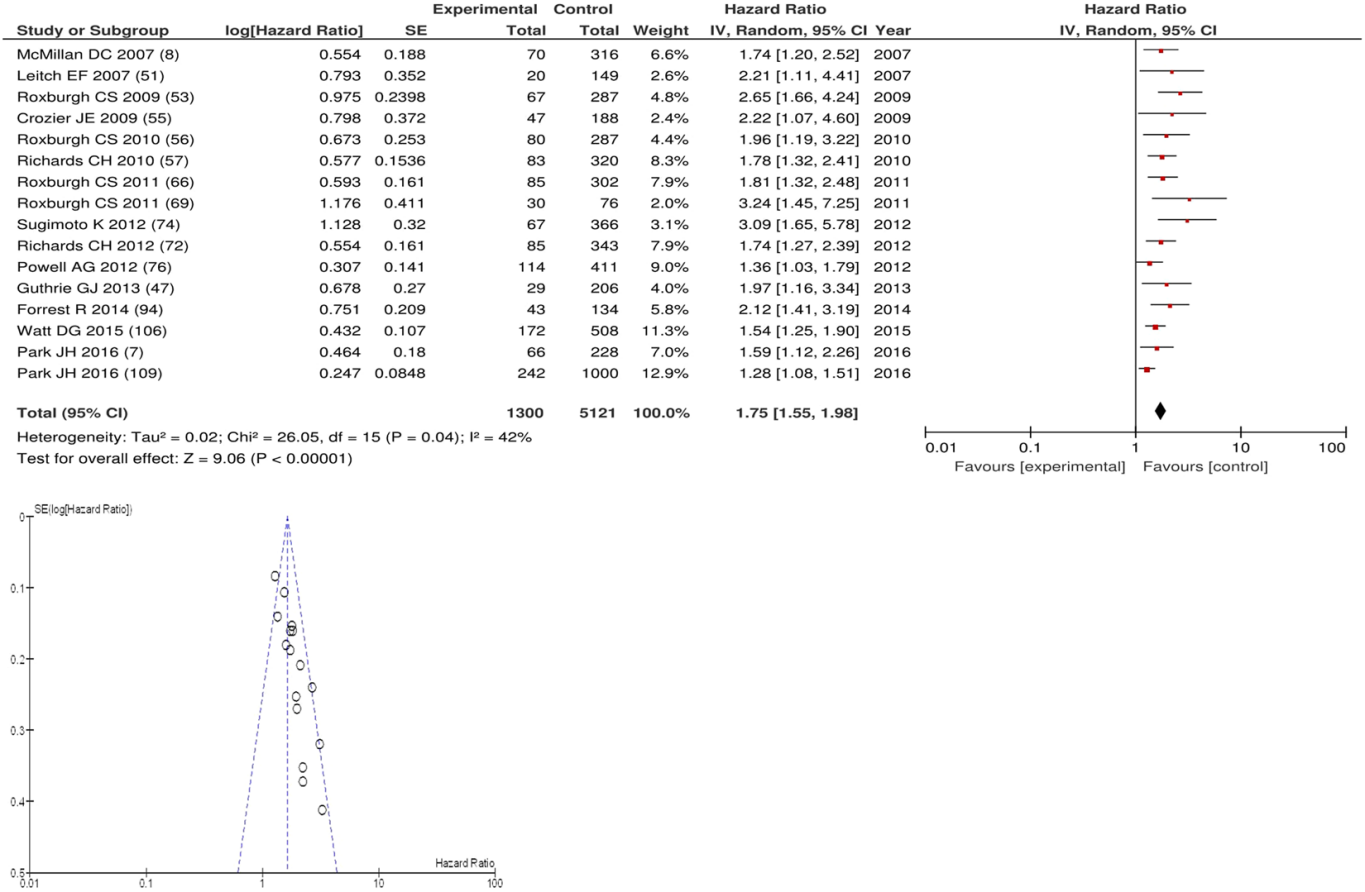
Forrest and Funnel Plot of Studies investigating the prognostic value of GPS/mGPS in terms of CSS in patients with operable colorectal cancer.

On meta-analysis of studies involving colorectal cancer (n = 16), including 5121 patients (1300 deaths), there was a significant association between GPS/mGPS and cancer specific survival (HR: 1.75 95% CI 1.55–1.98, p < 0.00001) with a moderate degree of heterogeneity (I^2^ = 42%, Fig. 9). These included studies carried out in the UK (n = 15) and Japan (n = 1). The proportion of patients who had an elevated GPS/mGPS was 39% in the UK and 8% in Japan.

### Studies of the prognostic value of Neutrophil Lymphocyte Ratio (NLR) in patients with primary operable cancer

One hundred and fifty eight articles with both overall survival (OS) and/or cancer specific survival (CSS) as their primary outcome measures were identified (Supplementary Table). This comprised data on 63,837 patients (22,681 deaths) reporting the significant prognostic value of NLR in cohorts of patients with primary operable cancer. All one hundred and fifty eight studies were carried out in a retrospective manner (Supplementary Table). One hundred and twenty eight studies used multivariate and thirty used univariate survival analysis (Supplementary Table).

**Figure 10:**
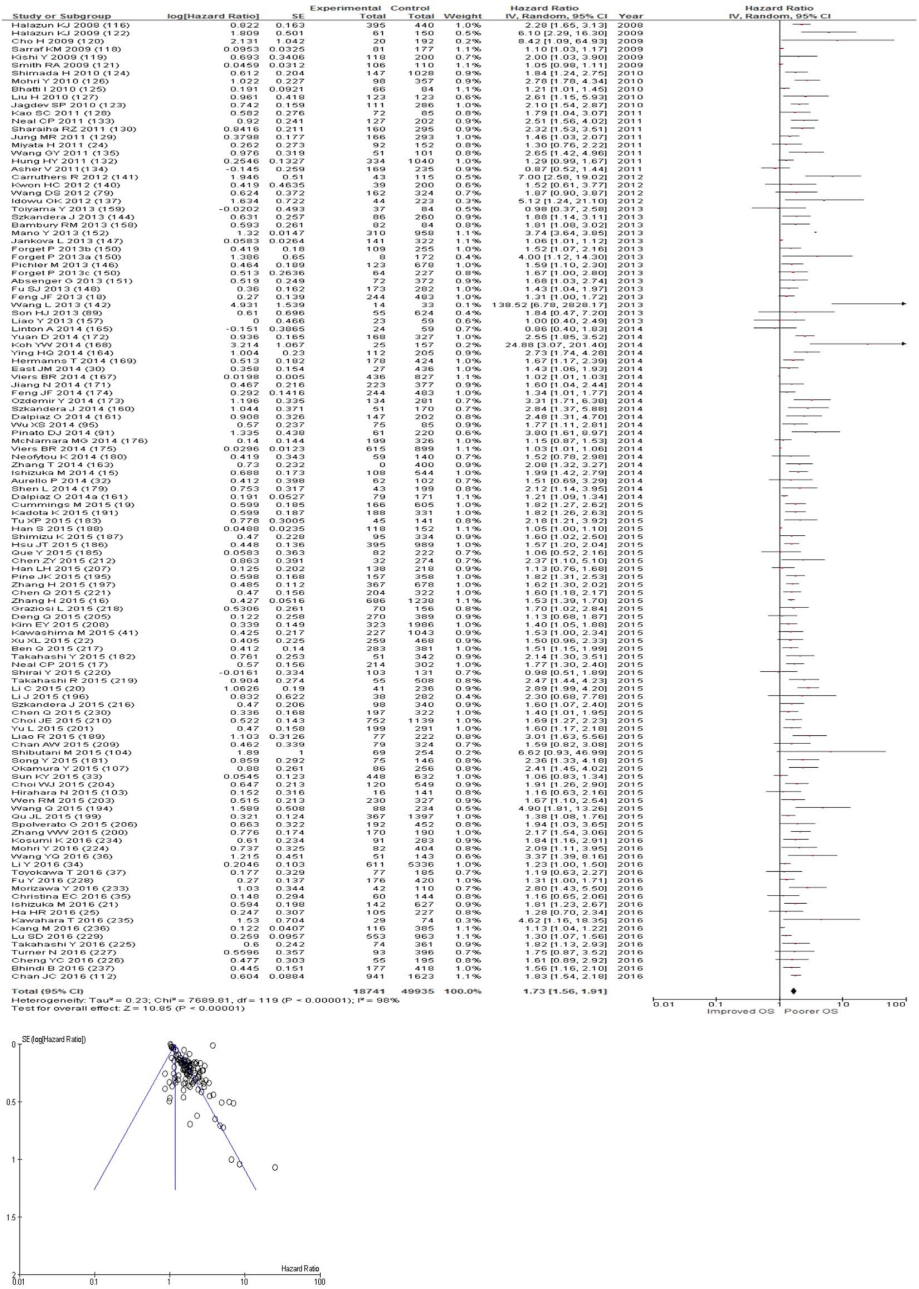
Forrest and Funnel Plot of Studies investigating the prognostic value of NLR in terms of OS in an unselected cohort of patients with operable cancer.

After exclusion one hundred and nineteen studies examined the relationship with overall survival including 49,664 patients (18,542 deaths), as the primary outcome measure. On meta-analysis there was a significant association between NLR and overall survival (HR 1.73 95% CI 1.56–1.91, p < 0.00001) with a considerable degree of heterogeneity (I^2^ = 98%, Fig. 10). The most common NLR threshold examined was ≥5 (n = 29). Other thresholds were ≥3 (n = 9), ≥2.5 (n = 7), NLR as continuous variable (n = 7), ≥4 (n = 7) and ≥2 (n = 5). Other thresholds were used in <5 studies and thus, meta-analysis was not carried out (n = 55).

**Figure 11:**
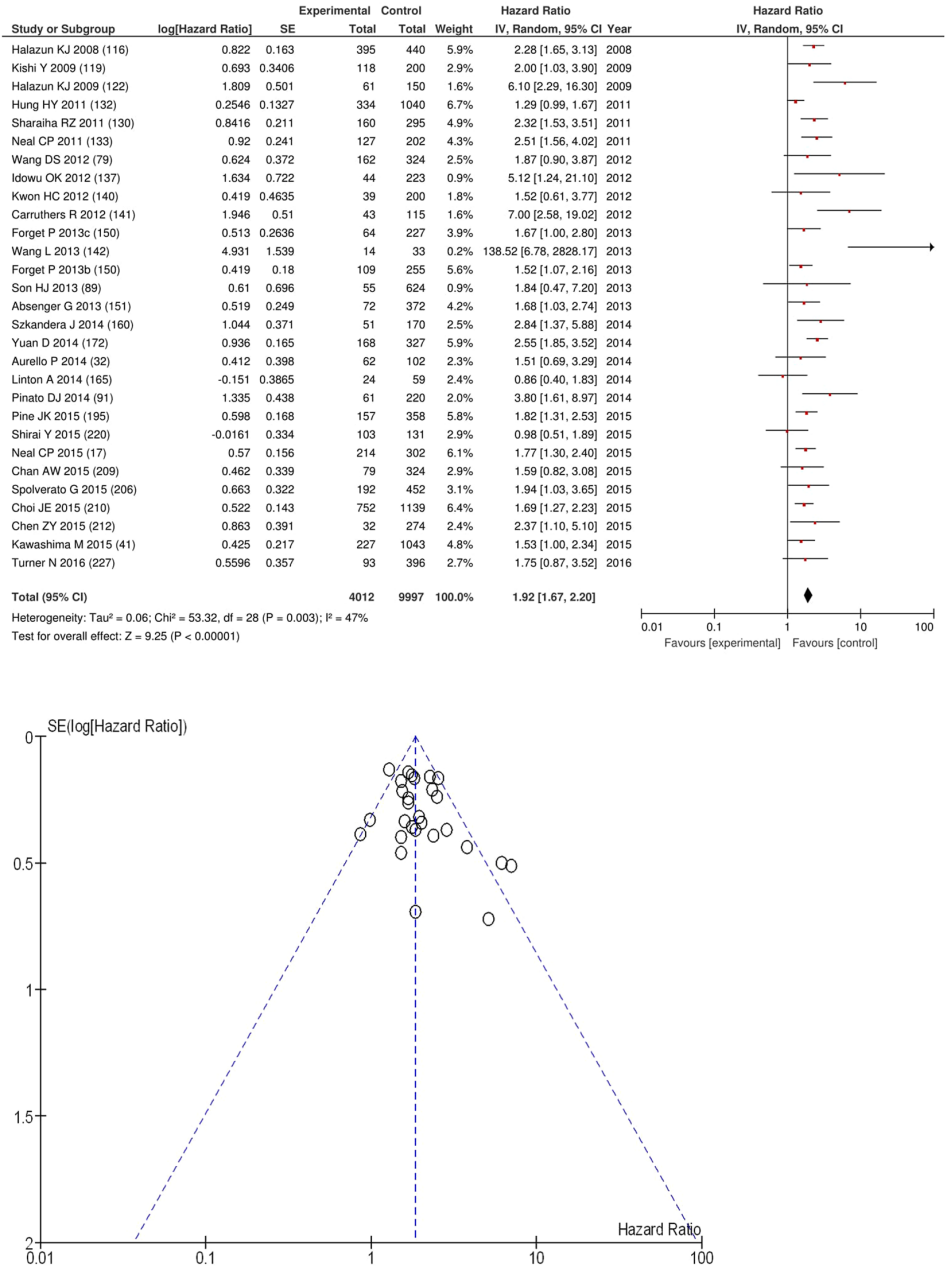
Forrest and Funnel Plot of Studies investigating the prognostic value of NLR ≥5 in terms of OS in an unselected cohort of patients with operable cancer.

On meta-analysis of those studies with a threshold of ≥5 (n = 29), including 9,997 patients (4,012 deaths) there was a significant association between elevated NLR and overall survival (HR: 1.92 95% CI 1.67–2.20, p < 0.00001) with a moderate degree of heterogeneity (I^2^ = 47%, Fig. 11). These included colorectal (n = 8), lung (n = 4), colorectal liver metastases (n = 4), oesophageal (n = 3), gastric (n = 2), soft tissue sarcoma (n = 2), liver (n = 2), pancreatic (n = 1), renal (n = 1), pleural mesothelioma (n = 1) and hepato-pancreatico-biliary cancers (n = 1).

**Figure 12:**
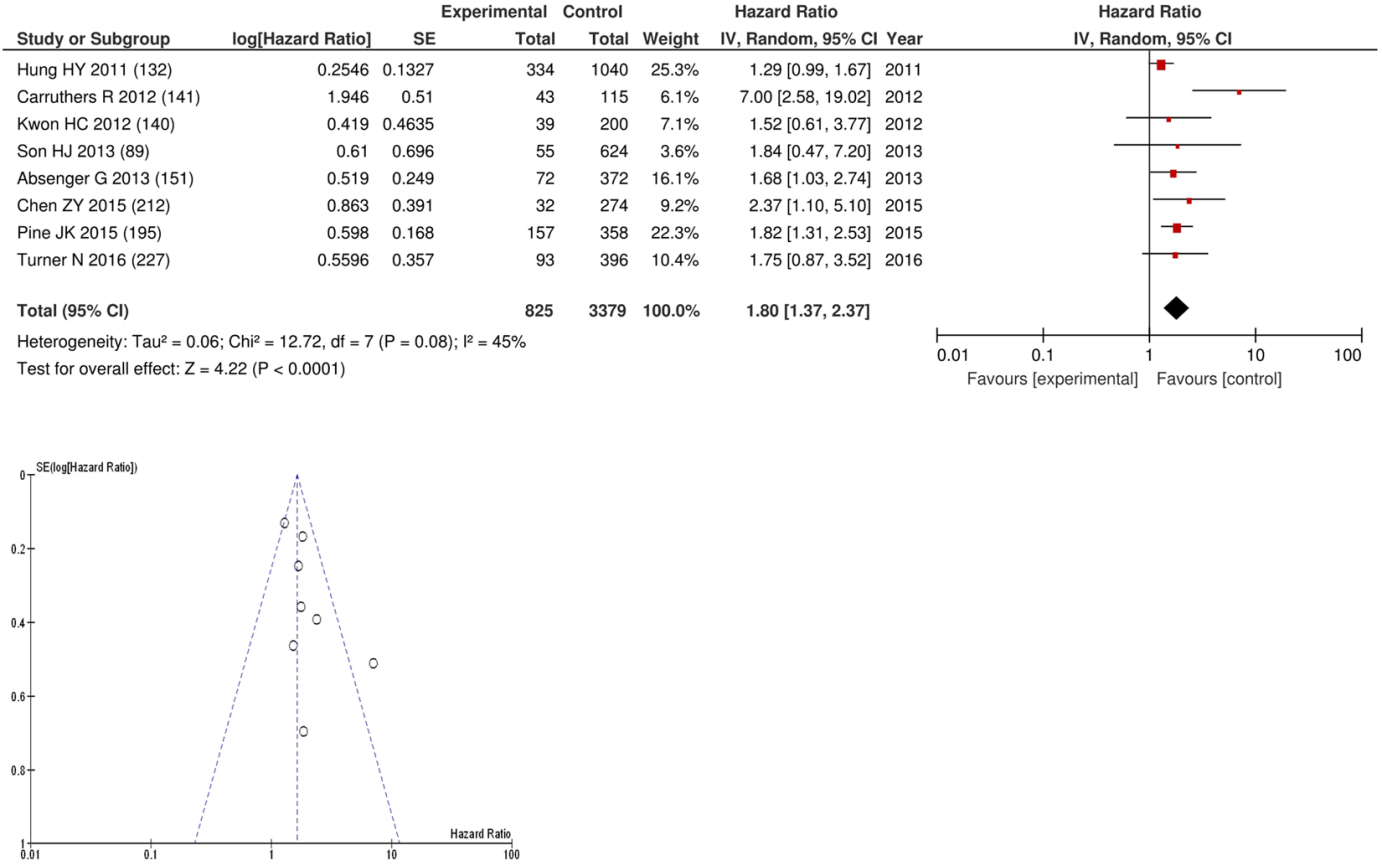
Forrest and Funnel Plot of Studies investigating the prognostic value of NLR ≥5 in terms of OS in patients with operable colorectal cancer.

On meta-analysis of those studies with a threshold of ≥5 and colorectal cancer (n = 8), including 3,379 patients (825 deaths)there was a significant association between an NLR ≥5 and overall survival (HR: 1.80 95% CI 1.37–2.37, p < 0.0001) with moderate heterogeneity (I^2^ = 45%, Fig. 12). In these eight studies, there was a variation in their geographical locations including the UK (n = 2), Korea (n = 2), Taiwan (n = 1), Austria (n = 1), US (n = 1) and Australia (n = 1). The proportion of patients who had an NLR ≥5 with colorectal cancer was 25% in the UK, 5% in Korea, 25% in Taiwan, 11% in US and 30% in Australia. 29% in Korea and 20% in Japan. No country had more than 4 studies and therefore no further meta-analysis was carried out.

**Figure 13:**
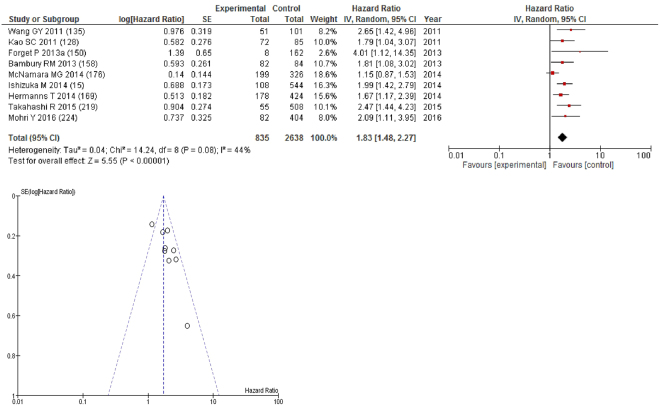
Forrest and Funnel Plot of Studies investigating the prognostic value of NLR ≥3 in terms of OS in an unselected cohort of patients with operable cancer.

On meta-analysis of those studies with a threshold of ≥3 (n = 9), including 2,638 patients (835 deaths) there was a significant association between elevated NLR and overall survival (HR: 1.83 95% CI 1.48–2.27, p < 0.00001) with a moderate degree of heterogeneity (I^2^ = 44%, Fig. 13). These included gastric (n = 2), liver (n = 1), biliary tract (n = 1), bladder (n = 1), breast (n = 1), colorectal (n = 1), pleural mesothelioma (n = 1) and endometrial cancers (n = 1). In these nine studies, there was a variation in their geographical locations including Japan (n = 4), Canada (n = 2), China (n = 1), Belgium (n = 1) and Australia (n = 1). The proportion of patients who had an NLR ≥3 was 28% in Japan, 47% in Canada, 33% in China, 31% in Belgium and 52% in Australia. No tumour site had more than four studies and therefore no further meta-analysis was carried out.

**Figure 14:**
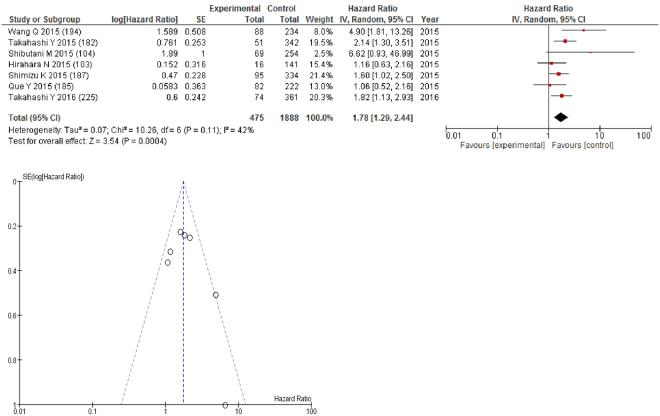
Forrest and Funnel Plot of Studies investigating the prognostic value of NLR ≥2.5 in terms of OS in an unselected cohort of patients with operable cancer.

On meta-analysis of those studies with a threshold of ≥2.5 (n = 7), including 1,888 patients (475 deaths) there was a significant association between elevated NLR and overall survival (HR: 1.78 95% CI 1.29–2.44, p = 0.0004) with a moderate degree of heterogeneity (I^2^ = 42%, Fig. 14). These included lung (n = 3), oesophageal (n = 1), colorectal (n = 1), soft tissue sarcoma (n = 1) and liver cancers (n = 1). In these seven studies, there was a variation in their geographical locations including Japan (n = 5), China (n = 1) and US (n = 1). The proportion of patients who had an NLR ≥2.5 was 30% in Japan, 28% in China and 50% in US. No tumour site had more than four studies and therefore no further meta-analysis was carried out.

**Figure 15:**
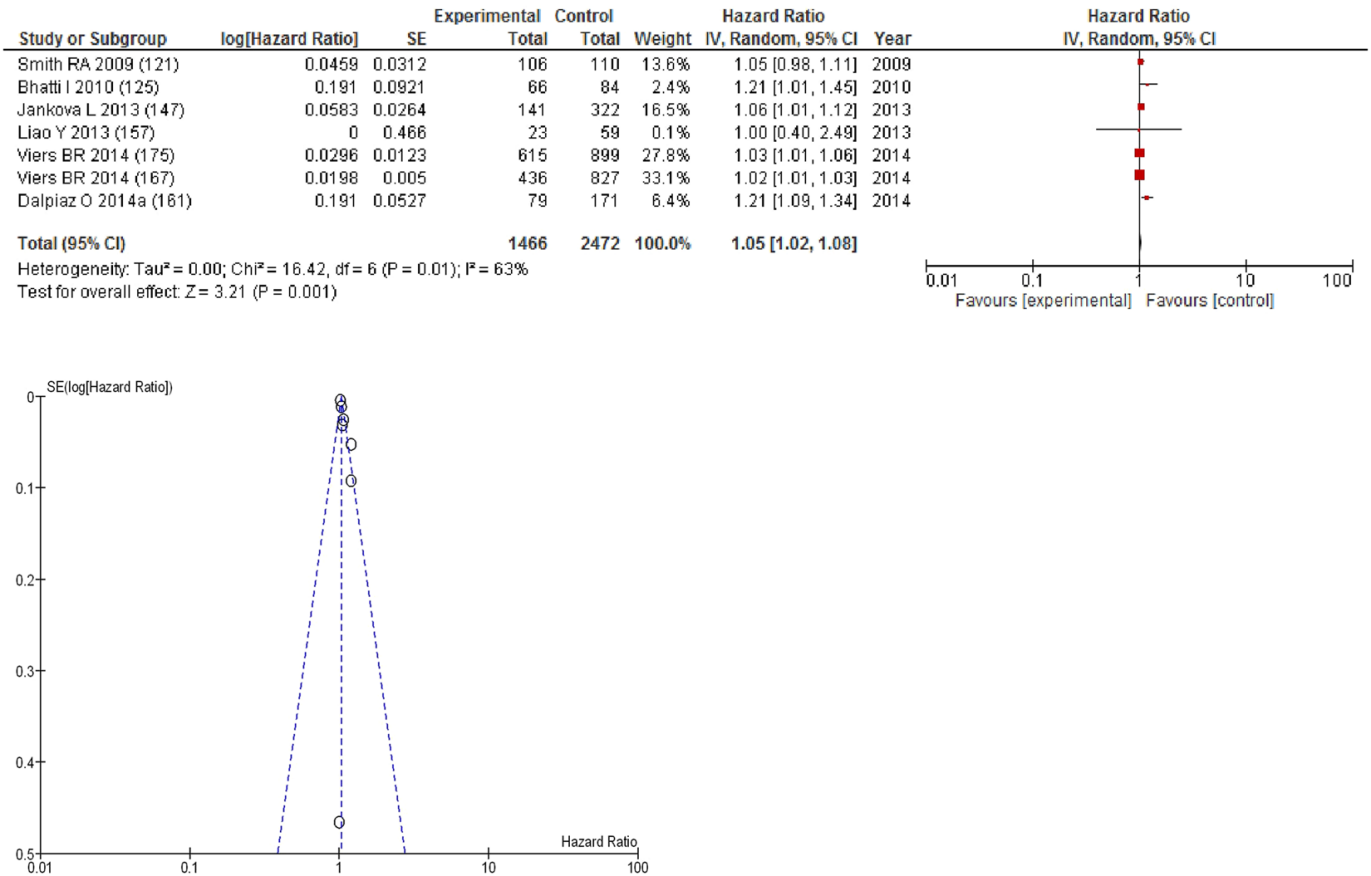
Forrest and Funnel Plot of Studies investigating the prognostic value of NLR as a continuous variable in terms of OS in an unselected cohort of patients with operable cancer.

On meta-analysis those studies with NLR as continuous variable (n = 7), including 2,472 patients (1,466 deaths) there was a moderate association between elevated NLR and overall survival (HR: 1.05 95% CI 1.02–1.08, p = 0.001) with a substantial degree of heterogeneity (I^2^ = 63%, Fig. 15). These included pancreatic (n = 2), renal (n = 2), colorectal (n = 1), lung (n = 1) and bladder cancers (n = 1). In these seven studies, there was a variation in their geographical locations including the UK (n = 2), US (n = 2), China (n = 1), Austria (n = 1) and Australia (n = 1). No tumour site had more than four studies and therefore no further meta-analysis was carried out.

**Figure 16:**
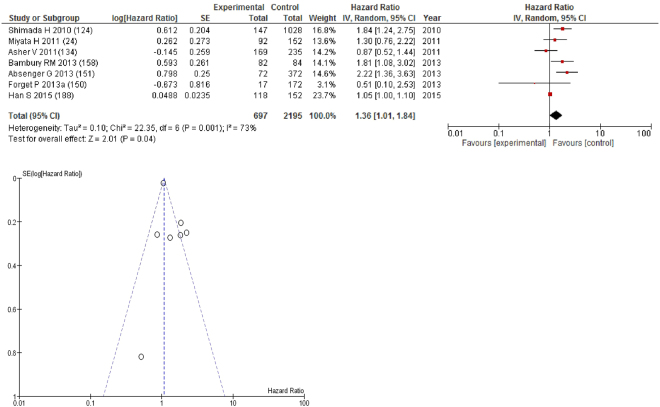
Forrest and Funnel Plot of Studies investigating the prognostic value of NLR ≥4 in terms of OS in an unselected cohort of patients with operable cancer.

On meta-analysis those studies with a threshold of ≥4 (n = 7), including 2,195 patients (697 deaths) there was a significant association between elevated NLR and overall survival (HR: 1.36 95% CI 1.01–1.84, p = 0.04) with a substantial degree of heterogeneity (I^2^ = 73%, Fig. 16). These included glioblastoma (n = 2), gastric (n = 1), oesophageal (n = 1), ovarian (n = 1), breast (n = 1) and colon cancers (n = 1). In these seven studies, there was a variation in their geographical locations including Japan (n = 2), China (n = 1), the UK (n = 1), Belgium (n = 1), Austria (n = 1) and Ireland (n = 1). The proportion of patients who had an NLR ≥4 was 15% in Japan, 32% in China, 22% in Belgium and 36% in Ireland. No tumour site had more than four studies and therefore no further meta-analysis was carried out.

**Figure 17:**
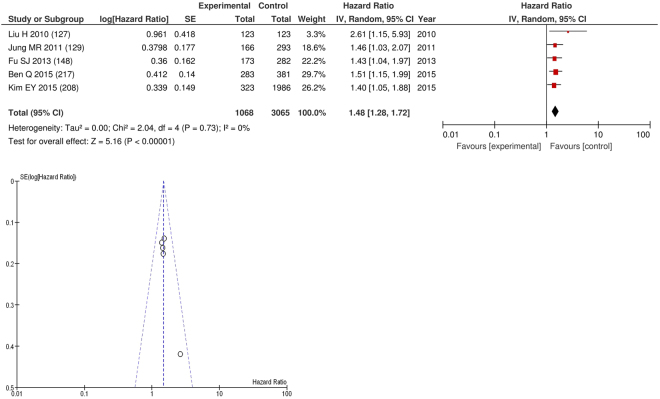
Forrest and Funnel Plot of Studies investigating the prognostic value of NLR ≥2 in terms of OS in an unselected cohort of patients with operable cancer.

On meta-analysis those studies with a threshold of ≥2 (n = 5), including 3,065 patients (1,068 deaths) there was a significant association between elevated NLR and overall survival (HR: 1.48 95% CI 1.28–1.72, p < 0.00001) with minimal heterogeneity (I^2^ = 0%, Fig. 17). These cancers included gastric (n = 2), colorectal (n = 1), liver (n = 1) and pancreatic (n = 1). In these five studies, there was a variation in their geographical locations including China (n = 3) and Korea (n = 2). The proportion of patients who had an NLR ≥2 was 60% in China and 39% in Korea. No tumour site had more than four studies and therefore no further meta-analysis was carried out.

**Figure 18:**
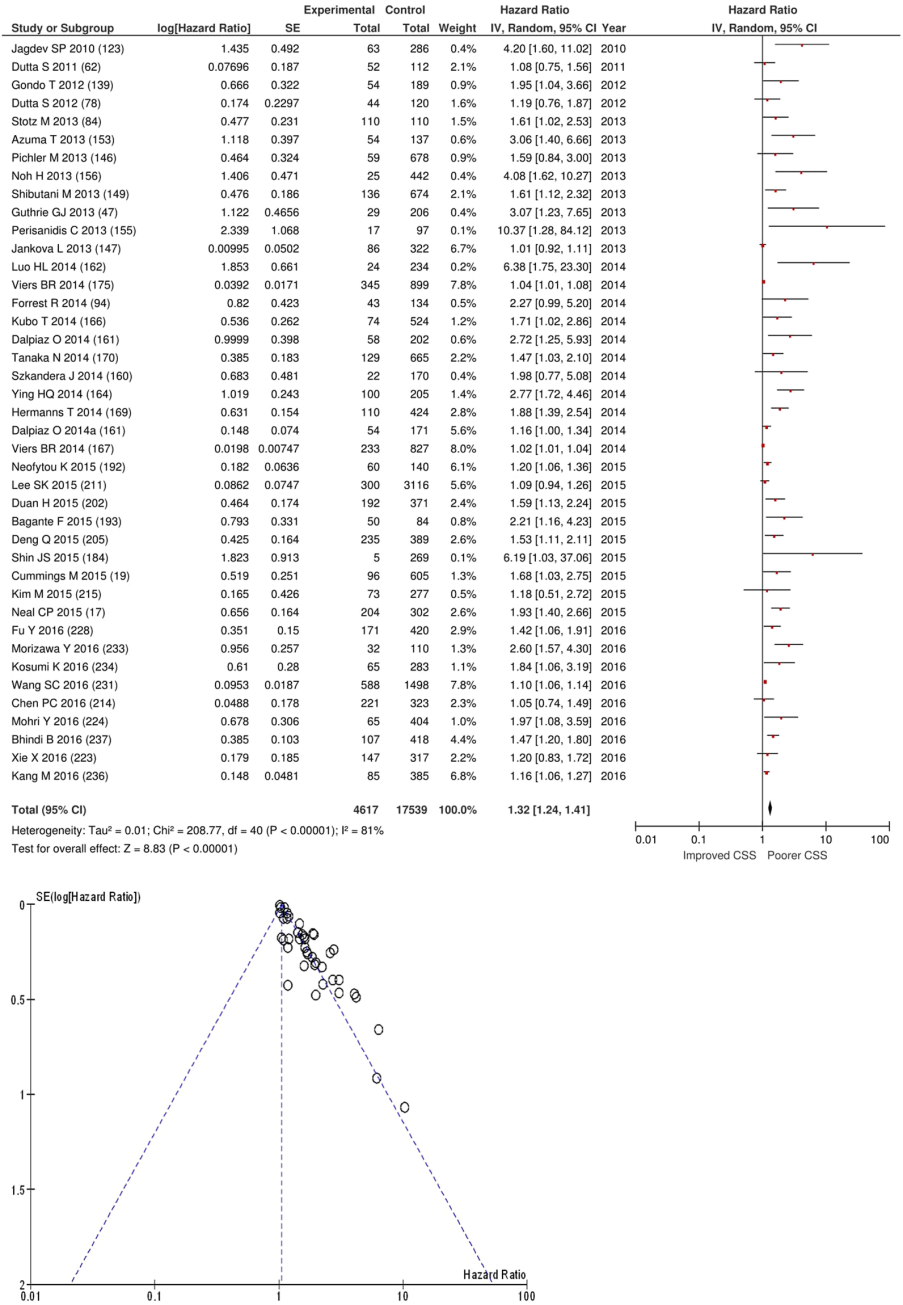
Forrest and Funnel Plot of Studies investigating the prognostic value of NLR in terms of CSS in an unselected cohort of patients with operable cancer.

After exclusion forty one studies examined the relationship with cancer specific survival including 17,539 patients (4,617 deaths), as its primary outcome measure. On meta-analysis there was a significant association between NLR and cancer specific survival (HR 1.32 95% CI 1.24–1.41, p < 0.00001) with a considerable degree of heterogeneity (I^2^ = 81%, Fig. 18). The most common NLR thresholds used was ≥5 (n = 7), ≥3 (n = 6) and NLR as continuous variable (n = 5). Other thresholds did not have more than four studies and therefore meta-analysis was not carried out (n = 19).

**Figure 19:**
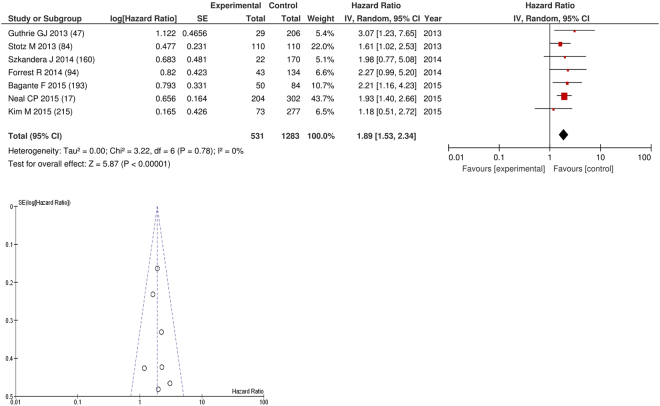
Forrest and Funnel Plot of Studies investigating the prognostic value of NLR ≥5 in terms of CSS in an unselected cohort of patients with operable cancer.

On meta-analysis those studies with a threshold of ≥5 (n = 7), including 1,283 patients (531 deaths) there was a significant association between elevated NLR and cancer specific survival (HR: 1.89 95% CI 1.53–2.34, p < 0.00001) with minimal heterogeneity (I^2^ = 0%, Fig. 19). These included colorectal (n = 2), liver only colorectal metastases (n = 1) and soft tissue sarcoma (n = 1), adrenal (n = 1), pancreatic (n = 1) and renal cancers (n = 1). In these seven studies, there was a variation in their geographical locations including the UK (n = 3), Austria (n = 2), US (n = 1) and South Korea (n = 1). The proportion of patients who had an NLR ≥5 was 19% in the UK, 35% in US and 7% in South Korea. No tumour site had more than four studies and therefore no further meta-analysis was carried out.

**Figure 20:**
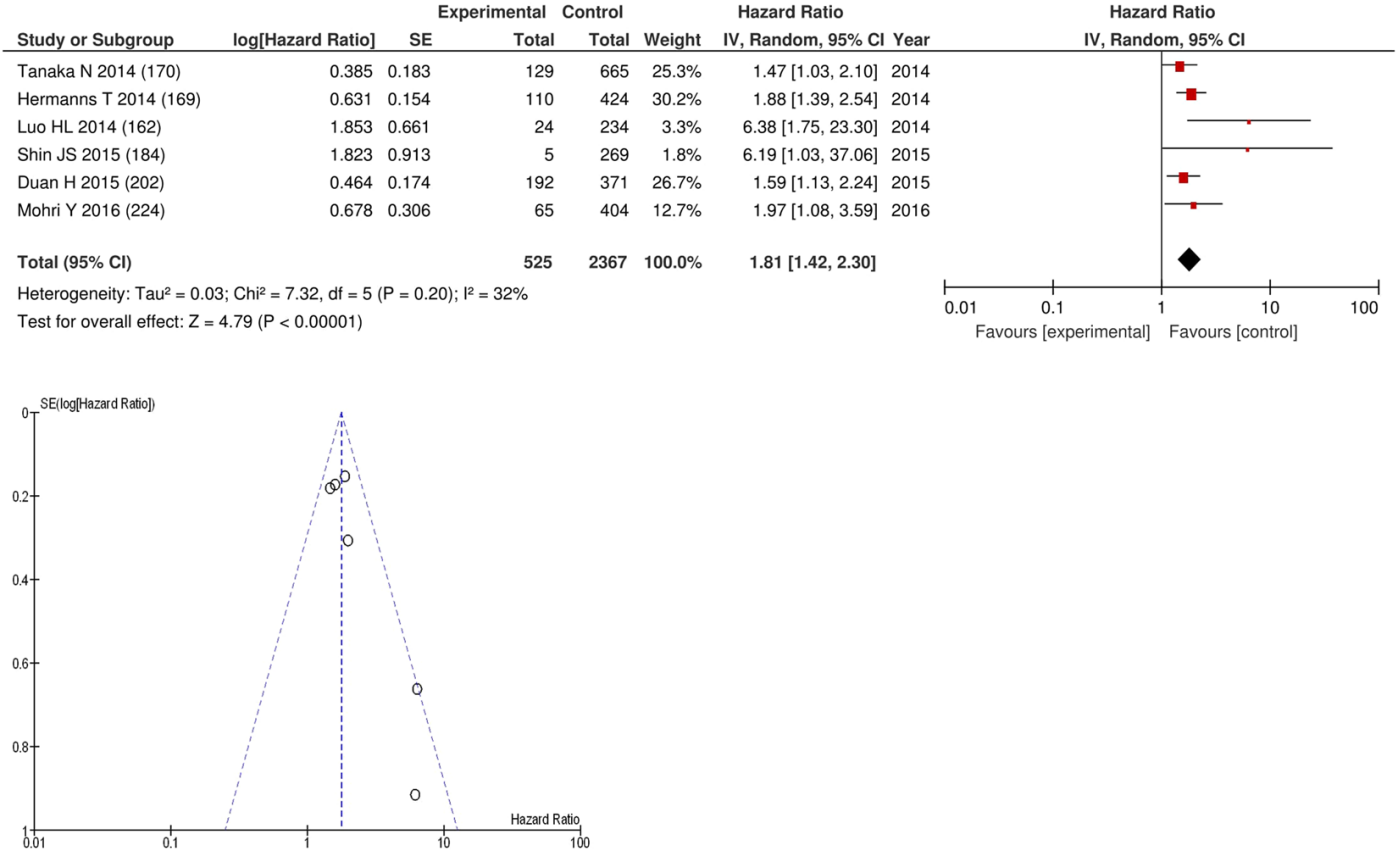
Forrest and Funnel Plot of Studies investigating the prognostic value of NLR ≥3 in terms of CSS in an unselected cohort of patients with operable cancer.

On meta-analysis those studies with a threshold of ≥3 (n = 6), including 2,367 patients (525 deaths) there was a significant association between elevated NLR and cancer specific survival (HR: 1.81 95% CI 1.42–2.30, p < 0.00001) with a moderate degree of heterogeneity (I^2^ = 32%, Fig. 20). These included renal (n = 2), bladder (n = 1), colorectal (n = 1), oesophageal (n = 1) and gastric cancers (n = 1). In these six studies, there was a variation in their geographical locations including Japan (n = 2), Korea (n = 1), China (n = 1), Taiwan (n = 1) and Canada (n = 1). The proportion of patients who had an NLR ≥3 was 25% in Japan, 20% in Korea, 20% in China, 40% in Taiwan and 51% in Canada. No tumour site had more than four studies and therefore no further meta-analysis was carried out.

**Figure 21:**
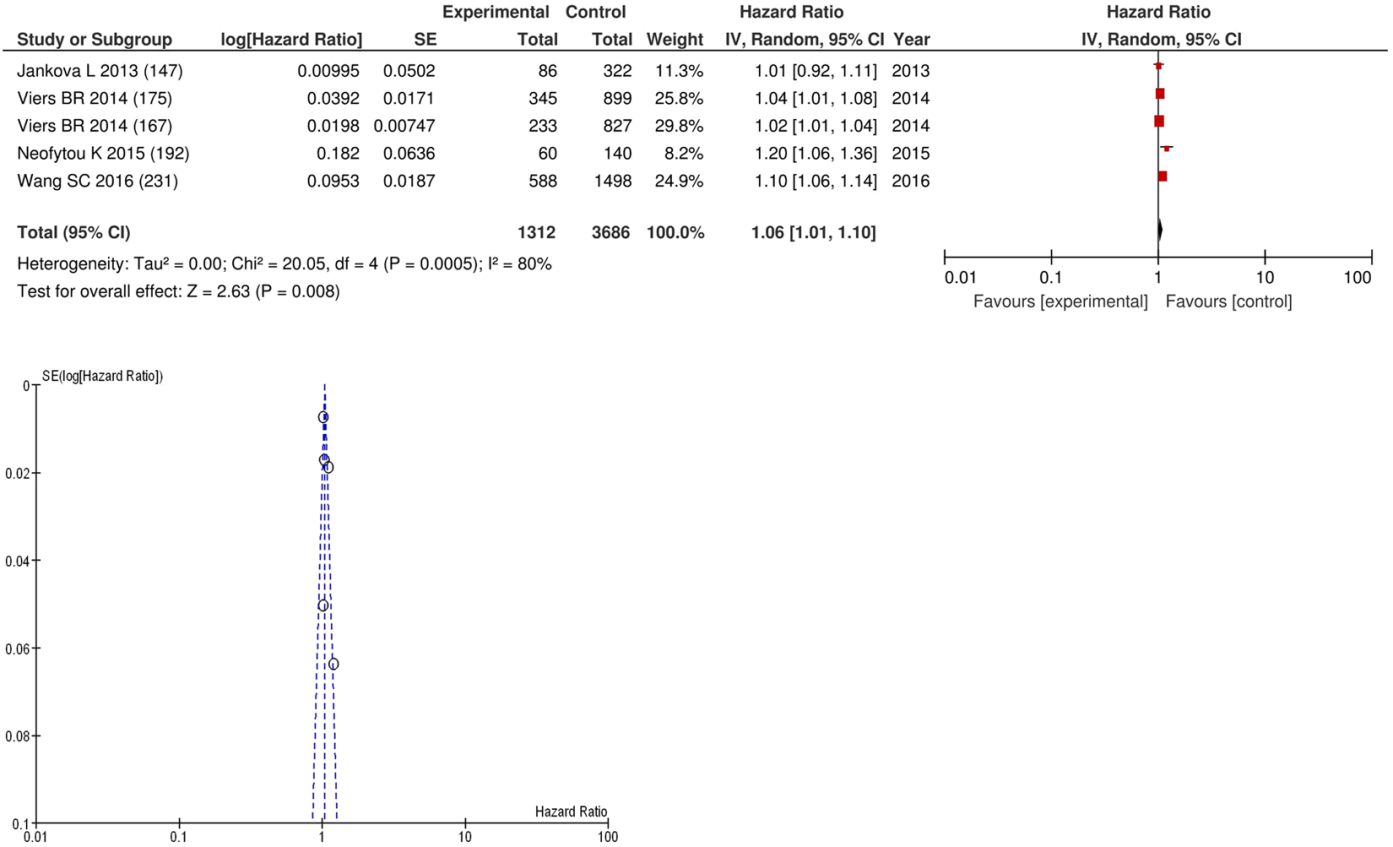
Forrest and Funnel Plot of Studies investigating the prognostic value of NLR as a continuous variable in terms of CSS in an unselected cohort of patients with operable cancer.

On meta-analysis those studies with NLR as continuous variable (n = 5), including 3,686 patients (1,312 deaths) there was a significant association between elevated NLR and cancer specific survival (HR: 1.06 95% CI 1.01–1.10, p = 0.008) with a substantial degree of heterogeneity (I^2^ = 80%, Fig. 21). These included renal (n = 1), bladder (n = 1), colorectal (n = 1), liver only colorectal metastases (n = 1) and gastric cancers (n = 1). In these six studies, there was a variation in their geographical locations including the US (n = 3), the UK (n = 1) and Australia (n = 1). No tumour site had more than four studies and therefore no further meta-analysis was carried out.

### Studies of the prognostic value of platelet lymphocyte ratio (PLR) in patients with primary operable cancer

Sixty eight articles with both OS and/or CSS as their primary outcome measures were identified (Supplementary Table). This comprised data on 29,273 patients (10,729 deaths) reporting the significant prognostic value of PLR in cohorts of patients with primary operable cancer (Supplementary Table). All sixty eight studies were conducted in a retrospective manner. Forty three studies were conducted in a multivariate and twenty five in a univariate manner (Supplementary Table).

**Figure 22:**
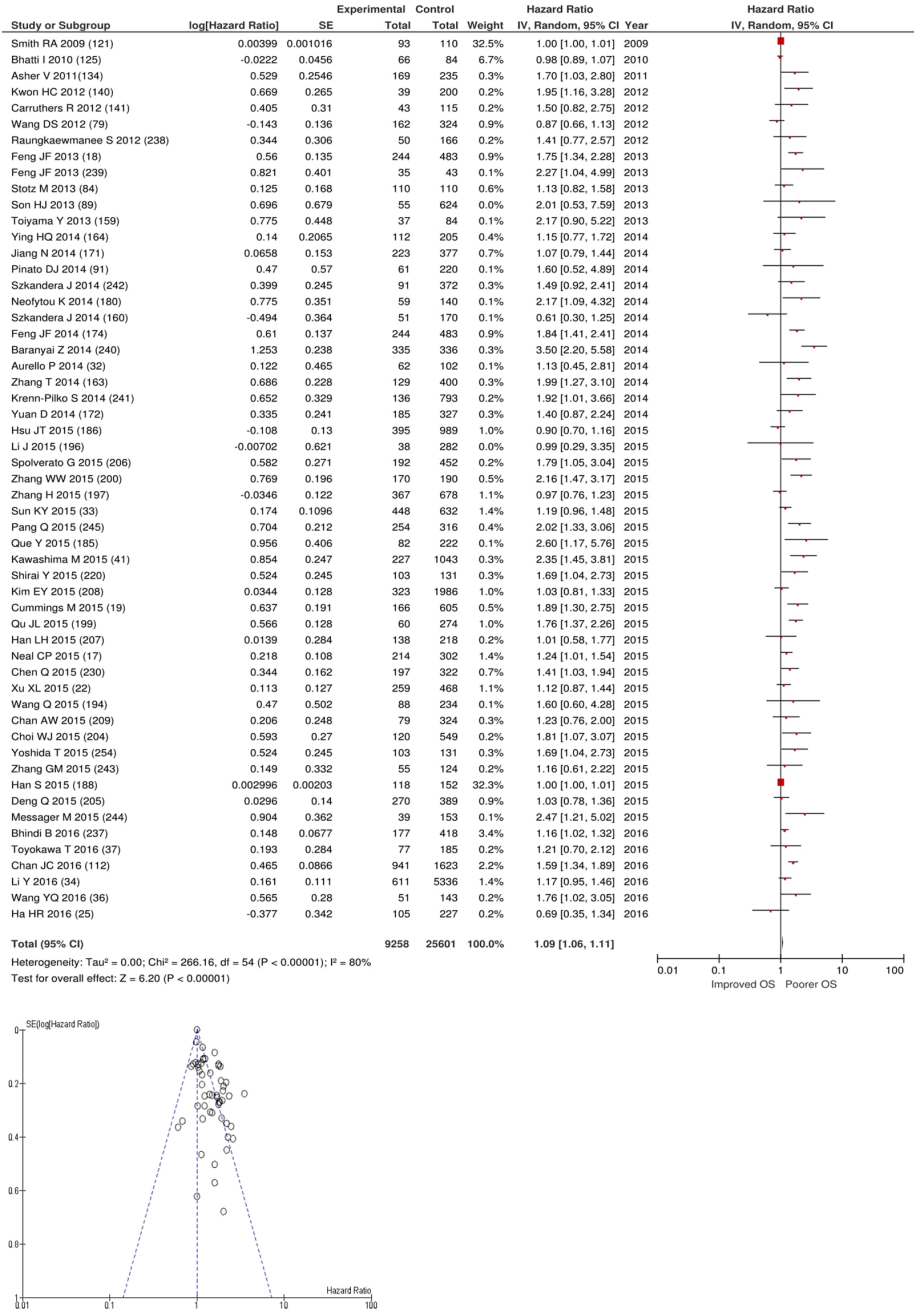
Forrest and Funnel Plot of Studies investigating the prognostic value of PLR in terms of OS in an unselected cohort of patients with operable cancer.

After exclusions fifty five studies examined the relationship with overall survival including 25,601 patients (9,258 deaths), as the primary outcome measure. On meta-analysis there was a significant association between an elevated PLR and overall survival (HR 1.09 95% CI 1.06–1.11, p < 0.00001) with a substantial degree of heterogeneity (I^2^ = 80%, Fig. 22). The most common PLR thresholds examined were ≥300 (n = 10) and ≥150 (n = 7). Other thresholds did not have more than four studies and therefore meta-analysis was not carried out (n = 58).

**Figure 23:**
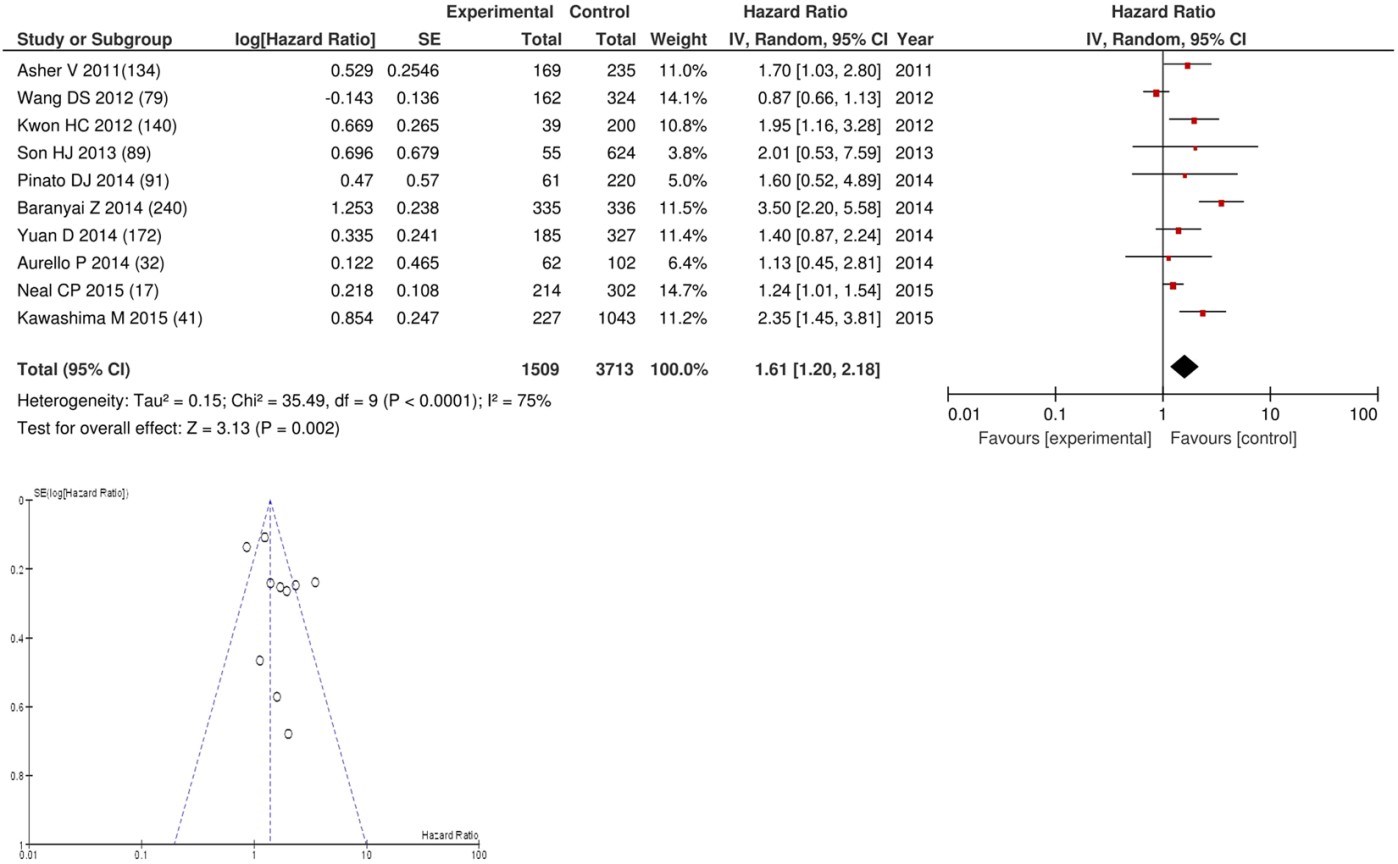
Forrest and Funnel Plot of Studies investigating the prognostic value of PLR ≥300 in terms of OS in an unselected cohort of patients with operable cancer.

On meta-analysis those studies with a threshold of ≥300 (n = 10), including 3,713 patients (HR: 1.61 95% CI 1.20–2.18, p = 0.002) with a substantial degree of heterogeneity (I^2^ = 75%, Fig. 23). These included colorectal (n = 3), lung (n = 2), gastric (n = 2), colorectal liver metastases (n = 1), oesophageal (n = 1) and ovarian cancers (n = 1). In these ten studies, there was a variation in their geographical locations including the UK (n = 3), Korea (n = 2), China (n = 2), Hungary (n = 1), Italy (n = 1) and Japan (n = 1). The proportion of patients who had a PLR ≥300 was 20% in the UK, 4% in Korea, 10% in China, 13% in Italy and 5% in Japan. No tumour site had more than four studies and therefore no further meta-analysis was carried out.

**Figure 24:**
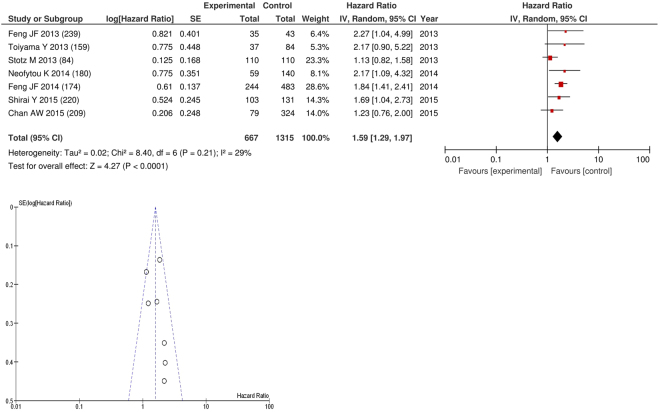
Forrest and Funnel Plot of Studies investigating the prognostic value of PLR ≥150 in terms of OS in an unselected cohort of patients with operable cancer.

On meta-analysis those studies with a threshold of ≥150 (n = 7), including 1,315 patients (667 deaths) there was a significant association between elevated PLR and overall survival (HR: 1.59 95% CI 1.29–1.97, p < 0.0001) with a minimal degree of heterogeneity (I^2^ = 29%, Fig. 24). These included oesophageal (n = 2), pancreatic (n = 2), liver (n = 1), colorectal liver metastases (n = 1) and colorectal cancers (n = 1). In these seven studies, there was a variation in their geographical locations including China (n = 2), Japan (n = 2), the UK (n = 1), Hong Kong (n = 1) and Australia (n = 1). The proportion of patients who had a PLR ≥150 was 43% in China, 49% in Japan, 41% in the UK, 27% in Hong Kong and 75% in Australia. No tumour site had more than four studies and therefore no further meta-analysis was carried out.

**Figure 25:**
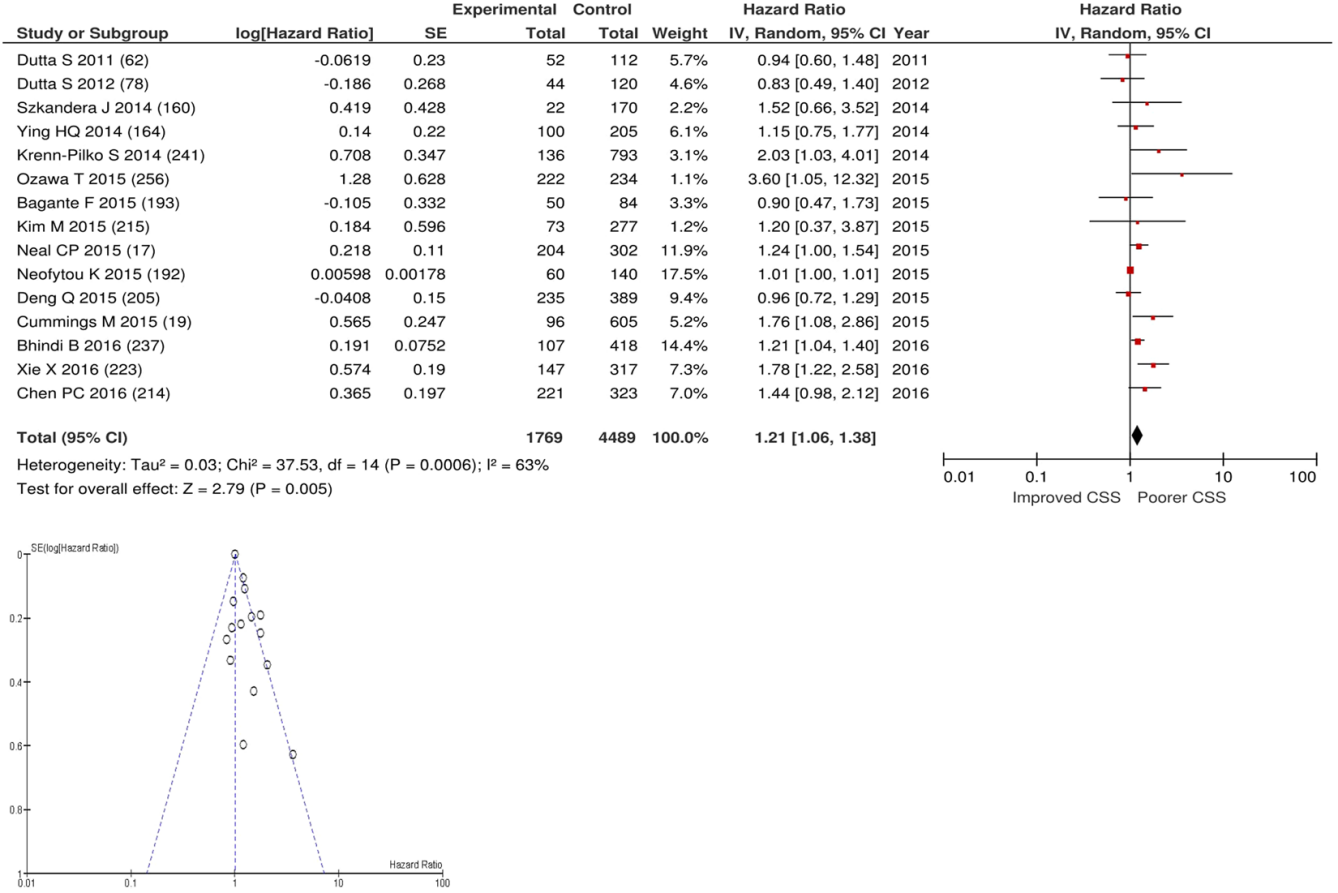
Forrest and Funnel Plot of Studies investigating the prognostic value of PLR in terms of CSS in an unselected cohort of patients with operable cancer.

After exclusions fifteen studies examined the relationship with cancer specific survival including 4,489 patients (1,769 deaths), as the primary outcome measure. On meta-analysis there was a significant association between an elevated PLR and cancer specific survival (HR 1.21 95% CI 1.06–1.38, p = 0.005) with a substantial degree of heterogeneity (I^2^ = 63%, Fig. 25). The most common PLR threshold examined was ≥300 (n = 4). Other thresholds used were >150 (n = 1), ≥25.4 (n = 1), >103 (n = 1), ≥132 (n = 1), ≥176 (n = 1), >190 (n = 1), ≥200 (n = 1), ≥240 (n = 1), ≥292 (n = 1), PLR as continuous variable (n = 1) and PLR per 100 units (n = 1). These included studies on oesophageal (n = 3), colorectal (n = 3), gastric (n = 2), colorectal liver metastases (n = 1), adrenal (n = 1), renal (n = 1), endometrial (n = 1), bladder (n = 1), soft tissue sarcoma (n = 1) and breast cancers (n = 1). Geographically studies were located in the UK (n = 5), China (n = 4), Austria (n = 2), Japan (n = 1), US (n = 1), South Korea (n = 1) and Canada (n = 1). The proportion of patients who had an elevated PLR was 12% in the UK, 55% in China, 23% in Japan, 38% in US and 3% in South Korea. No specific PLR thresholds had more than four studies and therefore no further meta-analysis was carried out.

### Studies of the prognostic value of lymphocyte monocyte ratio (LMR) in patients with primary operable cancer

Twenty one articles with both OS and/or CSS as their primary outcome measures were identified (Supplementary Table). This comprised data on 15,386 patients (4,298 deaths) reporting the significant prognostic value of LMR in cohorts of patients with primary operable cancer (Supplementary Table). All 21 studies were retrospective. Nineteen studies used multivariate and two used univariate survival analysis (Supplementary Table).

**Figure 26:**
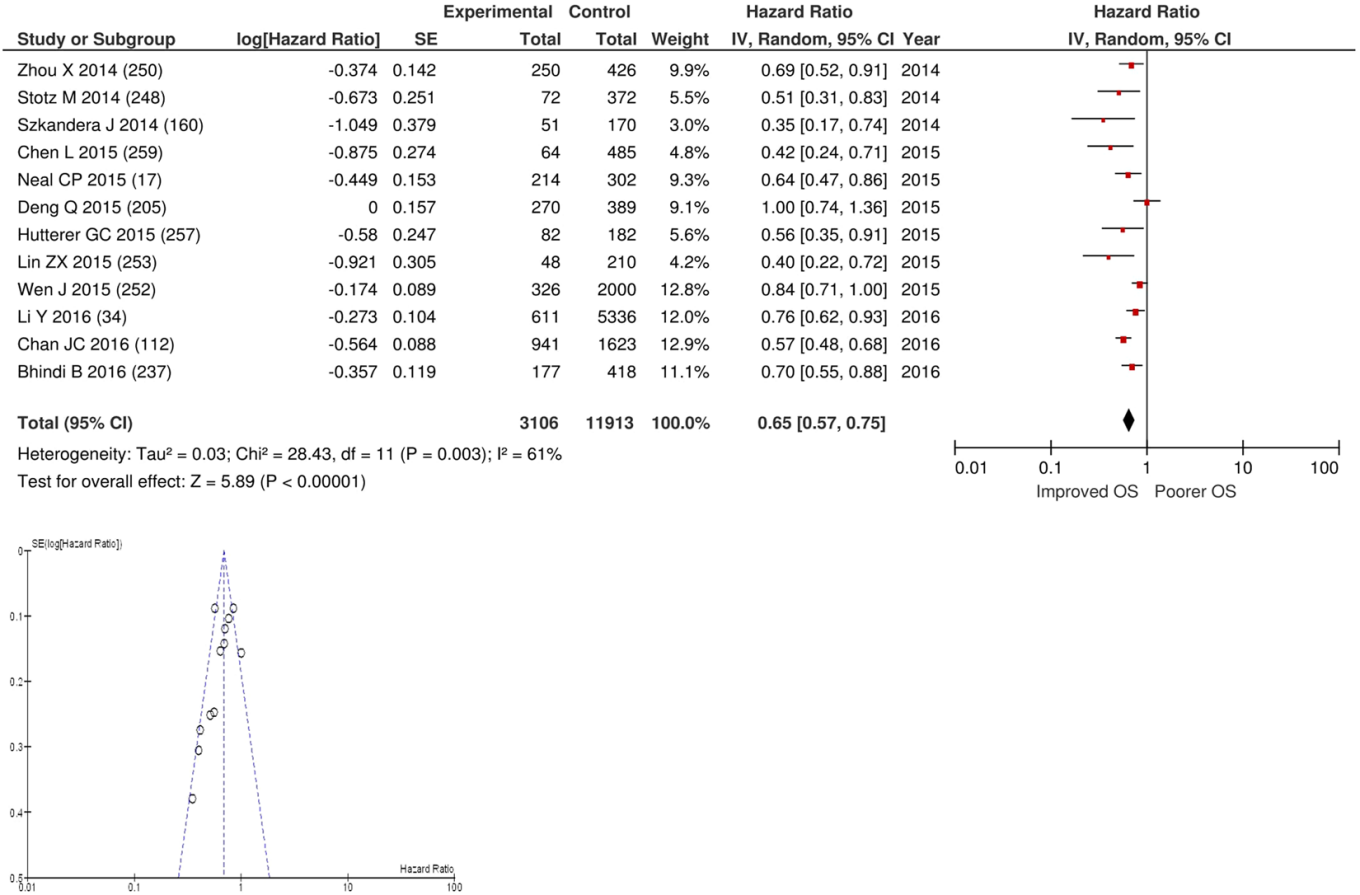
Forrest and Funnel Plot of Studies investigating the prognostic value of LMR in terms of OS in an unselected cohort of patients with operable cancer.

After exclusion twelve studies examined the relationship with overall survival including 11,913 patients (3,106 deaths), as the primary outcome measure. On meta-analysis there was a significant association between a elevated LMR and overall survival (HR 0.69 95% CI 0.63–0.74, p < 0.00001) with a substantial degree of heterogeneity (I^2^ = 61%, Fig. 26). There was a variety of LMR cut-offs used in each study including ≥2, (n = 1), ≥2.14 (n = 1), >2.35 (n = 1), >2.38 (n = 1), >2.83 (n = 1), ≥2.85 (n = 1), >2.87 (n = 1), >3.23 (n = 1), ≥3.80 (n = 1), ≥4 (n = 1), ≥4.32 (n = 1) and ≥4.95 (n = 1). These included studies on colorectal (n = 3), bladder (n = 2), liver only colorectal metastases (n = 1), gastric (n = 1), renal (n = 1), liver (n = 1), breast (n = 1), soft tissue sarcoma (n = 1) and cervical cancers (n = 1). Geographically the studies were carried out in China (n = 6), Austria (n = 3), the UK (n = 1), Canada (n = 1) and Australia (n = 1). The proportion of patients who had high LMRs was 71% in China, 68% in Japan, 64% in the UK, 49% in Australia and 48% in Austria. No specific LMR thresholds had more than four studies and therefore no further meta-analysis was carried out.

**Figure 27:**
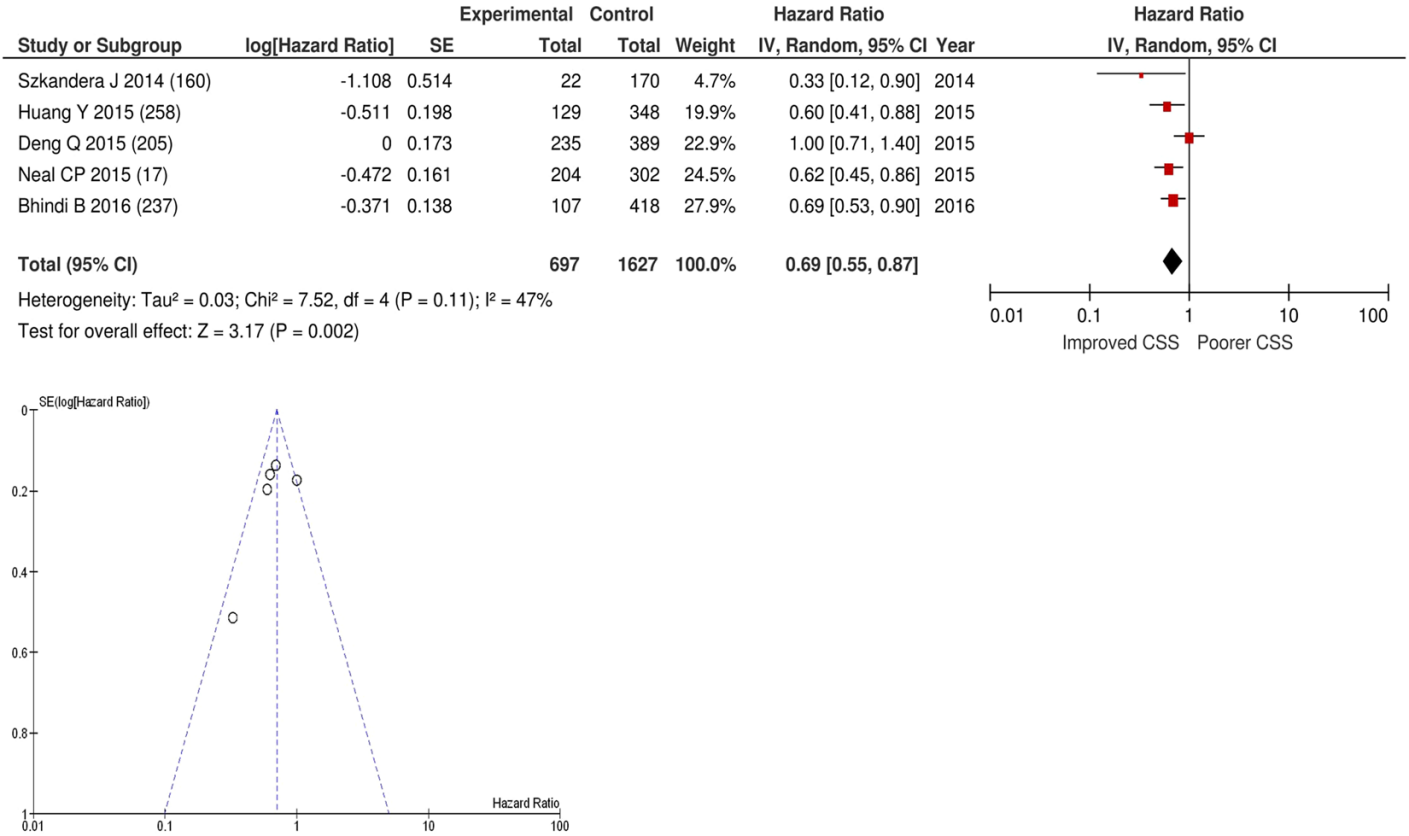
Forrest and Funnel Plot of Studies investigating the prognostic value of LMR in terms of CSS in an unselected cohort of patients with operable cancer.

After exclusion five studies examined the relationship with cancer specific survival including 1,627 patients (697 deaths), as the primary outcome measure. On meta-analysis there was a significant association between a elevated LMR and cancer specific survival (HR 0.70 95% CI 0.60–0.82, p < 0.00001) with a moderate degree of heterogeneity (I^2^ = 47%, Fig. 27). There was a variety of LMR cut-offs used in each study including >2.35 (n = 1), ≥2.85 (n = 1), >2.93 (n = 1) and ≥4.95 (n = 1). One study expressed LMR in terms of log. These included studies on liver only colorectal metastases (n = 1), gastric cancer (n = 1), oesophageal cancer (n = 1), bladder cancer (n = 1) and soft tissue sarcoma (n = 1). Geographically the studies were carried out in the China (n = 2), UK (n = 1), Austria (n = 1), and Canada (n = 1). The proportion of patients who had high LMRs was 68% in Japan, 64% in the UK, 50% in Austria and 40% in China. No specific LMR thresholds had more than four studies and therefore no further meta-analysis was carried out.

### Studies of the prognostic value of other scores of the systemic inflammatory response in patients with primary operable cancer

Thirty five articles reported a variety of other scores reported in less than 10 studies each. These included the PNI (Prognostic Nutritional Index), COP-NLR (combined platelet count and NLR), NLR/PLR combination, CAR (CRP/albumin ratio), SI (systemic inflammatory score), SII (systemic inflammatory index), NLR/CRP combination, (HALP) haemoglobin, albumin, lymphocyte and platelet, NLR/ESR (erythrocyte sedimentation rate) combination, (WLR) white cell count to lymphocyte count ratio, (APRI) AST-platelet ratio index, PI/CRP/WCC combination, Canton score, (AGR) albumin/globulin ratio, CRP/Neutrophil combination, (PIS) Prognostic Inflammation Score, and the CONUT score.

Eight articles with both overall survival (OS) and/or cancer specific survival (CSS) as their primary outcome measures were identified (Supplementary Table). This comprised data on 2,666 patients (1,387 deaths) reporting the significant prognostic value of PNI in cohorts of patients with primary operable cancer. All eight studies were carried out in a retrospective manner (Supplementary Table). Six studies used multivariate and two used univariate survival analysis (Supplementary Table).

**Figure 28:**
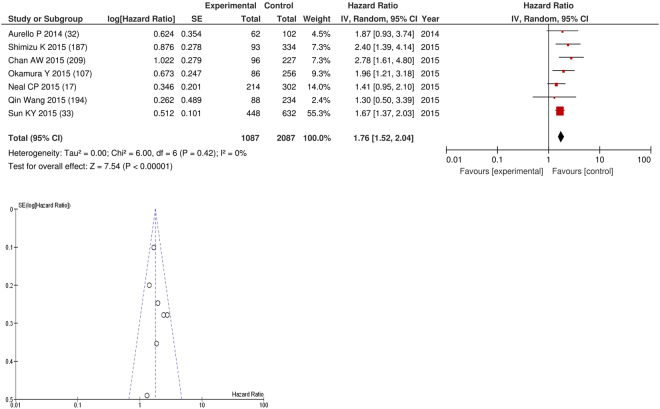
Forrest and Funnel Plot of Studies investigating the prognostic value of PNI in terms of OS in an unselected cohort of patients with operable cancer.

After exclusion seven studies examined the relationship with overall survival including 2,087 patients (1,087 deaths), as the primary outcome measure. On meta-analysis there was a significant association between PNI and overall survival (HR 1.76 95% CI 1.52–2.04, p < 0.00001) with minimal heterogeneity (I^2^ = 0%, Fig. 28). The most common PNI threshold examined was ≤45 (n = 3), ≤50 (n = 1), ≤50.5 (n = 1), 48.5 (n = 1), 48.2 (n = 1). These included hepatocellular (n = 3), gastric (n = 2), lung (n = 1) and colorectal liver metastases (n = 1). In these eight studies, there was a variation in their geographical locations including Japan (n = 2), UK (n = 1), Hong Kong (n = 1), China (n = 1), US (n = 1) and Italy (n = 1).The proportion of patients who with an elevated PNI was 74% in Hong Kong, 59% in Japan, 59% in Italy, 52% in China and 17% in the UK. No tumour site had more than four studies and therefore no further meta-analysis was carried out. Two studies examined the relationship with cancer specific survival including 579 patients (300 deaths), as the primary outcome measure. Both of these studies used a PNI threshold of ≤45. No threshold was used in ≥4 studies and thus, meta-analysis was not carried out.

Four studies reported the COP-NLR score. The first such study was by Ishizuka and coworkers^14^ from Japan. In this multivariate survival analysis on patients with colorectal cancer, low COP-NLR was shown to be related to a statistically better cancer specific survival (OR: 0.464 95% CI 0.267–0.807 p = 0.007). The second such study was also by Ishizuka and coworkers^15^ from Japan. In this multivariate survival analysis on patients with gastric cancer, elevated COP-NLR was shown to be related to a statistically significant worse overall survival (HR: 1.781 95% CI 1.094–2.899 p = 0.020). The third such study was by Zhang and coworkers^16^ from China. In this multivariate survival analysis on patients with lung cancer, elevated COP-NLR was shown to be related to a statistically significant worse overall survival (HR: 1.810 95% CI 1.587–2.056 p < 0.001). The fourth such study was by Neal and coworkers^17^ from the UK. In this univariate survival analysis on patients with colorectal liver metastases, elevated COP-NLR was shown to be related to a statistically significant worse overall survival (HR: 1.230 95% CI 1.005–1.505 p = 0.045) and worse cancer specific survival (HR: 1.243 95% CI 1.003–1.541 p = 0.047).

Three studies reported the combination of the NLR and PLR. The first such study was by Feng and coworkers^18^ from China. The combination of NLR and PLR is collectively named the CNP. The CNP was calculated based on data obtained on the day of admission, where patients with both elevated NLR (>3.45) and PLR (>166.5) were allocated a score of 2, and patients showing one or neither were allocated a score of 1 or 0, respectively. In this multivariate survival analysis on patients with oesophageal cancer, CNP 1 or 2 was shown to be related to a statistically worse overall survival (HR: 1.964 95% CI 1.371–2.814 p < 0.001). The second such study was by Cummings and coworkers^19^ from the UK. In this multivariate survival analysis on patients with endometrial cancer, both high NLR and PLR was shown to be related to a statistically significant worse overall survival (HR: 2.54 95% CI 1.61–4.01 p < 0.001) and worse cancer specific survival (HR: 2.26 95% CI 1.24–4.13 p = 0.008). The third such study was by Chuan Li and coworkers^20^ from China. In this multivariate survival analysis on patients with liver cancer, elevated postoperative NLR-PLR was shown to be related to a statistically significant worse overall survival (HR: 2.894 95% CI 1.992–4.2 p < 0.001).

Two studies reported the CAR. The first such study was by Ishizuka and coworkers^21^ from Japan. In this multivariate survival analysis on patients with colorectal cancer, CAR >0.038 was shown to be related to a statistically worse overall survival (HR: 2.613 95% CI 1.621–4.212 p < 0.001). The second such study was by Xu and coworkers^22,23^ from China. In this multivariate survival analysis on patients with oesophageal cancer, CRP/Albumin ratio >0.50 was shown to be related to a statistically significant worse overall survival (HR: 2.44 95% CI 1.82–3.26 p < 0.0001).

One study reported the SI a score involving leucocyte count, serum albumin and haemoglobin level. High leucocyte count (>9,500 µl), low serum albumin level (3.5 g/dl) and low haemoglobin level (<12.5 mg/dl) was each allocated a score of 1.The study was conducted by Miyata and coworkers^24^ from Japan. In this multivariate survival analysis on patients with oesophageal cancer, SI score of 2/3 was shown to be related to a statistically significant worse overall survival (HR: 3.17 95% CI 1.74–5.78 p = 0.0002).

One study reported on the SII which was determined as neutrophil × platelet/lymphocyte. The study was conducted by Ha and coworkers^25,26^ from South Korea. In this multivariate survival analysis on patients with ampulla of vater cancer, SII ≤780 was shown to predict better overall survival (HR: 0.924 95% CI 0.44–1.93 p = 0.833).

One study reported on the combination of the NLR and CRP. The study was conducted by Tomita and coworkers^27^ from Japan. In this multivariate survival analysis on patients with lung cancer, low NLR and low CRP (compared to both high) was shown to predict better overall survival (RR: 0.403 95% CI 0.240–0.689 p = 0.0012).

One study reported on preoperative HALP. The study was conducted by Chen and coworkers^28^ from China. In this multivariate survival analysis on patients with gastric cancer, HALP ≥56.8 was shown to predict better overall survival (HR: 0.700 95% CI 0.496–0.987 p = 0.042).

One study reported on the combination of the NLR and ESR. The study was conducted by Hyun and coworkers^29^ from Korea. Patients were divided into three groups: those with ESR and NLR in the normal range (group 0), those with either elevated ESR or elevated NLR (group I), and those with both elevated ESR and elevated NLR (group II). In this multivariate survival analysis on patients with renal cancer, both elevated ESR and NLR was shown to predict worse overall survival (HR: 3.521 95% CI 1.888–6.567 p < 0.001) and worse cancer specific survival (HR: 4.367 95% CI 1.987–9.597 p < 0.001).

One study reported on the WLR. The study was conducted by East and coworkers^30^ from the UK. In this multivariate survival analysis on patients with colon cancer, WLR ≥3.4 was shown to predict worse overall survival (HR: 4.10 95% CI 3.13–7.42 p = 0.03).

One study reported on the APRI. The study was conducted by Shen and coworkers^31^ from China. In this multivariate survival analysis on patients with liver cancer, APRI ≥0.62 was shown to predict worse overall survival (HR: 1.508 95% CI 1.127–2.016 p = 0.006).

One study reported on the combination of the PI, CRP and white cell count (0 if both low, 1 if either high, 2 if both high). The study was conducted by Aurello and coworkers^32^ from Italy. In this multivariate survival analysis on patients with gastric cancer, PI 2 was shown to predict worse overall survival (HR: 0.37 95% CI 0.16–0.82 p = 0.01).

One study reported on the Canton score involving PNI, NLR and platelet. The study was conducted by Sun and coworkers^33^ from China. In this multivariate survival analysis on patients with gastric cancer, elevated Canton score was shown to predict worse overall survival (HR: 1.643 95% CI 1.142–2.364 p = 0.007).

One study reported on the AGR. The study was conducted by Li and coworkers^34^ from China. In this multivariate survival analysis on patients with colorectal cancer, AGR ≥1.50 was shown to predict better overall survival (HR: 0.646 95% CI 0.543–0.767 p < 0.001).

One study reported on the combination of CRP and neutrophils. The study was conducted by Christina and coworkers^35^ from Austria. In this multivariate survival analysis on patients with oral cancer, high CRP/ neutrophil was shown to predict worse overall survival (HR: 2.7 95% CI 0.68–10.75 p = 0.16).

One study reported on the PIS involving a combination of NLR and serum albumin. PIS was defined as follows: patients with increased NLR and decreased serum albumin were assigned score 0; patients with either increased NLR or decreased serum albumin were assigned score 1; patients with decreased NLR and increased serum albumin were assigned score 2. The study was conducted by Wang and coworkers^36^ from China. In this multivariate survival analysis on patients with ovarian cancer, PIS 2 was shown to predict better overall survival (HR: 0.18 95% CI 0.09–0.38 p < 0.001).

Finally, the last study reported on the CONUT score involving serum albumin concentration, total lymphocyte count and total cholesterol concentration. The study was conducted by Toyokawa *and coworkers* ^37^ from Japan. In this multivariate survival analysis on patients with oesophageal cancer, high CONUT score was shown to predict worse overall survival (HR: 2.303 95% CI 1.191–4.455 p = 0.013).

### Assessment of bias using funnel plot analysis of studies carried out in patients with primary operable cancer

Funnel plot analysis containing ten or more studies revealed bias towards studies reporting a relationship between an increased SIR as evidenced by the GPS/GPS (multiple tumour types Figs 2 and 8; colorectal cancer Figs 3 and 9), NLR (multiple tumour types Figs 10 and 18; NLR >5 Fig. 11), PLR (multiple tumour types Figs 22 and 25; PLR >300 Fig. 23), LMR (multiple tumour types Fig. 26) and poorer survival. The funnel plots also showed that a clear majority of studies had high patient numbers. This is particularly true for studies focusing on GPS/mGPS (Figs 2 and 8), NLR (Figs 10 and 18), PLR (Fig. 22) and LMR (Fig. 26).

## Discussion

In the present review 244 reports of the prognostic value of systemic inflammation based prognostic scores were identified. This is in contrast to the initial review by Roxburgh and McMillan (2010) where 18 such studies were identified. In particular, those scores based on the ratio of components of a white cell count have been the subject of intense interest with, over the intervening 7 years, 158 studies reporting the value of the NLR, 68 reporting PLR and 21 reporting LMR. Also, the cumulative GPS/mGPS has been the subject of 80 reports. The majority of these studies have been carried out in lung and gastrointestinal cancer. For example, the GPS/mGPS had prognostic value in lung (5 studies), gastric cancer (7 studies), pancreatic (5 studies), and colon cancer (3 studies). A feature of this up to date review of systemic inflammation based prognostic scores is the identification of the proliferation of new scores derived from routinely available markers of the SIR. Most notable among these that have been validated in several studies are PINI (7 studies), COP-NLR (4 studies) and CNP (3 studies). It remains to be established whether any of the scores will have prognostic value in addition to the GPS/mGPS and NLR. Irrespective, there is increasing recognition and acceptance of the clinical utility of systemic inflammation based prognostic scores prior to surgery for cancer.

It is perhaps surprising that, given apparent the superior prognostic value of the GPS/ mGPS^3^ the relatively larger numbers of reports of the prognostic value of ratios based on components of the white cell count. However, the pre-operative differential white cell count is part of the standard pre-operative workup for the majority of cancer resections as it is used to help identify patients who may have an infection prior to surgery. Also, the white cell count is used to identify any pre-existing conditions that may affect the surgical procedure such as the hypercoagulability of thrombocytosis. Thus, these results are routinely available for retrospective studies. This might also explain the variety of prognostic thresholds reported for NLR, PLR and LMR. In contrast, reports on the prognostic value of the GPS/mGPS, not routinely assessed as part of the standard pre-operative workup, were more likely to be examined in prospective studies. This might explain the consistent adherence to the original thresholds reported for GPS/ mGPS. From the above there is a strong case for the GPS/mGPS to be incorporated into pre-operative workup of patients undergoing surgery for cancer.

It is of interest that while there is general uniformity of thresholds used in the GPS/mGPS studies, with most adhering to the original abnormal thresholds (CRP >10 mg/l and albumin <35 g/l), studies in East Asia particularly Japan have used thresholds of 7.5 mg/l^38^, 5 mg/l^39,40^ and 3 mg/l^41–43^. Such lower CRP thresholds are above the normal reference ranges in Japan/ East Asia cohorts and results in fewer patients breaching the CRP >10 mg/l threshold. This observation of a greater proportion of patients with elevated systemic inflammation markers in Western countries compared with Eastern Asian countries is also apparent in white cell derived ratios. Given the objective and reproducible nature of systemic inflammation based prognostic scores it is likely that such observations are real. Indeed, there are recognized ethnic differences in the normal range of neutrophils and lymphocytes^44–46^. For example, Azab and co-workers recently reported that, in more than 9,000 patients in the United States, there were ethnic differences in the NLR^46^. Specifically, in the cohort as a whole the mean NLR was 2.15. In contrast, black Americans had a mean NLR of 1.76, Hispanic Americans had a mean NLR of 2.08 and white Americans had a mean NLR of 2.24^46^. Also, within ethnicities, patients who had diabetes, cardiovascular disease, a high BMI and were smokers had a significantly higher NLR^46^. Although, similar data for the GPS/mGPS has not yet appeared in the literature it is likely that there would be a similar effect on the GPS/mGPS. Therefore, given that the most common abnormal thresholds used for NLR are >5 and >3 it is likely that a combination of tumour and host genetic and environmental factors are responsible for such consistent East/West differences. These and the present results emphasise the importance of not only staging the tumour but also the host systemic inflammatory response in patients with operable disease^7^.

Recently, studies have directly compared the prognostic value of the two most common combined markers of the systemic inflammatory response, the NLR and the GPS/mGPS. Guthrie and coworkers^47^ reported a comparison in both the preoperative and follow-up settings in patients with resectable colorectal cancer. In this study of 206 patients undergoing a surgical resection at a single institution it was reported that both preoperative mGPS (HR: 1.97, CI 1.16–3.34, p < 0.005) and NLR (HR: 3.07, CI 1.23–7.63, p < 0.05) were independently associated with cancer specific survival^47^. However in the postoperative follow-up only mGPS (HR: 4.81, CI 2.13–10.83, p < 0.001) maintained its significance in terms of cancer specific survival^47^. In contrast, Wang and coworkers (2012) reported that, in 177 patients with pancreatic cancer treated with surgery and palliative chemotherapy, although NLR and mGPS predicted overall survival only NLR was independently associated with overall survival (HR: 2.54 CI 1.31–4.90, p = 0.006)^48^. Finally, Okuno and coworkers (2016) reported that, in 534 patients with perihilar cholangiocarcinoma, both the NLR and mGPS had prognostic value^49^. However, on multivariate analysis, only the mGPS was independently associated with overall survival (HR: 1.58 CI 1.21–2.06, p = 0.001)^49^.

The present review and meta-analysis has a number of limitations^50–261^. For example funnel plot analysis, even after fixed effect analysis, showed that there was for all systemic inflammation based prognostic scores some asymmetry. This would suggest that there may be some reporting bias. The basis of this bias is not clear. Other than statistically significant results being more likely to be published other possible contributors may be that the studies included in the analysis were English language only publication, had small study size, included multiple tumour types and included multiple thresholds. Nevertheless the consistency of prognostic value over a variety of systemic inflammation based prognostic scores and across larger studies, single tumour types and single thresholds would indicate that although there was evidence of bias in the meta-analysis, such scores do indeed have prognostic value. Similarly when only univariate analysis was available it was entered into the analysis. The majority of studies had HR derived from multivariate analysis (181 studies) and therefore harmonisation of HR results was not attempted. In the present meta-analysis there was considerable heterogeneity in the HR of some of the markers of the SIR. However, this was less when a consistent threshold for the marker was used. There are other potential contributors to such heterogeneity including geographical location. Such sub-analysis was limited by the number of studies available for meta-analysis. The strength of this present review is its comprehensive nature.

In summary, the results of this review consolidate the prognostic value of combined markers of the systemic inflammatory response including GPS/mGPS NLR, PLR and LMR in patients with resectable cancers. This is particularly true for the GPS/mGPS and NLR and in lung and GI cancers. These should form part of the routine preoperative workup and follow-up for all such patients undergoing resection for cancer.

## Electronic supplementary material

Supplementary Information
