# Supplementary material for: The role of the systemic inflammatory response in predicting outcomes in patients with operable cancer: Systematic review and meta-analysis
Source: Sci Rep. 2017 Dec 1;7:16717. doi: 10.1038/s41598-017-16955-5 (PMC5711862; doi:10.1038/s41598-017-16955-5)
Supplement: Supplementary file 1 — Supplementary Information [file 41598_2017_16955_MOESM1_ESM.pdf]

The role of the systemic inflammatory response in predicting outcomes in patients with operable cancer: Systematic review and meta-analysis

Ross D Dolan, Jason Lim, Stephen T McSorley, Paul G Horgan, Donald C McMillan

Academic Unit of Surgery, School of Medicine, University of Glasgow, Glasgow Royal Infirmary, Glasgow, UK

Corresponding author:

Ross Dolan, Clinical Research Fellow,

Academic Unit of Surgery, University of Glasgow,

New Lister Building, Glasgow Royal Infirmary,

Glasgow,

G4 0SF,

United Kingdom.

Email: Ross.Dolan@glasgow.ac.uk

Tel: 0141 211 4000

Fax: 0141 211 4943

Keywords: Systemic inflammation, Operable cancer, Glasgow Prognostic Score, Neutrophil Lymphocyte Ratio, Platelet Lymphocyte Ratio, Lymphocyte Monocyte Ratio, Cancer Specific Survival, Overall Survival.

Supplementary Tables:

Table 1: Studies investigating the prognostic value of GPS/mGPS in an unselected cohort of patients with operable cancer (Abbreviations: POSSUM (physiological and operative Severity Score for the enUmeration of Mortality and Morbidity), pLNR (positive lymph node ratio), TNM (tumour, node, metastasis), ACE-27 (adult co-morbidity evaluation), CLIP (cancer of liver Italian program), CD68 (cluster of differentiation), UISS (UCLA integrated staging system), SSIGN (stage, size, grade and necrosis), LN (lymph node), HS-mGPS (high sensitivity mGPS), BCLC (Barcelona clinic liver cancer), AJCC (American joint committee on cancer), F-NLR (Fibrinogen and NLR), LVI (lymphatic/venous invasion), IBS (Inflammatory based score), CONUT (controlling nutritional status)

No: GPS/ mGPS	Study	Type of Study	Cancer	Country	Patients (n)	Measure of SIR	Additional Treatment	Cancer deaths (n)	Overall deaths (n)	Cancer survival (HR, 95%CI)	Overall survival (HR, 95%CI)	Independent Prognostic Factors
1.	Ishizuka et al[50] 2007	Retrospective	Colorectal	Japan	315	GPS (0/1/2)	No neoadjuvant treatments given	66	144	N/A	Multivariate: OR: 0.165 (0.037-0.732) p=0.0177	Multivariate: Nil else
2.	McMillan et al[8] 2007	Retrospective	Colorectal	United Kingdom	316	mGPS (0/1/2)	Adjuvant therapy not specified	70	117	Univariate: Colon: p<0.0001 Rectal: p<0.0001 Multivariate: Dukes stage B 1.74 (1.20-2.51) p=0.0032	Univariate: Colon: p<0.0001 Rectal: p<0.0001	Multivariate: Age
3.	Leitch et al[51] 2007	Retrospective	Colorectal	United Kingdom	149	mGPS (0/1/2)	43 patients in the GPS 0, 24 patients in the GPS1 and 4 patients in the GPS 4 group underwent adjuvant treatment	20	45	Multivariate: 2.21 (1.11-4.41) p=0.024	Multivariate: 2.08 (1.32-3.28) p=0.002	Multivariate: Age, TNM stage, monocyte count
4.	Kobayashi et al[52] 2008	Retrospective	Oesophageal squamous cell carcinoma	Japan	48	GPS (0/ 1 and 2)	Neoadjuvant chemoradiotherapy (nCRT)	N/A	34	N/A	Multivariate: OR: 0.17 (0.06-0.52) p=0.019	Multivariate: Nil else
5.	Roxburgh et al[53] 2009	Retrospective	Colorectal	United Kingdom	287	mGPS (0/1/2)	Adjuvant therapy not specified	67	116	Multivariate: 2.65 (1.66-4.25) p<0.001	N/A	Multivariate: Age, Dukes stage, Klintrup criteria

6.	Ishizuka et al[54]<br><br>2009	Retrospective	Colorectal Liver Metastases	Japan	93	GPS (0/1/2)	No patients had neoadjuvant chemotherapy	48	51	Univariate:<br>OR: 1.273 (0.269-6.030)<br>p=0.7612	N/A	Multivariate:<br>Number of tumours, number of hepatectomies, synchronous lung metastasis, CRP
7.	Crozier et al[55]<br><br>2009	Prospective	Colon cancer	UK	188	mGPS (0/1/2)	54 patients received adjuvant therapy	47	67	Multivariate: TNM stage 2 patients (n=95)<br>2.22 (1.04-4.74)<br>p=0.0391	N/A	Multivariate:<br>Presentation (elective/ emergency)
8.	Roxburgh et al[56]<br><br>2010	Retrospective	Colon	United Kingdom	287	mGPS (0/1/2)	Adjuvant chemotherapy	80	125	Multivariate:<br>1.96 (1.19-3.21)<br>p=0.008	Multivariate:<br>1.73 (1.18-2.25)<br>p=0.005	Multivariate:<br>Dukes stage, vascular invasion
9.	Richards et al[57]<br>2010	Prospective	Colorectal	United Kingdom	320	mGPS (0/1/2)	66 had adjuvant therapy	83	136	Multivariate:<br>1.78 (1.32-2.41)<br>p<0.001	Multivariate:<br>1.60 (1.26-2.02) p<0.001	Multivariate:<br>Age, Smoking, Dukes stage, POSSUM physiology score
10.	Hefler-Frischmuth et al[58]<br>2010	Prospective	Vulval	Austria	93	GPS (0/1/2)	No mention of adjuvant treatment	23	27	N/A	Multivariate:<br>1.1 (0.5–2.4) p=0.8	Multivariate:<br>Tumour stage, Positive lymph node
11.	Kobayashi et al[59]<br><br>2010	Retrospective	Esophageal Squamous Cell Carcinoma	Japan	65	GPS (0 and 1)	39 patients received neoadjuvant chemoradiotherapy	57	N/A	Multivariate:<br>GPS 0:<br>0.071 (0.011-0.470) p=0.0061<br><br>GPS 1:<br>0.367 (0.046-2.927) p=0.3442	N/A	Multivariate:<br>Number of lymph node metastases
12.	Kobayashi et al[60]<br><br>2010	Retrospective	Colorectal Liver Metastases	Japan	63	GPS (0/ 1 and 2)	53 patients received chemotherapy after hepatectomy	N/A	30 (5-year survival)	N/A	Multivariate:<br>3.07 (1.18-7.98)<br>p= 0.0217	Multivariate:<br>Liver metastases
13.	Moug et al[61]<br>2011	Retrospective	Colorectal	United Kingdom	206	GPS (0/1/2)	9 had neoadjuvant and 48 had adjuvant	N/A	63	N/A	Multivariate:<br>1.56 (1.18-2.08) p=0.02	Multivariate:<br>pLNR
14.	Dutta et al[62]<br>2011	Retrospective	Oesophageal	United Kingdom	112	GPS (0/1/2)	31 had neoadjuvant and 14 adjuvant therapy	52	59	Multivariate:<br>4.31 (2.20-8.45)<br>p<0.001	N/A	Multivariate:<br>Positive to total lymph node ratio (0/≤0.2/>0.2)

15.	Dutta et al[63] 2011	Retrospective	Oesophagogastric	United Kingdom	121	GPS (0/1/2)	67 patients have had neoadjuvant and 19 adjuvant therapy	39	44	Multivariate: 1.96 (1.09–3.54) p= 0.025	N/A	Multivariate: TNM stage
16.	Crumley et al[64] 2011	Retrospective	Gastroesophageal	United Kingdom	100	GPS (0/1/2)	Adjuvant and neoadjuvant therapy administered chemo and radiotherapy but numbers not given	51	55	Multivariate: 3.99 (1.96-8.11) p<0.001	N/A	Multivariate: Number of positive LN, Tumour differentiation, Klintrup score, Ki-67
17.	Jamieson et al[65] 2011	Prospective	Pancreatic Ductal Cancer	United Kingdom	135	GPS (0/1/2)	74 patients had adjuvant therapy	107	109	N/A	Multivariate: 2.26 (1.43-3.57) p=0.0001	Multivariate: Tumour stage, tumour grade, margin involved, venous invasion, preoperative biliary drainage, adjuvant therapy
18.	Roxburgh et al[66] 2011	Retrospective	Colorectal	United Kingdom	302	GPS (0/1/2)	71 patients had adjuvant therapy	85	135	Multivariate: 1.81 (1.32-2.48) p<0.001	Multivariate: 1.60 (1.25-2.05) p<0.001	Multivariate: Age, TNM, Peterson Index, Postoperative infective complications, ACE-27
19.	Vashist et al[67] 2011	Retrospective	Oesophageal	Germany	495	GPS (0/1/2)	No adjuvant or neoadjuvant therapy	N/A	71	N/A	Multivariate: GPS 1: 1.7 (1.3–2.2) p<0.001<br><br>GPS 2: 2.5 (1.7-3.6) p<0.001	Multivariate: Tumour size, Node status, Mets, Cell type
20.	Nozoe et al[68] 2011	Prospective	Gastric	Japan	232	GPS (0/1/2)<br><br>mGPS (0/1/2)	No mention of adjuvant treatment	N/A	184	N/A	Multivariate: GPS: 3.425 (1.211–9.709) p=0.020<br><br>mGPS: 4.184 (1.792-9.804) p=0.0009	Multivariate: Tumour stage
21.	Ishizuka et al[43] 2011	Retrospective	HCC	Japan	300	hGPS (0, 1/2)<br><br>*CRP>0.3 mg/dl	No mention of adjuvant treatment	91	106	N/A	Univariate: OR: 2.107 (1.061-4.185) p=0.033	Univariate: CLIP score (0,1/ ≥2)

22.	Roxburgh et al[69]<br><br>2011	Retrospective	Colon Cancer	UK	76	mGPS (0/1 or 2)	All patients received adjuvant chemotherapy	30	33	Multivariate:<br>3.24 (1.45-7.27)<br>p=0.004	Multivariate:<br>3.23 (1.49-7.01)<br>p=0.003	Multivariate:<br>Petersen index, T category
23.	Dutta et al[70]<br><br>2012	Retrospective	Oesophageal Cancer	United Kingdom	98	GPS (0/1/2)	47 underwent neoajuvant therapy and 18 adjuvant	60	68	Multivariate:<br>2.91 (1.51-5.62)<br>p=0.001	N/A	Multivariate:<br>Age, Positive to total lymph node ratio, CD68 tertiles
24.	Ishizuka et al[71]<br><br>2012	Retrospective	HCC	Japan	398	GPS (0, 1/2)	No mention of neoadjuvant or adjuvant therapy	112	130	N/A	Multivariate:<br>OR: 2.5 (1.124-5.561)<br>p=0.025	Multivariate:<br>CLIP score (0, 1/ ≥2)
25.	Richards[72]<br>2012	Retrospective	Colorectal Cancer	United Kingdom	343	GPS (0/1/2)	No mention of adjuvant treatment	85	N/A	Multivariate:<br>1.74 (1.27-2.39)<br>p=0.001	N/A	Multivariate:<br>GPS, Local Inflammatory Cell Infiltrate, TNM, Paterson Index
26.	Qayyum et al[73]<br>2012	Prospective	Renal Cell	United Kingdom	79	GPS (0/1/2)	No mention of adjuvant therapies	19	N/A	Multivariate:<br>8.64 (3.5–21.29)<br>p<0.001	N/A	Multivariate:<br>Nil else
27.	Suigimoto et al[74]<br>2012	Retrospective	Colorectal	Japan	366	GPS (0/1/2)	Adjuvant chemotherapy administered	67	N/A	Multivariate:<br>3.09 (1.65-5.79)<br>p=0.0004	N/A	Multivariate:<br>Invasion Depth, Lymphatic Invasion, Lymph node metastasis
28.	Kubota et al[75]<br>2012	Retrospective	Gastric	Japan	1017	GPS (0/1/2)	No mention of adjuvant treatment	66	92	Multivariate:<br>GPS 1: 1.26 (0.54-2.56) p=0.5702<br><br>GPS 2: 5.07 (1.94-11.41) p=0.0018	Multivariate:<br>GPS 1: 1.82 (1.00-3.11)<br>p=0.0499<br><br>GPS 2: 5.23 (2.30-10.37)<br>p=0.0003	Multivariate :<br>Age≥75, Upper zone tumour, Lymph node mets, Surgical complications
29.	Powell et al[76]<br>2012	Prospective	Colorectal	United Kingdom	411	mGPS (0/1/2)	Adjuvant therapy offered but no specific information on numbers given	114	191	Multivariate:<br>1.36 (1.03-1.79)<br>p=0.028	N/A	Multivariate:<br>Age, Lymph Node Ratio, Peterson Index, Klintrup score
30.	La Torre et al[77]<br>2012	Retrospective	Pancreatic	Italy	101	GPS (0/1/2)	26 underwent adjuvant treatment including chemotherapy and radiotherapy	N/A	84	N/A	Multivariate:<br>1.7745 (1.1869-2.6532)<br>p=0.005428	Multivariate:<br>LNR, Node status, Margin status

31.	Dutta et al[78] 2012	Retrospective	Gastric	United Kingdom	120	GPS (0/1/2)	Patients received both adjuvant and neoadjuvant therapy specific figures not given	44	51	Multivariate: 2.23 (1.40-3.54) p=0.001	N/A	Multivariate: Elevated lymph node ratio
32.	Wang et al[79] 2012	Retrospective	Gastric	China	324	GPS (0/1/2)	210 patients had adjuvant chemotherapy	N/A	162	N/A	Multivariate: 1.397 (1.070-1.824) p=0.014	Multivariate: The 7th TNM stage, Adjuvant chemotherapy
33.	Lamb et al[80] 2012	Retrospective	Renal	United Kingdom	169	GPS (0/1/2)	No mention of adjuvant therapies	35	59	Multivariate: 6.65 (3.71 – 11.93) p<0.001	Multivariate: 4.17 (2.48 – 7.03) p<0.001	Multivariate: Fuhmann grade, Necrosis, UISS, Leibovich, SSIGN,
34.	Ishizuka et al[81] 2012	Retrospective	Colorectal	Japan	271	GPS (0/1/2)	Adjuvant chemotherapy in 76 cases	42	59	Univariate: OR: 1.986 (1.028-3.840) p=0.041	Multivariate: OR: 2.023 (1.046-3.915) p=0.036	Multivariate: Platelet Count
35.	Jiang et al[82] 2012	Retrospective	Gastric	Japan	1710	mGPS (0/1/2)	No mention of adjuvant treatment	N/A	562	N/A	Multivariate: OR: 1.845 (1.184-2.875) p=0.007	Multivariate: Age, Tumour stage
36.	Jamieson et al[83] 2012	Retrospective	Pancreatic Ductal Adenocarcinoma	UK	173	mGPS (0/1/2)	67 patients received adjuvant chemotherapy	N/A	173	N/A	Multivariate: 1.77 (1.19-2.62) p=0.005	Multivariate: Tumour stage, resection margin status, venous invasion, inflammatory cell infiltrate, adjuvant therapy
37.	Stoz et al[84] 2013	Retrospective	Pancreatic Cancer	Austria	110	GPS (0/1/2)	88 Underwent chemotherapy	N/A	110	N/A	Univariate: 1.095 (0.791-1.574) p=0.585	Multivariate: Stage at diagnosis, NLR
38.	Guthrie et al[47] 2013	Retrospective	Colorectal	United Kingdom	206	mGPS (0/1/2)	58 patients had adjuvant chemotherapy	29	41	Multivariate: Pre-Op: 1.97 (1.16–3.34) P<0.05	N/A	Multivariate: Pre-Op NLR
39.	Shiba et al[85] 2013	Retrospective	Carcinoma of the ampulla	Japan	30	GPS (0/1/2)	No specific mention of adjuvant therapy	N/A	25	N/A	Multivariate: 11.364 (1.017-126.9) p=0.048	Multivariate: Lymph node metastasis

			of vater									
40.	Oshiro et al[86] 2013	Retrospective	Cholangiocarcinoma	Japan	62	GPS (0/1/2)	No mention of adjuvant treatment	N/A	46	N/A	Multivariate: 2.787 (1.153-6.735) p=0.022	Multivariate: Nil Else
41.	Horino et al[87] 2013	Retrospective	HCC	Japan	352	GPS (0/1/2)	No mention of adjuvant treatment	N/A	128	N/A	Multivariate: 3.796 (2.050–7.031) p<0.001	Multivariate: Tumour size, Operation time, Vp
42.	Ishizuka et al[88] 2013	Retrospective	Colorectal Stage IV	Japan	108	GPS 2 vs. 0,1	Majority had adjuvant chemotherapy	72	79	N/A	Multivariate: OR: 0.451 (0.271-0.753) p=0.002	Multivariate: Pathology others, Subclass of stage IV
43.	Ishizuka et al[14] 2013	Retrospective	Colorectal	Japan	481	GPS (0/1/2)	Patients with stage IV disease had chemotherapy	120	150	Multivariate: OR: 2.604 (1.242-5.456) p=0.011	N/A	Multivariate: Pathology, LN Mets, CRP, Albumin, CEA, COP-NLR
44.	Son et al[89] 2013	Retrospective	Colon Cancer	Korea	624	mGPS (2 vs. 0-1)	503 patients received chemotherapy	N/A	55 (5 yr survival)	N/A	Multivariate: 2.217 (0.716-6.864) p=0.167	Multivariate: Fibrinogen, stage, CEA
45.	Nozoe et al[90] 2014	Retrospective	Colorectal	Japan	272	GPS (0/1/2)	No mention of adjuvant treatment	N/A	49	N/A	Multivariate: OR: 7.41 (3.66-15.2) p<0.0001	Multivariate: Tumour stage, venous invasion
46.	Takeno et al[42] 2014	Retrospective	Gastric	Japan	552	mGPS (0/1/2)	No mention of adjuvant treatment	N/A	215	N/A	Multivariate: 1.2391 (0.9188-1.6787) p=0.1598	Multivariate: HS-mGPS
47.	Pinato et al[91] 2014	Retrospective	Lung	United Kingdom	220	GPS (0/1/2)	Adjuvant radio and chemotherapy administered	N/A	61	N/A	Univariate: 1.5 (1.0–2.0) p=0.02	Multivariate: NLR, Pleural Effusion
48.	Huang et al[92] 2014	Prospective	HCC	China	349	GPS (0/1/2)	No mention of adjuvant treatment	N/A	153	N/A	Multivariate: 1.633 (1.226–2.174) p=0.001	Multivariate: CLIP score, BCLC stage

49.	Feng et al[93]<br><br>2014	Retrospective	Oesopha geal	China	493	GPS (0/1/2)	Adjuvant chemo and radiotherapy administered	409 (1 year)	N/A	Univariate:<br>1.907 (1.608-2.262) p<0.001	N/A	Univariate:<br>Tumour depth, Differentiation, Nodal Mets
50.	Forrest et al[94]<br><br>2014	Retrospective	Colorect al	United Kingdom	134	GPS (0/1/2)	No mention of Adjuvant treatment	43	81	Univariate:<br>2.12 (1.41-3.20) p<0.001	N/A	Univariate:<br>T-stage, N-stage, TNM stage, Venous invasion, Peritoneal involvement, Margin involvement, Manual and Automatic Klintrup–Makinen grade
51.	Wu et al[95]<br><br>2014	Retrospective	Gallblad der	China	85	GPS (0 vs 1/2)	13 patients had post op chemotherapy	N/A	75	N/A	Multivariate:<br>10.877 (2.496-47.398) p=0.001	Multivariate:<br>Tumour Invasion, Lymph node metastasis, Margin status
52.	Hirashima et al[40]<br>2014	Retrospective	Gastric	Japan	294	mGPS (0/1/2)	9 patients had neoadjuvant chemotherapy	N/A	38	N/A	Multivariate:<br><75 Years: (n=195)<br>1.24 (0.41-3.75) p=0.70<br><br>>75 Years: (n=99)<br>2.26 (1.09-4.69) p=0.03	Multivariate:<br>Age, Total Gastrectomy, Peritoneal mets, Stage
53.	Nakamura et al[38]<br>2014	Retrospective	Oesopha geal	Japan	168	mGPS (0/1/2)	13 had neoadjuvant treatment while 62 had adjuvant treatment	N/A	44 (3-year survival)	N/A	Multivariate:<br>2.726 (1.021–7.112) p=0.0449	Multivariate:<br>N3: Lymph node, Residual Tumour
54.	Sun et al[96]<br><br>2014	Retrospective	Colon cancer	China	255	mGPS (0/1/2)	No specific mention of neoadjuvant or adjuvant treatment	N/A	94	N/A	Multivariate:<br>RR 2.968 (2.137-4.122) p=0.000	Multivariate:<br>AFP, CEA,fibrinogen, TNM
55.	Nakagawa et al[97]<br><br>2014	Retrospective	Colorect al Liver Metasta ses	Japan	343	mGPS (0/1/2)	69 patients received neoadjuvant chemotherapy, 216 received adjuvant chemotherapy	86	94	Multivariate:<br>1.595 (1.156-2.201)<br>p=0.004	N/A	Multivariate:<br>CEA (<30/ ≥30 ng/L)
56.	Aurello et al[32]<br><br>2014	Retrospective	Gastric Cancer	Italy	102	mGPS (0/1/2)	68 patients received adjuvant chemotherapy after surgery	62	62	N/A	Multivariate:<br>mGPS 1:<br>1.70 (1.20-3.42)<br>p=0.005	Multivariate:<br>Prognostic index

											mGPS 2:<br>1.91 (1.38-3.18)<br>p=0.008	
57.	Miyazaki et al[98]<br><br>2015	Retrospective	NSCLC	Japan	97	GPS (0/1/2)	No mention of adjuvant treatment	29	44	N/A	Multivariate:<br>2.13 (1.036-4.393)<br>p=0.04	Multivariate:<br>Patient factors, Inflammatory factors, stage factors
58.	Matsuda et al[99]<br><br>2015	Retrospective	Oesophageal Cancer	Japan	199	GPS (0/1/2)	99 patients received neoadjuvant chemotherapy/ chemoradiotherapy	N/A	72	N/A	Multivariate:<br>GPS 1:<br>0.562 (0.229-1.377)<br>p=0.208<br><br>GPS 2:<br>0.969 (0.123-7.668)<br>p=0.976	Multivariate:<br>Clinical stage, fibrinogen and albumin score
59.	Farhan-Alanie et al[100]<br>2015	Retrospective	Oral SCC	United Kingdom	178	GPS (0/1/2)	70 patients had adjuvant therapy	42	56	Multivariate:<br>2.12 (1.49-3.00)<br>p<0.001	Multivariate:<br>1.69 (1.23-2.31) p=0.001	Multivariate:<br>Male and AJCC stage 4
60.	Ferro et al[101]<br>2015	Retrospective	Bladder Cancer	Italy	1037	mGPS (0/1/2)	799 received adjuvant chemotherapy	426	430	Multivariate:<br>mGPS 1: 0.87 (0.54-1.40)<br>p=0.565<br>mGPS 2: 0.94 (0.49-1.81<br>p=0.853	Multivariate:<br>mGPS 1: 1.19 (0.84-1.70)<br>p=0.332<br>mGPS 2: 1.25 (0.74-2.11)<br>p=0.410	Multivariate:<br><br>Pathologic stage T4, Node positive and adjuvant Chemotherapy
61.	Arigami et al[39]<br>2015	Retrospective	Oesophageal Cancer	Japan	238	mGPS (0/1/2)	No mention of adjuvant therapy	N/A	98	N/A	Multivariate:<br>1.08 (0.49-2.19) p=0.830	Multivariate:<br>F-NLR Score, Lymph node mets, Depth of tumour invasion
62.	Xu et al[22]<br>2015	Retrospective	Oesophageal SCC	China	468	GPS/mGPS (0/1/2)	196 patient received adjuvant chemo and radiotherapy	N/A	259	N/A	Univariate:<br>GPS 1: 1.33 (0.99-1.78)<br>p=0.057<br>GPS 2: 1.83 (1.18-2.86)<br>p=0.008<br><br>mGPS 1: 1.39 (1.01-1.91)<br>p=0.046<br>mGPS 2: 1.82 (1.17-2.83)<br>p=0.008	Multivariate:<br>Lymph Node Mets, Venous/lymphatic invasion, CRP/Alb Ratio

63.	Ni[102] 2015	Retrospective	HCC	China	367	mGPS (0/1/2)	No mention of adjuvant treatment	N/A	40	N/A	Multivariate: 4.356 (2.495-7.605) p<0.001	Multivariate: GGT≥60, AFP≥400, CLIP Score, Vascular Invasion
64.	Hirahara[103] 2015	Retrospective	Oesophageal	Japan	141	GPS (0/1/2)	No mention of adjuvant treatment	N/A	16	N/A	Multivariate: 2.045 (1.032-3.928) p=0.041	Multivariate: p Stage
65.	Shibutani[104] 2015	Retrospective	Colorectal	Japan	254	GPS (0/1/2)	Adjuvant chemotherapy	N/A	69	N/A	Multivariate: 7.238 (1.180-44.415) p=0.032	Multivariate: NLR (Pre & Post op), Number of lymph node mets
66.	Shiba et al[105] 2015	Retrospective	Gallbladder Ca	Japan	51	GPS (0/1/2)	No mention of adjuvant treatment	N/A	16	N/A	Multivariate: 3.782 (1.119-12.786) p=0.032	Multivariate: Lymph node metastasis
67.	Kawashima et al[41] 2015	Retrospective	Lung Cancer	Japan	1043	GPS (0/1/2)	No mention of adjuvant treatment	N/A	227	N/A	Multivariate: GPS 1 1.63 (1.09-2.42) p=0.02<br><br>GPS 2 1.44 (0.80-2.60) p=0.22	Multivariate: Age, smoking, preoperative co-morbidity, CEA, pathological stage, histological tumour type, LVI, surgical procedure
68.	Watt et al[106] 2015	Retrospective	Colorectal cancer	UK	508	mGPS (0/1/2)	108 patients had adjuvant chemotherapy following resection.	172	292	Multivariate: 1.54 (1.25-1.90) p< 0.001	Multivariate: 1.32 (1.12-1.56) p=0.001	Multivariate: Age, site, TNM stage, margin involvement, peritoneal involvement, sex, venous invasion, tumour perforation
69.	Okamura et al[107] 2015	Retrospective	HCC	Japan	256	GPS (0/1/2)	No mention of adjuvant treatment	N/A	86	N/A	Multivariate: 1.71 (0.92-3.16) p=0.089	Multivariate: AFP, des-gamma-carboxy prothrombin, high NLR, low PNI.
70.	Abe et al[108] 2016	Retrospective	HCC	Japan	46	GPS (0/ 1,2)	No mention of adjuvant treatment	N/A	17	N/A	Multivariate: 7.718 (1.710-34.840) p=0.008	Multivariate: Milan criteria

71.	Ishizuka et al[21] 2016	Retrospective	Colorectal Cancer	Japan	627	GPS (2/0, 1)	No mention of adjuvant treatment	110	142	N/A	Multivariate: 1.809 (1.181-2.772) p=0.006	Multivariate: Pathological differentiation, CEA, stage, CAR, NLR
72.	Park[109] 2016	Retrospective	Colorectal Cancer	United Kingdom	228	GPS (0/1/2)	131 received adjuvant therapy	66	N/A	Multivariate: 1.59 (1.12–2.27) p=0.010	N/A	Multivariate: CD3 cancer cells nest density (low/high), NPS
73.	Park[7] 2016	Retrospective	Colorectal	United Kingdom	1000	mGPS (0/1/2)	Adjuvant therapy: 248<br>Neoadjuvant therapy: 98	242	435	Multivariate: 1.28 (1.09-1.52) p=0.003	Multivariate: 1.28 (1.13-1.45) p<0.001	Multivariate: Age, Adjuvant therapy, T stage, N stage, Differentiation, Margins involved
74.	Fu et al[110] 2016	Retrospective	HCC	China	Training: 772	GPS (0/1/2)<br><br>mGPS (0/1/2)	No mention of adjuvant treatment	N/A	377 (4-year survival)	N/A	Multivariate:<br><br>Training cohort:<br><br>mGPS 3.508 (1.384-8.890) p=0.008	Multivariate:<br><br>AFP, GGT, IBS, PLR, PI, tumour size, tumour number, microscopic vascular invasion, differentiation, BCLC.
75.	Fan et al[111] 2016	Retrospective	Non-small Cell Lung Cancer	China	1243	GPS (0/1/2)<br>mGPS (0/1/2)	684 patients received chemotherapy, 220 patients received radiotherapy	N/A	373	N/A	Multivariate:<br>GPS:<br>2.228 (1.447-3.431) p< 0.0001<br><br>mGPS:<br>0.958 (0.633-1.452) p=0.841	Multivariate: Gender, age, TNM stage, chemotherapy, radiotherapy
76.	Chan et al[112]<br><br>2016	Retrospective	Colorectal Cancer	Australia	386	mGPS (0/1/2)	Patients with high-risk stage II and III colon cancer disease were generally offered standard adjuvant chemotherapy, whereas those with stage II or III rectal	N/A	353	N/A	Univariate:<br>mGPS 1:<br>1.552 (0.892-2.700) P=0.001<br><br>mGPS 2:<br>2.214 (1.454-3.369) p=0.001	Multivariate: Age, T stage, grade, LMR

							cancers were usually treated with neoadjuvant					
77.	Walsh et al[113] 2016	Retrospective	Esophageal Cancer	Ireland	223	mGPS (0 vs. 1/2)	109 patients received neoadjuvant chemoradiotherapy, 66 patients received chemotherapy	N/A	104 (5-year survival)	N/A	Multivariate:<br>1.24 (0.69-2.22)<br>p=0.47	Multivariate:<br>TNM stage, nodal status
78.	Otowa et al[114]<br><br>2016	Retrospective	Oesophageal Cancer	Japan	100	Pre-NAC mGPS (0/1-2)<br><br>Post-NAC mGPS (0/2)<br><br>NAC=neoadjuvant chemotherapy	All patients underwent NAC followed by surgery	N/A	36	N/A	Mul;tivariatee:<br>Pre-NAC mGPS:<br>0.043 (0.001–1.311)<br>p=0.067<br><br>Post-NAC mGPS:<br>0.020 (0.018–0.621)<br>p=0.018	Multivariate:<br>Grade of response to chemotherapy
79.	Melling et al[115]<br><br>2016	Retrospective	Gastric Cancer	Germany	88	GPS (0/1/2)	Any neoadjuvant/adjuvant therapy was an exclusion criteria	N/A	57	N/A	Multivariate:<br>OR 1.6 (1.0-2.4)<br>p=0.033	Multivariate:<br>Nil else
80.	Toyokawa et al[37]<br><br>2016	Retrospective	Thoracic Oesophageal Squamous Cell Carcinoma	Japan	185	GPS (0 vs 1/2)	46 patients received neoadjuvant treatment (39 chemotherapy, 6 chemoradiotherapy, 1 radiotherapy)	N/A	77	N/A	Multivariate:<br>1.021 (0.465-2.245)<br>p=0.958	Multivariate:<br>Sex, performance status, ASA, cTNM stage, CONUT score

Table 2: Studies investigating the prognostic value of NLR in an unselected cohort of patients with operable cancer (Abbreviations: Red blood cell count, WBC: White blood cell count, Th (T-helper cells), D2-40 (Chalkley score), pT (T-stage), pN (N-stage), NSAIDS (Non-steroidal inflammatory drugs), CNP (combined NLR and PLR ratio), ER (estrogens receptor), FAP-α (Fibroblast activation protein-α), PI (Prognostic index), KPS (Karnofsky Performance Score), MGMT (O^6-methylguanine-DNA-methyltransferase), HR (herceptin receptor), FIGO (Federation of Gynecology and Obstetrics), LVSI (Lymphovascular space invasion), Red cell distribution (RDW), ECOG-PS (ECOG-performance status))

**No: NLR**	**Study**	**Type of Study**	**Cancer**	**Country**	**Patients (n)**	**Measure of SIR**	**Additional Treatment**	**Cancer deaths (n)**	**Overall deaths (n)**	**Cancer survival (HR, 95%CI)**	**Overall survival (HR, 95%CI)**	**Independent Prognostic Factors**
1.	Halazun et al[116] 2008	Retrospective	Colorectal Liver Metastases	United Kingdom	440	NLR >5	Adjuvant therapy of 5-FU/folinic acid	N/A	395 (5-year survival)	N/A	Multivariate: 2.275 (1.654-3.129) p<0.0001	Multivariate: Age, tumour number
2.	Gomez et al[117] 2008	Retrospective	Intrahepatic cholangiocarcinoma	United Kingdom	27	NLR ≥5	No mention of adjuvant or neoadjuvant treatment	N/A	21	N/A	Multivariate: RR: 1.778 (0.558-5.668) p=0.331	Multivariate: Nil else
3.	Sarraf et al[118] 2009	Retrospective	Non Small Cell Lung Cancer	United Kingdom	177	NLR (tertiles)	No mention of adjuvant or neoadjuvant treatment	N/A	81 (5-year survival)	N/A	Multivariate: 1.10 (1.03-1.17) p= 0.005	Multivariate: Stage of disease
4.	Kishi et al[119] 2009	Retrospective	Colorectal Liver Metastases	US	200	NLR >5	Neoadjuvant chemotherapy	N/A	118 (5-year survival)	N/A	Multivariate: 2.0 (1.0-3.8) p= 0.048	Multivariate: Postoperative factors namely concomitant radiofrequency ablation (RFA) and surgical margin
5.	Cho et al[120] 2009	Retrospective	Epithelial Ovarian Cancer	South Korea	192	NLR >2.6	Adjuvant chemotherapy	N/A	20	N/A	Multivariate: 8.42 (1.09-64.84) p=0.041	Multivariate: Age, stage
6.	Smith et al[121] 2009	Retrospective	Pancreatic Ductal Adenocarcinoma	UK	110	NLR continuous	33 patients had adjuvant therapy	N/A	106	N/A	Univariate: 1.047 (0.985-1.113) p=0.14	Multivariate: Lymphocyte count, PLR
7.	Halazun et al[122] 2009	Retrospective	HCC	US	150	NLR ≥5	116 patients received pretransplant tumour therapy	N/A	61	N/A	Multivariate: 6.102 (2.286-16.290) p<0.0001	Multivariate: Preoperative AFP

8.	Jagdev et al[123]<br><br>2010	Retrospective	Renal Cell Carcinoma	UK	286	Log (NLR)	No mention of adjuvant or neoadjuvant treatment	63 (5-year survival)	111 (5-year survival)	Multivariate:<br>4.2 (1.6-11)<br>p=0.004	Univariate:<br>2.1 (1.5-2.8)<br>p<0.001	Multivariate:<br>Log CRP, stage, grade, RBC, WBC, M stage, necrosis, micro vascular invasion
9.	Ubukata et al[23]<br><br>2010	Retrospective	Gastric Cancer	Japan	157	NLR ≥5	No neoadjuvant therapy.	N/A	77	N/A	Multivariate:<br>RR: 5.779 (0.950-35.170)<br>p=0.0001	Multivariate:<br>Th1/ Th2 ratio, pathological stage, depth of invasion, tumour size, lymph node metastasis
10.	Shimada et al[124]<br><br>2010	Retrospective	Gastric Cancer	Japan	1028	NLR ≥4	No mention of adjuvant or neoadjuvant treatment	128	147	N/A	Multivariate:<br>1.845 (1.236-2.747)<br>p=0.003	Multivariate:<br>Tumour depth, N factor, distant/ peritoneal metastasis, histology, platelet count
11.	Bhatti et al[125]<br><br>2010	Retrospective	Pancreatic ductal adenocarci noma	UK	84	NLR (continuous)	30 patients received adjuvant chemotherapy	N/A	66 (3-year survival)	N/A	Multivariate:<br>1.210 (1.010-1.449)<br>p=0.039	Multivariate:<br>Lymphocyte count, resection margin
12.	Mohri et al[126]<br><br>2010	Retrospective	Gastric Cancer	Japan	357	NLR >2.2	No neoadjuvant therapy	N/A	98	N/A	Multivariate:<br>2.78 (1.79-4.36)<br>p<0.0001	Multivariate:<br>Tumour size, clinical T stage
13.	Liu et al[127]<br><br>2010	Retrospective	Rectal carcinoma	China	123	NLR >2	Stage II cancers received adjuvant chemotherapy	N/A	123	N/A	Multivariate:<br>2.615 (1.152-5.933)<br>p=0.021	Multivariate:<br>Depth of invasion, tumour size, CA12-5 level, stage
14.	Miyata et al[24]<br><br>2011	Retrospective	Oesophage al Cancer	Japan	152	NLR ≥4	All patients received neoadjuvant chemotherapy	N.A	92 (5-year survival)	N/A	Multivariate:<br>1.30 (0.76-2.22)<br>p=0.3362	Multivariate:<br>Clinical response, SI score, number of metastatic lymph nodes, operative complication
15.	Dutta et al[62]<br>2011	Retrospective	Oesophagu s	United Kingdom	112	NLR (<2.5/ 2.5-5/ >5)	31 had neoadjuvant and 14 adjuvant therapy	52	59	Univariate:<br>1.08 (0.75-1.56)<br>p=0.686	N/A	Multivariate:<br>Positive to total lymph node ratio (0/≤0.2/>0.2), mGPS

16.	Kao et al[128]<br><br>2011	Retrospective	Malignant pleural mesothelio ma	Australia	85	NLR ≥3	19 patients received neoadjuvant chemotherapy	N/A	72 (5-year survival)	N/A	Multivariate:<br>1.79 (1.04-3.07)<br>p=0.04	Multivariate:<br>Gender, histological subtype, calretinin score, D2-40 score
17.	Jung et al[129]<br><br>2011	Retrospective	Gastric cancer	Korea	293	NLR ≥2	183 patients received adjuvant chemotherapy	N/A	166	N/A	Multivariate:<br>1.462 (1.033-2.068)<br>p=0.032	Multivariate:<br>Combined resection radicalism, Lauren classification, postoperative chemotherapy
18.	Sharaiha et al[130]<br><br>2011	Retrospective	Esophageal cancer	US	295	NLR ≥5	127 received neoadjuvant therapy (chemo/ radiotherapy)	N/A	160 (5-year survival)	N/A	Multivariate:<br>2.32 (1.53-3.50)<br>p<0.0001	Multivariate:<br>Age, sex, stage, tumour differentiation, comorbidities
19.	Tomita et al[131]<br><br>2011	Retrospective	Non-small Cell Lung Cancer	Japan	284	NLR≥2.5	No mention of adjuvant or neoadjuvant treatment	N/A	109 (5-year survival)	N/A	Multivariate:<br>RR: 1.2863 (1.0462-1.5738)<br>p=0.0173	Multivariate:<br>Age, histology, pT, pN, pleural lavage cytology
20.	Hung et al[132]<br><br>2011	Retrospective	Colon cancer	Taiwan	1040	NLR ≥5	No neoadjuvant therapy administered	122	334	N/A	Multivariate:<br>1.29 (1.07-1.80)<br>p=0.012	Multivariate:<br>Age, CEA, examined lymph node no. <12, T stage, tumour obstruction/ perforation
21.	Neal et al[133]<br><br>2011	Retrospective	Colorectal Liver Metastases	UK	202	NLR ≥5	84 patients had systemic chemotherapy in the 6 months before liver resection	N/A	127 (5-year survival)	N/A	Univariate:<br>2.51 (1.56-4.02)<br>p<0.001	Multivariate:<br>Clinical risk score, neutrophil count, serum albumin
22.	Asher et al[134]<br><br>2011	Retrospective	Ovarian Cancer	UK	235	NLR>4	170 patients received chemotherapy	N/A	169 (survival after 150 months)	N/A	Multivariate:<br>0.865 (0.521-1.437)<br>p=0.575	Multivariate:<br>Age, stage, residual disease, PLR
23.	Wang et al[135]<br><br>2011	Retrospective	HCC	China	101	NLR≥3	35 patients received pre-transplant tumour therapy	N/A	51	N/A	Multivariate:<br>2.654 (1.419-4.964)<br>p<0.001	Multivariate:<br>Tumour numbers, vascular invasion
24.	Bertuzzo et al[136]<br><br>2011	Retrospective	HCC	Italy	219	NLR ≥5	159 patients received neoadjuvant treatments (TACE,	27	61	N/A	Multivariate:<br>OR: 4.868 (2.473-9.582)<br>p< 0.0001	Multivariate:<br>Microvascular invasion

							PEI, RFA)					
25.	Idowu et al[137]<br><br>2012	Retrospective	Soft Tissue Sarcoma	UK	223	NLR ≥5	No mention of adjuvant or neoadjuvant treatment	N/A	44 (5-year survival)	N/A	Multivariate:<br>5.125 (1.245-21.086)<br>p=0.024	Multivariate:<br>Grade, surgical margin.
26.	Ishizuka et al[138]<br><br>2012	Retrospective	Colorectal Cancer	Japan	169	NLR (continuous)	Adjuvant chemotherapy in most patients	86	96	N/A	Multivariate:<br>OR: 0.980 (0.870-1.106)<br>p=0.747	Multivariate:<br>Tumour pathology
27.	Wang et al[79]<br>2012	Retrospective	Gastric	China	324	NLR >5	210 patients had adjuvant chemotherapy	N/A	162	N/A	Multivariate:<br>1.866 (0.901-3.866)<br>p=0.093	Multivariate:<br>The 7th TNM stage, adjuvant chemotherapy
28.	Gondo et al[139]<br><br>2012	Retrospective	Bladder cancer	Japan	189	NLR ≥2.5	38 received intravesical chemotherapy	54	N/A	Multivariate:<br>1.946 (1.035-3.663)<br>p=0.0387	N/A	Multivariate:<br>Tumour size, Hb
29.	Kwon et al[140]<br><br>2012	Retrospective	Colorectal cancer	Korea	200	NLR ≥5	150 patients received adjuvant chemotherapy/ chemoradiation	N/A	39	N/A	Multivariate:<br>1.520 (0.613-3.772)<br>p=0.367	Multivariate:<br>Stage, CEA,PLR
30.	Carruthers et al[141]<br><br>2012	Retrospective	Rectal cancer	UK	115	NLR ≥5	Neoadjuvant chemoradiation	N/A	43	N/A	Multivariate:<br>7.0 (2.6-19.2)<br>p<0.001	Multivariate: Total WBC, platelet count, R status, down staging
31.	Dutta et al[78]<br>2012	Retrospective	Gastric	United Kingdom	120	NLR (<2.5/ 2.5-5/ >5)	Patients received both adjuvant and neoadjuvant therapy specific figures not given	44	51	Univariate:<br>1.19 (0.76-1.87)<br>p=0.454	N/A	Multivariate:<br>Positive lymph node ratio
32.	Wang et al[142]<br><br>2013	Retrospective	Oesophageal Cacinosarcoma	China	33	NLR≥5	4 patients received adjuvant chemotherapy, 3 received adjuvant radiotherapy	N/A	14	N/A	Multivariate:<br>138.47 (6.772-2831.214)<br>p=0.001	Multivariate:<br>Nil else
33.	Choi et al[143]<br><br>2013	Retrospective	Soft Tissue Sarcoma	Korea	162	NLR >2.5	7 patients received neoadjuvant chemotherapy, 72 patients received adjuvant radiation, 36 patients received	20	20	Multivariate:<br>OR: 1.32 (0.55-3.21) p=0.096	N/A	Multivariate:<br>CRP, ESR, number of elevated markers

							adjuvant chemotherapy				
34.	Szkandera et al[144]<br><br>2013	Retrospective	Soft Tissue Sarcoma	Austria	260	NLR <3.58vs. ≥3.58	167 patients received adjuvant radiotherapy, 35 received adjuvant chemotherapy	N/A	86	N/A	Multivariate:<br>1.88 (1.14-3.12)<br>P=0.014	Multivariate:<br>Sex, tumour necrosis, tumour stage
35.	Krane et al[145]<br><br>2013	Retrospective	Bladder Cancer	US	68	NLR >2.5	10 patients received neoadjuvant chemotherapy	25	40	Multivariate:<br>RR 2.68 (1.01-8.59)	Multivariate:<br>RR 2.49 (1.14-6.09)	Multivariate:<br>Hypoalbuminaemia, pT3, nodal disease.
36.	Pichler et al[146]<br><br>2013	Retrospective	Renal Cell Carcinoma	Austria	678	NLR <3.3vs. ≥3.3	No mention of adjuvant or neoadjuvant treatment	59	123	Multivariate:<br>1.59 (0.84-2.99)<br>P=0.148	Multivariate:<br>1.59 (1.10-2.31)<br>P=0.014	Multivariate:<br>Age, T stage, tumour grade, presence of tumour necrosis
37.	Jankova et al[147]<br><br>2013	Retrospective	Colorectal cancer	Australia	322	NLR (continuous)	7 patients received adjuvant radiotherapy, 197 received adjuvant chemotherapy	86	141	Multivariate:<br>1.01 (0.92-1.12)<br>P=0.782	Multivariate:<br>1.06 (1.01-1.12)<br>P=0.013	Multivariate:<br>Age, direct spread beyond muscularis propria, nodes involvement, adjacent structure infiltrated, postoperative chemotherapy, sex
38.	Fu et al[148]<br><br>2013	Retrospective	Hepatocellular Carcinoma	China	282	NLR>2	No mention of adjuvant or neoadjuvant treatment	N/A	173	N/A	Multivariate:<br>1.434 (1.044-1.970)<br>P=0.026	Multivariate:<br>Tumour size, tumour number, macroscopic vascular invasion, Child-Pugh class
39.	Shibutani et al[149]<br><br>2013	Retrospective	Colorectal Cancer	Japan	674	NLR ≥2.5	No mention of adjuvant or neoadjuvant treatment	136	177	Multivariate:<br>1.609 (1.117-2.319)<br>P=0.011	N/A	Multivariate:<br>Tumour diameter, lymph node metastasis, distant metastasis
40.	Forget et al[150]<br><br>2013	Retrospective	Breast Cancer	Belgium	Centre 1: n=172<br><br>Centre 2: n=162	Centre 1: NLR. ≥4<br><br>Centre 2: NLR. ≥3	No mention of adjuvant or neoadjuvant treatment	N/A	Centre 1: 17 (at 60 months)<br><br>Centre 2: 8 (at 24 months)	N/A	Centre 1:<br>Univariate<br>0.51 (0.35-8.58)<br>P=0.47	Univariate:<br>Ketorolac or diclofenac use

											Centre 2:<br>Univariate<br>4.00 (1.12-14.3)<br>P=0.03	
41.	Forget et al[150]<br><br>2013	Retrospective	NSCLC	Belgium	255	NLR≥5	No mention of adjuvant or neoadjuvant treatment	N/A	109 (at 60 months)	N/A	Univariate:<br>1.52 (1.07-2.17) P=0.02	Multivariate:<br>Pneumonectomy, Ketorolac (vs. no NSAIDS)
42.	Forget et al[150]<br><br>2013	Retrospective	Kidney Cancer	Belgium	227	NLR≥5	No mention of adjuvant or neoadjuvant treatment	N/A	64 (at 60 months)	N/A	Multivariate:<br>1.67 (1.0-2.81) p=0.05	Multivariate:<br>Node status, stage, histological stage
43.	Absenger et al[151]<br><br>2013	Retrospective	Colon Cancer	Austria	372	dNLR (≤2.2 vs. >2.2)<br><br>preoperative NLR >4<br><br>preoperative NLR ≥5	230 patients received adjuvant chemotherapy	N/A	72	N/A	Multivariate:<br>dNLR<br>1.78 (1.07-2.97) p=0.026<br><br>Preoperative NLR >4<br>2.22 (1.36-3.62) p=0.002<br><br>Preoperative NLR ≥5<br>1.68 (1.03-2.73) p=0.037	Multivariate:<br>Clinical stage
44.	Feng et al[18]<br><br>2013	Retrospective	Oesophageal Squamous Cell Carcinoma	China	483	NLR >3.45	No mention of adjuvant or neoadjuvant treatment	N/A	244	N/A	Multivariate:<br>1.310 (0.997-1.722) p=0.053	Multivariate:<br>Differentiation, depth of invasion, node metastasis, PLR, CNP
45.	Mano et al[152]<br><br>2013	Retrospective	Hepatocellular Carcinoma	Japan	958	NLR ≥2.81	No mention of adjuvant or neoadjuvant treatment	N/A	310 (5-year survival)	N/A	Multivariate:<br>3.745 (1.027-1.088) p=0.0002	Multivariate:<br>Albumin, tumour size, portal vein thrombus, stage, multiple tumours
46.	Azuma et al[153]<br><br>2013	Retrospective	Upper Urinary Tract Urothelial Carcinoma	Japan	137	NLR ≥2.5	No mention of adjuvant or neoadjuvant treatment	54 (5-year survival)	N/A	Multivariate:<br>3.06 (1.44-6.83) p=0.0035	N/A	Multivariate:<br>pTstage, lymphovascular invasion
47.	Dumitrascu et al[154]<br><br>2013	Retrospective	Hilar Cholangiocarcinoma	Romania	90	NLR <3.3	43 received adjuvant treatment (chemotherapy, radiotherapy or chemoradiotherapy)	51	56	N/A	Multivariate:<br>RR 0.76 (0.57-1) p=0.053	Multivariate:<br>Adjuvant chemotherapy with gemcitabine, R0 resection, caudate lobe invasion

48.	Perisanidis et al[155]<br><br>2013	Retrospective	Oral Cancer	Austria	97	NLR >1.9	All patients treated with neoadjuvant chemoradiotherapy	17	35	Multivariate:<br>10.37 (1.28-84.08)<br>p=0.029	N/A	Multivariate:<br>ypTNM, perineural invasion
49.	Noh et al[156]<br><br>2013	Retrospective	Breast Cancer	Korea	442	NLR ≥2.5	Triple negative cancers are treated with chemotherapy	25 (5-year survival)	32	Multivariate:<br>4.08 (1.62-10.28)<br>p=0,003	N/A	Multivariate:<br>Node status, ER status
50.	Liao et al[157]<br><br>2013	Retrospective	Non-small Cell Lung Cancer	China	59	NLR continuous	Patients who underwent neoadjuvant chemotherapy and/or radiotherapy were excluded	N/A	23 (after 40 months)	N/A	Multivariate:<br>1.00 (0.40-2.49)<br>p=0.98	Multivariate:<br>Tumour differentiation, FAP-α percentage/ grade.
51.	Bambury et al[158]<br><br>2013	Retrospective	Glioblastom a multiforme	Ireland	84	NLR >4	49 patients received complete Stupp protocol (using concurrent chemoradiotherapy followed by consolidation chemotherapy with temozolomide)	N/A	82	N/A	Multivariate:<br>1.81 (1.08-3.01)<br>p=0.025	Multivariate:<br>Age, gender, extent of resection, full Stupp protocol
52.	Toiyama et al[159]<br><br>2013	Retrospective	Rectal Cancer	Japan	84	NLR >3	All patients received neoadjuvant chemoradiotherapy	N/A	37 (after 150 months)	N/A	Multivariate:<br>0.98 (0.37-2.56)<br>p=0.96	Multivariate:<br>Pathological TNM stage, CRP
53.	Son et al[89]<br><br>2013	Retrospective	Colon Cancer	Korea	624	NLR ≥5	503 patients received chemotherapy	N/A	55 (5 yr survival)	N/A	Multivariate:<br>1.841 (0.470-7.204)<br>p=0.381	Multivariate:<br>Fibrinogen, stage, CEA
54.	Stoz et al [84]<br><br>2013	Retrospective	Pancreatic Cancer	Austria	110	NLR≥5	88 Underwent chemotherapy	N/A	110	Multivariate:<br>1.611 (1.024-2.534) p=0.039	N/A	Multivariate:<br>Stage at diagnosis, NLR
55.	Guthrie et al[47]<br>2013	Retrospective	Colorectal	United Kingdom	206	NLR>5	58 patients had adjuvant chemotherapy	29	41	Multivariate<br>Pre-Op:<br>3.07 (1.23–7.63)<br>P<0.05	N/A	Multivariate:<br>Pre-Op and Post-Op mGPS
56.	Ishizuka et al[14]<br>2013	Retrospective	Colorectal	Japan	481	NLR>3	Patients with stage IV disease had chemotherapy	120	150	Univariate:<br>OR: 0.961 (0.843-1.096) p=0.554	N/A	Multivariate:<br>Pathology, LN Mets, CRP, Albumin, CEA, GPS

57.	Szkandera et al[160]<br><br>2014	Retrospective	Soft Tissue Sarcoma	Austria	340 Training set, n=170<br><br>Validation set, n=170	NLR ≥5	Training set:<br>16 received adjuvant chemotherapy, 102 received adjuvant radiotherapy<br><br>Validation set:<br>22 received adjuvant chemotherapy, 107 received adjuvant radiotherapy	Training set: 30<br>Validation set: 22	Training set: 53<br>Validation set: 51	Univariate:<br>Training set:<br>2.14 (0.81-5.66) p=0.124<br><br>Validation set:<br>1.98 (0.77-5.08) p=0.153	Multivariate:<br>Training set:<br>1.68 (0.75-3.76) p=0.201<br><br>Validation set:<br>2.84 (1.37-5.87) p=0.005	Multivariate:<br>Age, tumour grade, LMR, tumour size
58.	Dalpiaz et al[161]<br><br>2014	Retrospective	Upper Tract Urothelial Carcinoma	Austria	202	NLR ≥2.7	No mention of adjuvant or neoadjuvant treatment	58	147	Multivariate:<br>2.718 (1.246-5.928)<br>P=0.012	Multivariate:<br>2.480 (1.308-4.702)<br>P=0.005	Multivariate:<br>pT stage
59.	Luo et al[162]<br><br>2014	Retrospective	Upper Urinary Tract Urothelial Carcinoma	Taiwan	234	NLR >3	Patients underwent RNU without neoadjuvant or adjuvant intervention.	24	N/A	Multivariate:<br>6.38 (1.75-23.31)<br>p=0.006	N/A	Multivariate:<br>Pathological stage, age, smoking
60.	Wu et al[95]<br><br>2014	Retrospective	Gallbladder	China	85	NLR >2.3	13 patients had post op chemotherapy	N/A	75	N/A	Univariate:<br>1.769 (1.111-2.818)<br>p=0.016	Multivariate:<br>Tumour Invasion, Lymph node metastasis, Margin status
61.	Zhang et al[163]<br><br>2014	Retrospective	Non-Small Cell Lung Cancer	China	400	NLR <3.3vs. ≥3.3	Patients treated with neoadjuvant and adjuvant therapy were excluded	86	N/A	N/A	Multivariate:<br>2.075 (1.317-3.271)<br>p=0.002	Multivariate:<br>Age, tumour size
62.	Ying et al[164]<br><br>2014	Retrospective	Colorectal Cancer	China	205	NLR≥3.12	77 colon and 31 rectal cancer patients underwent chemotherapy	100	112	Multivariate:<br>2.77 (1.72-4.46)<br>p<0.001	Multivariate:<br>2.73 (1.74-4.29)<br>p<0.001	Multivariate:<br>Grade (G3/G4), chemotherapy
63.	Linton et al[165]<br><br>2014	Retrospective	Malignant Pleural Mesothelioma	Australia	59	NLR. ≥5	64% received adjuvant radiotherapy, 33% received induction or adjuvant chemotherapy	N/A	24 (survival >20 months)	N/A	Survival after 4 months<br>Univariate:<br><br>NLR≥5 0.86 (0.40-1.82)<br>p=0.69	Multivariate:<br>Nil else

64.	Ishizuka et al[15]<br><br>2014	Retrospective	Gastric Cancer	Japan	544	NLR (≤3 vs. >3)	343 patients received adjuvant chemotherapy	55	108	N/A	Univariate:<br>1.990 (1.417-2.793)<br>p<0.001	Multivariate:<br>Age, tumour type, lymph node metastasis, albumin, COP-NLR
65.	Kubo et al[166]<br><br>2014	Retrospective	Colorectal carcinoma	Japan	524	NLR (high/low)	Adjuvant chemotherapy in 156 patients with stage 3 cancer and 38 patients with stage 2 cancer	74	104	Multivariate:<br>1.71 (1.03-2.88)<br>p=0.04	N/A	Multivariate:<br>Cancer site, T stage, lymph node metastasis
66.	Viers et al[167]<br><br>2014	Retrospective	Clear Cell Renal Carcinoma	US	827	NLR (continuous)	No mention of adjuvant or neoadjuvant treatment	233	436	Multivariate:<br>1.02 (1.01-1.04)<br>p=0.009	Multivariate:<br>1.02 (1.01-1.03)<br>p=0.004	Multivariate:<br>ECOG performance status, tumour size, constitutional symptoms, age
67.	Koh et al[168]<br><br>2014	Retrospective	Breast Cancer	South Korea	157	NLR >2.25)	All treated with neoadjuvant chemotherapy	N/A	25	N/A	Multivariate:<br>24.87 (3.075-201.3)<br>p=0.003	Multivariate:<br>Nil else
68.	Hermanns et al[169]<br><br>2014	Retrospective	Bladder cancer	Canada	424	NLR ≥3	29 patients received neo-adjuvant chemotherapy, 87 received adjuvant chemotherapy, 55 received salvage chemotherapy	110	178	Multivariate:<br>1.88 (1.39-2.54)<br>p<0.001	Multivariate:<br>1.67 (1.17-2.39)<br>p=0.005	Multivariate:<br>Charlson Comorbidity Index, Hb, platelets, N-stage, year of radical cystectomy, lymphovascular invasion
69.	Tanaka et al[170]<br><br>2014	Retrospective	Upper Tract Urothelial Carcinoma	Japan	665	NLR >3	129 patients received adjuvant chemotherapy	129	N/A	Multivariate:<br>1.47 (1.03-2.11)<br>p=0.036	N/A	Multivariate:<br>Age, pathological T stage, lymphovascular invasion, lymph node involvement
70.	Jiang et al[171]<br><br>2014	Retrospective	Gastric Cancer	China	377	NLR <1.44 vs. ≥1.44	219 patients received adjuvant chemotherapy post gastrectomy	N/A	223	N/A	Multivariate:<br>1.595 (1.045-2.435)<br>p=0.030	Multivariate:<br>Tumour size, serosal invasion, lymph node metastasis, post complication

71.	Yuan et al[172] 2014	Retrospective	Adenocarcinoma of Esophagogastric Junction	China	327	NLR <5 vs. ≥5	18 patients received neoadjuvant chemotherapy, 59 patients received adjuvant chemotherapy	N/A	168	N/A	Multivariate: 2.551 (1.847-3.524) p<0.0001	Multivariate: pTNM stage, adjuvant treatment
72.	Ozdemir et al[173] 2014	Retrospective	Colorectal Cancer	Turkey	281	NLR (≤2.2 vs. >2.2)	Patients with lymph node invasion, vascular invasion, perineural invasion and high neoadjuvant CEA were given adjuvant chemotherapy	N/A	134	N/A	Multivariate: 3.306 (1.713-6.378) p=0.005	Multivariate: pN stage, pTNM stage.
73.	Dalpiaz et al[161] 2014	Retrospective	Upper Tract Urothelial Carcinoma	Austria	171	dNLR (continuous),	No mention of adjuvant or neoadjuvant treatment	54	79	Multivariate: 1.16 (1.01-1.35) p=0.045	Multivariate: 1.21 (1.09-1.34) p<0.001	Multivariate: Age at operation, pT-stage
74.	Feng et al[174] 2014	Retrospective	Esophageal SCC	China	483	NLR ≥3.5	No mention of adjuvant or neoadjuvant treatment	N/A	244	N/A	Multivariate: 1.339 (1.015-1.768) p=0.039	Multivariate: Differentiation, depth of invasion, nodal metastasis, PLR
75.	Viers et al[175] 2014	Retrospective	Bladder Cancer	USA	899	NLR (continuous)	117 patients received adjuvant therapy (radiation or chemotherapy)	345	615	Multivariate: 1.04 (1.01-1.08) p=0.01	Multivariate: 1.03 (1.01-1.06) p=0.01	Multivariate: Age at surgery, ECOG performance status, pathologic tumour stage, lymph node density, lymphovascular invasion
76.	McNamara et al[176] 2014	Retrospective	Biliary Tract Cancer	Canada	326	NLR ≥3	90 received adjuvant chemotherapy	N/A	199	N/A	Multivariate: 1.15 (0.87-1.53) p=0.33	Multivariate: Site, stage,age
77.	East et al[30] 2014	Retrospective	Colon Cancer	United Kingdom	436<br>Training set, n=386<br>Test set, n=50	NLR ≥3.4	26 patients received adjuvant chemotherapy	N/A	27	N/A	Multivariate: Training set: 1.43 (1.06-1.94) p=0.02<br>Test set: 3.40 (2.64-5.13) p<0.001	Multivariate: N stage, R0 resection, adjuvant treatment, T stage, WLR.

78.	Malietzis et al[177]<br><br>2014	Retrospective	Colorectal Cancer	United Kingdom	506	NLR >3	All patients with neoadjuvant or adjuvant therapy were excluded	28	118	N/A	Multivariate:<br>OR: 1.23 (0.80-1.90)<br>p=0.347	Multivariate:<br>Age at operation, T stage, N stage, surgical approach, ASA score, major complication
79.	Grivas et al[178]<br><br>2014	Retrospective	Renal Cell Carcinoma	Greece	114	NLR ≥2.7	No patients received adjuvant therapy	10	14	N/A	Multivariate:<br>2.866<br>p=0.034	Multivariate:<br>Hb level, Fuhrman grade
80.	Shen et al[179]<br><br>2014	Retrospective	Rectal Cancer	China	199	NLR ≥2.8	All patients treated with neoadjuvant chemoradiotherapy followed by surgery, 184 patients received adjuvant chemotherapy.	N/A	43	N/A	Multivariate:<br>2.123 (1.140-3.954)<br>p=0.018	Multivariate:<br>ypTNM staging, adjuvant chemotherapy
81.	Sun et al[96]<br><br>2014	Retrospective	Colon cancer	China	255	NLR ≥5	No specific mention of neoadjuvant or adjuvant treatment	N/A	94	N/A	Multivariate:<br>RR 1.541 (0.724-3.282)<br>p=0.262	Multivariate:<br>AFP, CEA,fibrinogen, TNM, mGPS
82.	Neofytou et al[180]<br><br>2014	Retrospective	Liver-only Colorectal Metastases	UK	140	NLR >2.4	All patients received neoadjuvant chemotherapy	N/A	59 (5-year survival)	N/A	Multivariate:<br>1.52 (0.78-2.99)<br>p=0.216	Multivariate:<br>No adjuvant chemotherapy
83.	Aurello et al[32]<br><br>2014	Retrospective	Gastric Cancer	Italy	102	NLR ≥5	68 patients received adjuvant chemotherapy after surgery	62	62	N/A	Multivariate:<br>1.51 (0.69-3.28)<br>p=0.29	Multivariate:<br>Prognostic index, mGPS, Tumour stage IV, PI 1&2
84.	Pinato et al[91]<br>2014	Retrospective	Lung	United Kingdom	220	NLR>5	Adjuvant radio and chemotherapy administered	N/A	61	N/A	Multivariate:<br>3.8 (1.6 –8.9) p=0.002	Multivariate:<br>TNM stage, Pleural Effusion
85.	Forrest et al[94]<br><br>2014	Retrospective	Colorectal	United Kingdom	134	NLR>5	No mention of Adjuvant treatment	43	81	Univariate:<br>2.27 (0.99-5.19)<br>p=0.052	N/A	Univariate: T-stage, N-stage, TNM stage, Venous invasion, Peritoneal involvement, Margin involvement, Manual and Automatic Klintrup–Makinen

												grade
86.	Song et al[181]<br><br>2015	Retrospective	Hypopharyngeal SCC	China	146	NLR ≥2.3	14 patients received adjuvant chemoradiotherapy<br><br>94 received adjuvant radiotherapy	N/A	75 (3-year survival)	N/A	Multivariate:<br>2.36 (1.33-4.18)<br>p0.003	Multivariate:<br>Treatment modalities
87.	Xu et al[22]<br>2015	Retrospective	Oesophageal SCC	China	468	NLR>2.40	196 patient received adjuvant chemo and radiotherapy	N/A	259	N/A	Univariate:<br>1.50 (1.17-2.83) p=0.008	Multivariate:<br>Lymph Node Mets, Venous/lymphatic invasion, CRP/Alb Ration
88.	Hirahara[103]<br><br>2015	Retrospective	Oesophageal	Japan	141	NLR≥2.5	No mention of adjuvant treatment	N/A	16	N/A	Univariate:<br>1.164 (0.616-2.126)<br>p=0.631	Multivariate:<br>pStage, GPS
89.	Shibutani[104]<br>2015	Retrospective	Colorectal	Japan	254	NLR>2.5	Adjuvant chemotherapy	N/A	69	N/A	Multivariate:<br>6.599 (0.928-46.914)<br>p=0.059	Multivariate:<br>NLR (Post op), Number of lymph node mets
90.	Takahashi et al[182]<br><br>2015	Retrospective	Non-small Cell Lung Cancer	Japan	342	NLR ≥2.5	Patients who had received neoadjuvant chemotherapy or thoracic irradiation were not included.	N/A	51 (5-year survival)	N/A	Multivariate:<br>2.141 (1.306-3.515)<br>p=0.003	Multivariate:<br>Smoking, CEA,, nonadenocarcinoma, pathological stage, presence of pleural invasion
91.	Tu et al[183]<br><br>2015	Retrospective	Laryngeal Squamous Cell Carcinoma	China	141	NLR >2.17	No mention of adjuvant treatment	N/A	45	N/A	Multivariate :<br>2.177 (1.208-3.924)<br>p=0.010	Multivariate:<br>T classification, lymph node metastasis
92.	Shin et al[184]<br><br>2015	Retrospective	Colorectal Cancer	Korea	269	NLR. ≥3	Patients treated with chemoradiation were excluded	5	N/A	Multivariate:<br>6.190 (1.034-37.047)<br>p=0.046	N/A	Multivariate;<br>Thrombocytosis
93.	Que et al[185]<br><br>2015	Retrospective	Soft-tissue Sarcoma	China	222	NLR ≥2.5	39 patients received adjuvant chemotherapy, 65 patients received adjuvant	N/A	82 (after 150 months)	N/A	Multivariate:<br>1.06 (0.52-2.16)<br>p=0.881	Multivariate:<br>Tumour site, AJCC stage, PLR

							radiotherapy					
94.	Hsu et al[186]<br><br>2015	Retrospective	Gastric Cancer	Taiwan	989	NLR >3.44	499 patients with stage 2 to 4 tumour received chemotherapy	N/A	395 (5-year survival)	N/A	Multivariate:<br>1.565 (1.198-2.044)<br>p=0.001	Multivariate:<br>Resection margin, differentiation, T status, N status, LN ratio, M1 status
95.	Shimizu et al[187]<br><br>2015	Retrospective	Non-small Cell Lung Cancer	Japan	334	NLR ≥2.5	Neither radiotherapy nor chemotherapy administered prior to the surgery	N/A	95 (3 year survival)	N/A	Multivariate:<br>1.60 (1.04-2.54)<br>p=0.048	Multivariate:<br>Age, nodal metastasis, PNI
96.	Han et al[188]<br><br>2015	Retrospective	Glioblastoma	China	152	NLR ≥4	All patients received adjuvant radio-chemotherapy	N/A	118 (2-year survival)	N/A	Multivariate:<br>1.050 (1.003-1.100)<br>p=0.037	Multivariate:<br>KPS, resection, MGMT promoter, PLR
97.	Liao et al[189]<br><br>2015	Retrospective	Hepatocellular Carcinoma	China	222	NLR >2.1	69 patients received transcatheter arterial chemoembolization (TACE) 1 month post surgery.	N/A	77 (5-year survival)	N/A	Multivariate:<br>3.013 (1.633-5.561)<br>p=0.014	Multivariate:<br>Neutrophil count, postoperative TACE
98.	Aldemir et al[190]<br><br>2015	Retrospective	Gastric Cancer	Turkey	53	NLR ≥2.75	No mention of adjuvant treatment	N/A	19	N/A	Univariate:<br>p=0.88	Univariate:<br>ECOG performance status, platelet count
99.	Kadota et al[191]<br><br>2015	Retrospective	Lung Squamous Cell Carcinoma	US	485<br><br>Training cohort n=331	NLR >5.5	80% patients received adjuvant therapy	N/A	Training cohort n=188	N/A	In training cohort Univariate:<br>1.82 (1.26-2.62)<br>p=0.001	Multivariate:<br>Smoking pack-year, pathological stage, CD10/CD20 risk index, age, lymphovascular invasion
100.	Neofytou et al[192]<br><br>2015	Retrospective	Liver-Only Colorectal Metastases	UK	140	NLR (continuous)	All patients received neoadjuvant chemotherapy, 104 received adjuvant chemotherapy.	60	63	Univariate:<br><br>1.20 (1.06-1.36)<br>p=0.003	N/A	Multivariate:<br>Adjuvant chemotherapy, preoperative LMR.

101.	Bagante et al[193]<br><br>2015	Retrospective	Adrenocortical Carcinoma	US	84	NLR >5	51 patients received peri-operative systemic chemotherapy, 38 patients received adjuvant mitotane	50 (5-year CSS)	N/A	Multivariate:<br>2.21 (1.10-4.43)<br>p=0.025	N/A	Multivariate:<br>AJCC tumour status and metastatic status
102.	Wang et al[194]<br><br>2015	Retrospective	Hepatocellular Carcinoma	US	234	NLR >2.5	170 patients had antiviral treatment	N/A	88 (5-year survival)	N/A	Multivariate:<br>4.9 (1.8-13.2)<br>p=0.002	Multivariate:<br>Tumour size
103.	Pine et al[195]<br><br>2015	Retrospective	Colorectal Cancer	United Kingdom	358	NLR ≥5	No mention of adjuvant treatment	N/A	157 (after 4 years)	N/A	Multivariate:<br>1.819 (1.310-2.526)<br>p<0.001	Multivariate:<br>Age, Dukes' stage C and stage D
104.	Li et al[196]<br><br>2015	Retrospective	Endometria Cancer	China	282	NLR ≥4.68	No mention of adjuvant treatment	N/A	38 (5-year survival)	N/A	Multivariate:<br>2.298 (0.679-7.781)<br>p=0.181	Multivariate:<br>CRP, D-dimer,
105.	Zhang et al[197]<br><br>2015	Retrospective	Non-small Cell Lung Cancer	China	678	NLR >2.3	Adjuvant chemotherapy or/and radiotherapy	N/A	367	N/A	Multivariate:<br>1.624 (1.304-2.022)<br>p<0.001	Multivariate:<br>Pathological stage (I,II,IIIA)
106.	Zhang et al[198]<br><br>2015	Retrospective	Gallbladder Carcinoma	China	145	NLR ≥1.94	No mention of adjuvant treatment	N/A	117 (5-year survival)	N/A	Multivariate:<br>RR<br>2.059 (1.253-3.384)<br>p=0.004	Multivariate:<br>Nevin stages, operation modes, Hb
107.	Qu et al[199]<br><br>2015	Retrospective	Gastric Cancer	China	1397<br>Development set: n=1123<br><br>Validation set: n=274	NLR >1.86	All patients underwent neoadjuvant chemotherapy or adjuvant radiotherapy	N/A	3-year survival<br>Development set: 307<br><br>Validation set: 60	N/A	Multivariate:<br>1.379 (1.082-1.758)<br>p=0.009	Multivariate:<br>Age, tumour size, Lauren type, depth of invasion, number of metastatic lymph node.
108.	Zhang et al[200]<br><br>2015	Retrospective	Ovarian Cancer	China	190	NLR >3.4)	Surgery was followed by platinum-based chemotherapy	N/A	170 (after 100-month)	N/A	Univariate:<br>2.172 (1.545-3.054)<br>p<0.001	Multivariate:<br>Stage (FIGO), postoperative residual tumour mass, PLR
109.	Yu et al[201]<br><br>2015	Retrospective	Gastric Cancer	China	291	NLR <3.5	No mention of adjuvant treatment	N/A	199 (5-year survival)	N/A	Multivariate:<br>0.626 (0.460-0.852)<br>p=0.003	Multivariate:<br>N staging, TNM staging

110.	Sun et al[33]<br><br>2015	Retrospective	Gastric Cancer	China	632	NLR. >1.83	395 patients received adjuvant chemotherapy	N/A	448	N/A	Multivariate:<br>1.056 (0.830-1.343) p=0.656	Multivariate:<br>Age, respectability, distant metastasis, pathological stage, CEA, postoperative complications, PNI
111.	Duan et al[202]<br><br>2015	Retrospective	Esophageal SCC	China	371	NLR >3	No mention of adjuvant treatment	192	N/A	Multivariate:<br>1.591 (1.132-2.235) p=0.007	N/A	Multivariate:<br>pN status
112.	Wen et al[203]<br><br>2015	Retrospective	Renal Cell Carcinoma	China	327	NLR ≥1.7	No mention of adjuvant treatment	N/A	230 (after 80 months)	N/A	Multivariate:<br>1.674 (1.103-2.539) p=0.019	Multivariate:<br>Histological subtypes, pT stage
113.	Zhang et al[16]<br><br>2015	Retrospective	Non-Small Cell Lung Cancer	China	1238	NLR >2.3	Adjuvant treatments including chemotherapy, radiotherapy and concurrent chemoradiotherapy	N/A	686	N/A	Univariate:<br>1.533 (1.458-1.785) p<0.001	Multivariate:<br>TNM stage, LDH, D-dimer, COP-NLR
114.	Choi et al[204]<br><br>2015	Retrospective	Colorectal Cancer	Canada	549	NLR≥2.6	147 patients received adjuvant therapy : chemotherapy, radiation or both	N/A	120 (5-year survival)	N/A	Multivariate:<br>1.91 (1.26-2.9) p=0.002	Multivariate:<br>Age>75, lymph nodes positive, ASA status
115.	Deng et al[205]<br><br>2015	Retrospective	Gastric Cancer	China	389	NLR≥2.36	No mention of adjuvant treatment	235	270	Multivariate:<br>1.53 (1.11-2.11) p=0.010	Multivariate:<br>1.13 (0.68-1.87) p=0.648	Multivariate:<br>Age, tumour stage, lymph node, distant metastasis, dNLR
116.	Spolverato et al[206]<br><br>2015	Retrospective	Hepato-Pancreatico-Biliary Malignancies	US	452	NLR ≥5	189 patients received neoadjuvant chemotherapy.	N/A	192 (5-year survival)	N/A	Multivariate:<br>1.94 (1.03-3.64) p=0.040	Multivariate:<br>Age, complications.
117.	Han et al[207]<br><br>2015	Retrospective	Esophageal SCC	China	218	NLR< 2.60	Adjuvant treatment:<br>17 received chemotherapy<br>41 received radiotherapy<br>24 received	N/A	138	N/A	Multivariate:<br>1.133 (0.762-1.685) p=0.538	Multivariate:<br>Tumour length, pTNM stage, LMR.

							chemoradiotherapy				
118.	Kim et al[208]<br><br>2015	Retrospective	Gastric Cancer	Korea	1986	NLR>2	No mention of adjuvant treatment	N/A	323 (5-year survival)	N/A	Multivariate:<br>1.403 (1.048-1.879)<br>p=0.0230	Multivariate:<br>Age, approach method, depth of invasion, node status
119.	Chan et al[209]<br><br>2015	Retrospective	Hepatocellular Carcinoma	Hong Kong	324	NLR≥5	282 patients with chronic viral hepatitis received antiviral therapy	N/A	79 (5-year survival)	N/A	Univariate:<br>1.587 (0.817-3.086)<br>p=0.173	Multivariate:<br>Antiviral therapy, microvascular invasion, PNI.
120.	Choi et al[210]<br><br>2015	Retrospective	Lung Cancer	US	1139	NLR ≥5	Neoadjuvant :<br>245 received chemotherapy<br>18 received radiation<br><br>Adjuvant :<br>285 received chemotherapy<br>170 received radiation	N/A	752 (5-year survival)	N/A	Multivariate:<br>Preoperative NLR<br>1.686 (1.274-2.230)<br>p=0.0003	Multivariate:<br>Age, stage, adjuvant radiation
121.	Lee et al[211]<br><br>2015	Retrospective	Breast cancer	South Korea	3116	NLR ≥ 5.2	No mention of adjuvant treatment	300	N/A	Univariate:<br>1.09 (0.94-1.26)<br>p=0.516	N/A	Multivariate:<br>Post op NLR 1 week, Nuclear grade, AJCC stage, HR status.
122.	Chen et al[212]<br><br>2015	Retrospective	Colorectal Cancer	US	274	NLR >5	No mention of adjuvant treatment	N/A	32 (3-year survival)	N/A	Univariate:<br>2.37 (1.10-5.10)<br>p=0.023	Multivariate:<br>Metastatic site, LDH
123.	Wuxiao et al[213]<br><br>2015	Retrospective	Colon Cancer	China	548	NLR ≤3	All stage 3 patients received 5-fluorouracil based adjuvant chemotherapy	N/A	106	N/A	Multivariate: RR<br>0.384 (0.255-0.580)<br>p<0.001<br><br>Inverted: 2.60 (1.72-3.92)	Multivariate:<br>Histological grade, preoperative CEA levels

124.	Qing Chen et al[214] 2015	Retrospective	Intrahepatic cholangiocarcinoma	China	322	NLR ≥2.49	Patients treated with chemoradiotherapy are removed from this study	N/A	204 (5-year survival)	N/A	Multivariate: 1.600 (1.178-2.174) p=0.003	Multivariate: CA199, tumour number, lymph node metastasis.
125.	Kim et al[215] 2015	Retrospective	Upper Urinary Tract Urothelial Carcinoma	South Korea	277	NLR. ≥5:1	71 patients received adjuvant chemotherapy	73	96	Univariate: 1.179 (0.511-2.718) p=0.700	N/A	Multivariate: Bladder cuff excision, pathologic T stage, lymphovascular invasion, derived NLR
126.	Szkandera et al[216] 2015	Retrospective	Soft Tissue Sarcoma	Austria	340	dNLR ≥2.39	No mention of adjuvant treatment	N/A	98	N/A	Multivariate:, 1.60 (1.07-2.40) p=0.022	Multivariate: Tumour grade
127.	Ben et al[217] 2015	Retrospective	Pancreatic Ductal Adenocarcinoma	China	381	NLR ≥2	No mention of adjuvant treatment	N/A	283	N/A	Multivariate: 1.51 (1.15-1.99) p=0.003	Multivariate: lymphoid node involvement, poor tumour differentiation, edge-positive.
128.	Graziosi et al[218] 2015	Retrospective	Gastric Cancer	Italy	156	NLR. ≥2.34	18 patients received neoadjuvant chemotherapy<br><br>70 patients received adjuvant chemotherapy	N/A	70	N/A	Multivariate: 1.70 (1.02-2.84) p<0.043	Multivariate: Mixed-type Lauren classification
129.	Takahashi et al[219] 2015	Retrospective	Endometrial Cancer	Japan	508	NLR >3	215 patients received adjuvant therapy	50	55	N/A	Univariate: 2.47 (1.45-4.24) p=0.0009	Multivariate: Age, FIGO stage, LVSI, neutrophil count
130.	Shirai et al[220] 2015	Retrospective	Pancreatic cancer	Japan	131	NLR ≥5	No mention of adjuvant treatment	N/A	103 (5-year survival)	N/A	Univariate: 0.984 (0.511-1.894) p=0.961	Multivariate: Tumour size, resection margin status, tumour differentiation, PLR
131.	Chen et al[221] 2015	Retrospective	Intrahepatic Cholangiocarcinoma	China	322	NLR (continuous)	Adjuvant chemoradiotherapy used as well as radiofrequency	N/A	197 (5-year survival)	N/A	Multivariate: 1.399 (1.006-1.947) p=0.046	Multivariate: CA19-9, tumour number, lymph node metastasis,

							ablation					PLR
132.	Neal et al[133]<br><br>2015	Retrospective	Colorectal Liver Metastases	UK	302	NLR ≥5	132 patients had systemic chemotherapy in the 6 months prior to liver resection, 126 patients received systemic chemotherapy following metastasectomy	204 (5-year survival)	214 (5-year survival)	Multivariate: 1.927 (1.398-2.655) p<0.001	Multivariate: 1.769 (1.302-2.403) p<0.001	Multivariate: Clinical risk score
133.	Kawashima et al[41]<br><br>2015	Retrospective	Lung Cancer	Japan	1043	NLR >5	No mention of adjuvant treatment	N/A	227	N/A	Univariate:<br>1.53 (1.00-2.34)<br>p=0.05	Multivariate: Age, smoking, preoperative co-morbidity, CEA, pathological stage, histological tumour type, LVI, surgical procedure
134.	M Cummings et al[19]<br><br>2015	Retrospective	Endometrial Cancer	UK	605	NLR ≥2.4	33% of patients received adjuvant radiotherapy, 13% of patients received adjuvant chemotherapy	96	166	Multivariate:<br>1.68 (1.03-2.76)<br>p=0.04	Multivariate:<br>1.82 (1.27-2.62)<br>p=0.001	Multivariate: PLR, combined NLR + PLR, age, FIGO stage, grade, histopathological subtype, LVSI
135.	Lian et al[222]<br><br>2015	Retrospective	Gastric Cancer	China	162	NLR ≥4.02	No mention of adjuvant treatment	N/A	N/A (expressed in months)	N/A	Univariate:<br>OR 2.58 (1.62-3.80)<br>p=0.001	Multivariate: Depth of invasion, lymph node metastasis, AJCC stage, PLR
136.	Okamura et al[107]<br><br>2015	Retrospective	Hepatocellular Carcinoma	Japan	256	NLR ≥2.81	No mention of adjuvant treatment	N/A	86	N/A	Multivariate:<br>2.41 (1.44-4.01)<br>p=0.001	Multivariate: AFP, des-gamma-carboxy prothrombin, low PNI.
137.	Xie et al[223]<br><br>2016	Retrospective	Oesophageal Squamous Cell Cancer	China	317	NLR. >2.1	76 patients received adjuvant chemotherapy after surgery	147	152	Multivariate:<br>1.196 (0.833-1.719)<br>p=0.332	N/A	Multivariate: PLR, TNM stage

138.	Mohri et al[224] 2016	Retrospective	Gastric Cancer	Japan	404	NLR >3	No mention of adjuvant treatment	65 (5-year survival)	82 (5-year survival)	Multivariate: 1.97 (1.08-3.58) p=0.03	Multivariate: 2.09 (1.10-3.94) p=0.02	Multivariate: Age, gender, ASA, tumour size, p-stage 2 and 3, infectious complication
139.	Ha et al[25] 2016	Retrospective	Ampulla of Vater Cancer	South Korea	227	NLR >1.78	Adjuvant treatments including chemotherapy, radiotherapy and concurrent chemoradiotherapy	N/A	105	N/A	Multivariate: 1.280 (0.70-2.33) p=0.418	Multivariate: Vascular invasion, CA19-9.
140.	Li et al[34] 2016	Retrospective	Colorectal Cancer	China	5336	NLR (≤2.72 vs. >2.72)	5-Fu based adjuvant chemotherapy for stage 2/3 patients	588	611	N/A	Multivariate: 1.227 (1.003-1.501) p=0.047	Multivariate: Age, T stage, N stage, differentiation, venous invasion, LMR, AGR
141.	Takahashi et al[225] 2016	Retrospective	Lung adenocarcinoma	Japan	361	NLR ≥2.5	80 received adjuvant chemotherapy	N/A	74 (5-year survival)	N/A	Multivariate: 1.822 (1.133-2.931) p=0.013	Multivariate: Gender, smoking history, pathological stage, lymphatic/ vascular/ pleural invasion
142.	Cheng et al[226] 2016	Retrospective	Upper Tract Urothelial Carcinoma	Taiwan	195	NLR ≥2.7	35 patients received adjuvant chemotherapy and 16 patients received adjuvant radiation therapy	N/A	55	Multivariate: 1.362 (0.652-2.847) p=0.411	Multivariate: 1.611 (0.890-2.916) p=0.115	Multivariate: WBC, pT stage, tumour grade, RDW
143.	Turner et al[227] 2016	Retrospective	Colon Cancer	Australia	396	NLR >5	Neoadjuvant chemotherapy was an exclusion criteria	N/A	93	N/A	Multivariate: 1.75 (0.87-3.52) p=0.039	Multivariate: Low CIC density, age, ASA score, T4 stage
144.	Fu et al[228] 2016	Retrospective	Laryngeal Squamous Cell Carcinoma	China	420	NLR ≥2.59	Patients needed to have no previous anti-cancer treatment to be included	171 (5-year CSS)	176 (5-year survival)	Multivariate: 1.42 (1.06-1.91) p=0.018	Multivariate: 1.31 (1.00-1.71) p=0.046	Multivariate: Age, drinking, N stage, histological type
145.	Lu et al[229] 2016	Retrospective	Hepatocellular carcinoma	China	963	NLR>2.81	No mention of adjuvant treatment	N/A	553 (5-year survival)	N/A	Multivariate : 1.296 (1.074-1.563) p=0.007	Multivariate : Tumour number, incomplete capsule, serum albumine, ALT, macrovascular

												invasion
146.	Chen et al[230]<br><br>2016	Retrospective	Esophageal Squamous Cell Carcinoma	China	323	NLR >3.5	No mention of adjuvant treatment	221 (5-year)	N/A	Multivariate :<br>1.050 (0.740-1.488)<br>p=0.786	N/A	Multivariate :<br>TNM stage, I stage
147.	Wang et al[231]<br><br>2016	Retrospective	Gastroesophageal Junction and Gastric Adenocarcinoma	US	1498	NLR (continuous)	Neoadjuvant chemotherapy or radiotherapy	588 (5-years)	N/A	Multivariate:<br>1.10 (1.05-1.13)<br>p<0.0001	N/A	Multivariate:<br>T stage, N stage, tumour location
148.	Hodek et al[232]<br><br>2016	Retrospective	Rectal Carcinoma	Czech Republic	173	NLR (continuous)	All patients received neoadjuvant chemoradiotherapy	N/A	22	N/A	Univariate:<br>RR 1.21 (1.03-1.43)<br>p=0.02	Univariate:<br>WBC, RBC, Hb, platelet count, neutrophils, PLR
149.	Christina et al[35]<br><br>2016	Retrospective	Oral cancer	Austria	144	NLR> 1.9	All patients received neoadjuvant radiotherapy in combination with systemic cytotoxic therapy	N/A	60 (5-year survival)	N/A	Univariate:<br>1.16 (0.65-2.06)<br>p=0.62	Multivariate:<br>Regression grade
150.	Morizawa et al[233]<br><br>2016	Retrospective	Bladder cancer	Japan	110	NLR ≥2.6	37 patients received neoadjuvant chemotherapy	32	42	Multivariate:<br>2.6 (1.9-5.2)<br>p=0.01	Multivariate:<br>2.8 (1.4-5.4)<br>p=0.00	Multivariate:<br>ECOG-PS, lymph node metastasis, tumour growth pattern
151.	Ishizuka et al[21]<br><br>2016	Retrospective	Colorectal Cancer	Japan	627	NLR >2.9	No mention of adjuvant treatment	110	142	N/A	Multivariate:<br>1.811 (1.229-2.669)<br>p=0.003	Multivariate:<br>Pathological differentiation, CEA, stage, CAR, GPS
152.	Kosumi et al[234]<br><br>2016	Retrospective	Oesophageal Squamous Cell Carcinoma	Japan	283	NLR ≥1.94	191 patients received adjuvant therapy, 10 patients received neoadjuvant chemoradiotherapy	65	91	Multivariate:<br>1.84 (1.07-3.21)<br>p=0.028	Multivariate:<br>1.84 (1.17-2.93)<br>p=0.0081	Multivariate:<br>Nil else

153.	Kawahara et al[235] 2016	Retrospective	Bladder Cancer	Japan	74	NLR ≥2.38	10 patients received neoadjuvant chemotherapy, 25 patients received adjuvant chemotherapy	N/A	29 (after 4000 days)	N/A	Multivariate: 4.62 (1.16-18.34) p=0.030	Multivariate: CRP, pathological lymph node metastasis.
154.	Wang et al[36] 2016	Retrospective	Ovarian Cancer	China	143	NLR. >3.43)	No mention of adjuvant treatment	N/A	51	N/A	Multivariate: 3.37 (1.39-8.15) p=0.007	Multivariate: Metastasis, prognostic inflammation score
155.	Kang et al[236] 2016	Retrospective	Bladder Cancer	Korea	385	Preop-NLR ≥2.1	96 patients received adjuvant chemotherapy	85	116	Multivariate: 1.16 (1.06-1.28) p=0.005	Multivariate: 1.13 (1.04-1.22) p=0.003	Multivariate: Postop-NLR, pT stage, number of lymph nodes removed, lymph node status, age, surgical margin status
156.	Chan et al[112] 2016	Retrospective	Colorectal Cancer	Australia	1623	NLR. >3.19)	Patients with high-risk stage II and III colon cancer disease were generally offered standard adjuvant chemotherapy, whereas those with stage II or III rectal cancers were usually treated with neoadjuvant chemoradiotherapy	N/A	941	N/A	Univariate: 1.830 (1.539-2.176) p< 0.001	Multivariate: Age, T stage, N stage, grade, LMR
157.	Toyokawa et al[37] 2016	Retrospective	Thoracic Oesophageal Squamous Cell Carcinoma	Japan	185	NLR >3.612	46 patients received neoadjuvant treatment (39 chemotherapy, 6 chemoradiotherapy, 1 radiotherapy)	N/A	77	N/A	Multivariate: 1.194 (0.627-2.273) p=0.589	Multivariate: Sex, performance status, ASA, cTNM stage, CONUT score
158.	Bhindi et al[237] 2016	Retrospective	Bladder Cancer	Canada	418	NLR (per 1-log unit)	28 received neo-adjuvant chemotherapy, 87 received adjuvant	107	177	Multivariate: 1.47 (1.20-1.80) p<0.001	Multivariate: 1.56 (1.16-2.10) p=0.004	Multivariate: T-stage, N-stage, haemoglobin, age, Charlson co-

							chemotherapy, 54 received salvage chemotherapy					morbidity index, lymphovascular invasion

Table 3: Studies investigating the prognostic value of PLR in an unselected cohort of patients with operable cancer (Abbreviations: R-status (Resection margin status), ypN (Post neoadjuvant node status))

No: PLR	Study	Type of Study	Cancer	Country	Patients (n)	Measure of SIR	Additional Treatment	Cancer deaths (n)	Overall deaths (n)	Cancer survival (HR, 95%CI)	Overall survival (HR, 95%CI)	Independent Prognostic Factors
1.	Smith et al[121] 2009	Retrospective	Pancreatic Ductal Adenocarcinoma	UK	110	PLR (continuous)	33 patients had adjuvant therapy	N/A	93 (48-month survival)	N/A	Multivariate: 1.004 (1.002-1.006) p=0.0003	Multivariate: Tumour size, Lymph node ratio
2.	Bhatti et al[125] 2010	Retrospective	Pancreatic ductal adenocarcinoma	UK	84	PLR ≤100, 100-200, >200	30 patients received adjuvant chemotherapy	N/A	66 (3-year survival)	N/A	Univariate: 0.978 (0.899-1.075) 0.642	Multivariate: NLR, Resection margin status
3.	Asher et al[134] 2011	Retrospective	Ovarian Cancer	UK	235	PLR>300	170 patients received chemotherapy	N/A	169 (survival after 150 months)	N/A	Multivariate: 1.698 (1.031-2.797) p=0.03	Multivariate: Age, stage, residual disease
4.	Dutta et al[62] 2011	Retrospective	Oesophagus	UK	112	PLR (<150/ 150-300/ >300)	31 had neoadjuvant and 14 adjuvant therapy	52	59	Univariate: 0.94 (0.60-1.48) p=0.781	N/A	Multivariate: mGPS (0/1/2) lymph node ratio (0/≤0.2/>0.2)
5.	Kwon et al[140] 2012	Retrospective	Colorectal cancer	Korea	200	PLR <150, 150-300, >300	150 patients received adjuvant chemotherapy or chemoradiation	N/A	39	N/A	Multivariate: 1.953 (1.161-3.284) p=0.012	Multivariate: Stage, CEA
6.	Dutta et al[78] 2012	Retrospective	Gastric	UK	120	PLR (<150/ 150-300/ >300)	Patients received both adjuvant and neoadjuvant therapy specific figures not given	44	51	Univariate: 0.83 (0.49-1.40) p=0.483	N/A	Multivariate: Positive lymph node ratio, mGPS,
7.	Carruthers et al[141] 2012	Retrospective	Rectal cancer	UK	115	PLR<160	Neoadjuvant chemoradiation	N/A	43	N/A	Univariate: 1.5 (0.8-2.7) p=0.192	Multivariate: R status, NLR (<5)
8.	Raungkaewmanee et al[238] 2012	Retrospective	Epithelial Ovarian Cancer	Thailand	166	PLR ≥200	145 patients had adjuvant chemotherapy	N/A	50	N/A	Multivariate: 1.41 (0.77-2.56) p=0.263	Multivariate: Stage, surgical outcomes

9.	Wang et al[79] 2012	Retrospective	Gastric	China	324	PLR (<150/ 150-300/ >300)	210 patients had adjuvant chemotherapy	N/A	162	N/A	Univariate: 0.867 (0.665-1.132) p=0.296	Multivariate: The 7th TNM stage, Adjuvant chemotherapy , GPS
10.	Feng et al[18] 2013	Retrospective	Oesophageal Squamous Cell Carcinoma	China	483	PLR >166.5	No mention of adjuvant or neoadjuvant treatment	N/A	244	N/A	Multivariate: 1.751 (1.345-2.280) p<0.001	Multivariate: Differentiation, depth of invasion, node metastasis, CNP
11.	Feng et al[239] 2013	Retrospective	Small Cell Carcinoma of Oesophagus	China	43	PLR ≥150	26 patients received adjuvant chemoradiotherapy	N/A	35	N/A	Multivariate: 2.272 (1.035-4.984) p=0.041	Multivariate: Chemoradiotherapy
12.	Stoz et al[84] 2013	Retrospective	Pancreatic Cancer	Austria	110	PLR≥150	88 Underwent chemotherapy	N/A	110	N/A	Univariate: 1.133 (0.815-1.574) p=0.458	Multivariate: Stage at diagnosis, NLR
13.	Toiyama et al[159] 2013	Retrospective	Rectal Cancer	Japan	84	PLR >150	All patients received neoadjuvant chemoradiotherapy	N/A	37 (after 150 months)	N/A	Univariate: 2.17 (0.90-5.21) p=0.08	Multivariate: Pathological TNM stage, CRP
14.	Son et al[89] 2013	Retrospective	Colon Cancer	Korea	624	PLR>300 vs. <150/ 150-300	503 patients received chemotherapy	N/A	55 (5 yr survival)	N/A	Multivariate: 2.006 (0.530-7.589) p=0.305	Multivariate: Fibrinogen, stage, CEA
15.	Zhang et al[163] 2014	Retrospective	Non-Small Cell Lung Cancer	China	400	PLR ≥171	Patients treated with neoadjuvant and adjuvant therapy were excluded	86	129	N/A	Univariate: 1.985 (1.269-3.104) p=0.003	Multivariate: Age, tumour size
16.	Ying et al[164] 2014	Retrospective	Colorectal Cancer	China	205	PLR≥176	77 colon and 31 rectal cancer patients underwent chemotherapy	100	112	Multivariate: 1.15 (0.75-1.78) p=0.513	Multivariate: 1.15 (0.77-1.73) p=0.501	Multivariate: Grade (G3/G4), chemotherapy
17.	Szkandera et al[160] 2014	Retrospective	Soft Tissue Sarcoma	Austria	340<br><br>Training set, n=170<br><br>Validation set,	PLR ≥200	Training set: 16 received adjuvant chemotherapy, 102 received adjuvant radiotherapy<br><br>Validation set:	Training set: 30<br><br>Validation set: 22	Training set: 53<br><br>Validation set: 51	Univariate: Training set: 2.43 (0.99-5.90) p=0.051<br><br>Validation set: 1.52 (0.66-3.54) p=0.320	Univariate: Training set: 3.02 (0.94-9.70) p=0.019<br><br>Multivariate: Validation set: 0.61 (0.30-1.25)	Multivariate: Age, tumour grade, LMR, tumour size

					n=170		22 received adjuvant chemotherapy, 107 received adjuvant radiotherapy				p=0.175	
18.	Baranyai et al[240]<br><br>2014	Retrospective	Colorectal Cancer	Hungary	336	PLR >300	No mention of adjuvant or neoadjuvant treatment	N/A	335	N/A	Multivariate:<br>3.5 (2.2-5.6)<br>logrank P=3.6e-08 (insignificant)	Multivariate:<br>Elevated platelet count
19.	Jiang et al[171]<br><br>2014	Retrospective	Gastric Cancer	China	377	PLR ≥184	219 patients received adjuvant chemotherapy post gastrectomy	N/A	223	N/A	Multivariate:<br>1.068 (0.791-1.441)<br>p=0.668	Multivariate:<br>Tumour size, serosal invasion, lymph node metastasis, post complication, NLR
20.	Yuan et al[172]<br><br>2014	Retrospective	Adenocarcinoma of Esophagogastric Junction	China	327	PLR <150, 150-300, ≥300	18 patients received neoadjuvant chemotherapy, 59 patients received adjuvant chemotherapy	N/A	185	N/A	Univariate:<br>PLR 150-300: 1.284 (0.897-1.838) p=0.172<br><br>PLR ≥300: 1.398 (0.872-2.241) p=0.164	Multivariate:<br>pTNM stage, adjuvant treatment
21.	Feng et al[174]<br><br>2014	Retrospective	Esophageal SCC	China	483	PLR ≥150	No mention of adjuvant or neoadjuvant treatment	N/A	244	N/A	Multivariate:<br>1.840 (1.407-2.407)<br>p<0.001	Multivariate:<br>Differentiation, depth of invasion, nodal metastasis, NLR
22.	Sun et al[96]<br><br>2014	Retrospective	Colon cancer	China	255	PLR<150, 150-300, >300	No specific mention of neoadjuvant or adjuvant treatment	N/A	94	N/A	Multivariate:<br>RR 0.825 (0.560-1.215)<br>p=0.330	Multivariate:<br>AFP, CEA,fibrinogen, TNM, mGPS
23.	Neofytou et al[180]<br><br>2014	Retrospective	Liver-only Colorectal Metastases	UK	140	PLR >150	All patients received neoadjuvant chemotherapy	N/A	59 (5-year survival)	N/A	Multivariate:<br>2.17 (1.09-4.32)<br>p=0.027	Multivariate:<br>No adjuvant chemotherapy
24.	Krenn-Pilko et al[241]<br><br>2014	Retrospective	Breast cancer	Austria	793	PLR ≥292	712 patients received adjuvant radiotherapy, 93 received adjuvant chemotherapy, 378 received adjuvant hormonal treatment, and 202 received both	136	136	Multivariate:<br>2.03 (1.03-4.02)<br>p=0.042	Multivariate:<br>1.92 (1.01-3.67)<br>p=0.047	Multivariate:<br>Tumour stage, lymph node involvement

							adjuvant chemotherapy and hormonal therapy.					
25.	Szkandera et al[242]<br><br>2014	Retrospective	Colon Cancer	Austria	372	PLR >225	No specific mention of neoadjuvant or adjuvant treatment	N/A	91	N/A	Multivariate:<br>1.49 (0.92-2.40)<br>p=0.107	Multivariate:<br>Nil else
26.	Pinato et al[91]<br>2014	Retrospective	Lung	United Kingdom	220	PLR>300	Adjuvant radio and chemotherapy administered	N/A	61	N/A	Univariate:<br>1.6 (0.6–5.6) p=0.32	Multivariate:<br>TNM I/II/III, Pleural Effusion, NLR
27.	Aurello et al[32]<br><br>2014	Retrospective	Gastric Cancer	Italy	102	PLR <150, 150-300, >300 (0,1,2 respectively)	68 patients received adjuvant chemotherapy after surgery	62	62	N/A	Multivariate:<br>PLR 1:<br>0.43 (0.10-1.73)<br>p=0.23<br><br>PLR 2:<br>1.13 (0.45-2.79)<br>p=0.79	Multivariate:<br>Prognostic index, mGPS
28.	Que et al[185]<br><br>2015	Retrospective	Soft-tissue Sarcoma	China	222	PLR ≥133.915	39 patients received adjuvant chemotherapy, 65 patients received adjuvant radiotherapy	N/A	82 (after 150 months)	N/A	Multivariate:<br>2.60 (1.17-5.74)<br>p=0.019	Multivariate:<br>Tumour site: Trunk & extremity, AJCC stage, PLR
29.	Hsu et al[186]<br><br>2015	Retrospective	Gastric Cancer	Taiwan	989	PLR >132	499 patients with stage 2 to 4 tumour received chemotherapy	N/A	395 (5-year survival)	N/A	Multivariate:<br>0.898 (0.696-1.159)<br>p=0.41	Multivariate:<br>NLR, resection margins, differentiation, T status, N status, LN ratio, M1 status
30.	Sheng Han et al[188]<br><br>2015	Retrospective	Glioblastoma	China	152	PLR >135	All patients received adjuvant radio-chemotherapy	N/A	118 (2-year survival)	N/A	Multivariate:<br>1.003 (0.999-1.007)<br>p=0.152	Multivariate:<br>KPS, MGMT promoter, pre-treatment NLR
31.	Aldemir et al[190]<br><br>2015	Retrospective	Gastric Cancer	Turkey	53	PLR <170 vs. ≥170	No mention of adjuvant treatment	N/A	19	N/A	Univariate:<br>p=0.55	Univariate:<br>ECOG performance status, platelet count

32.	Bagante et al[193]<br><br>2015	Retrospective	Adrenocortical Carcinoma	US	84	PLR >190	51 patients received peri-operative systemic chemotherapy, 38 patients received adjuvant mitotane	50 (5-year DSS)	N/A	Univariate:<br>0.90 (0.47-1.73)<br>p=0.757	N/A	Multivariate:<br>AJCC tumor site, T stage III-IV, Metastasis, NLR
33.	Wang et a[194]<br><br>2015	Retrospective	HCC	US	234	PLR >118.5	170 patients had antiviral treatment	N/A	88 (5-year survival)	N/A	Multivariate:<br>1.6 (0.6-4.3)<br>p=0.3	Multivariate:<br>Tumour size, NLR
34.	Li et al[196]<br><br>2015	Retrospective	Endometrial Cancer	China	282	PLR ≥250	No specific mention of neoadjuvant or adjuvant treatment	N/A	38 (5-year survival)	N/A	Multivariate:<br>0.993 (0.294-3.357)<br>p=0.991	Multivariate:<br>CRP, D-dimer,
35.	Zhang et al[197]<br><br>2015	Retrospective	Non-small Cell Lung Cancer	China	678	PLR >106	Adjuvant chemotherapy or/and radiotherapy	N/A	367	N/A	Multivariate:<br>0.966 (0.761-1.228)<br>p=0.780	Multivariate:<br>Pathological stage (I,II,IIIA), NLR
36.	Zhang et al[198]<br><br>2015	Retrospective	Gallbladder Carcinoma	China	145	PLR ≥113.34	No specific mention of neoadjuvant or adjuvant treatment	N/A	117 (5-year survival)	N/A	Univariate:<br>RR 1.903 (1.309-2.767)<br>p=0.001	Multivariate:<br>Nevin stages, operation modes, Hb, NLR
37.	Qu et al[199]<br><br>2015	Retrospective	Gastric Cancer	China	1397<br><br>Development set: n=1123<br><br>Validation set: n=274	PLR. >168	No specific mention of neoadjuvant or adjuvant treatment	N/A	3-year survival<br><br>Development set: 307<br><br>Validation set: 60	N/A	Univariate:<br>1.762 (1.372-2.264)<br>p<0.001	Multivariate:<br>Age, tumour size, Lauren type, depth of invasion, number of metastatic lymph node, NLR
38.	Zhang et a[200]<br><br>2015	Retrospective	Ovarian Cancer	China	190	PLR >203	Surgery was followed by platinum-based chemotherapy	N/A	170 (after 100-month)	N/A	Multivariate:<br>2.158 (1.468-3.171)<br>p<0.001	Multivariate:<br>Stage (FIGO), postoperative residual tumour mass
39.	Zhang et al[243]<br><br>2015	Retrospective	Bladder cancer	China	124	PLR ≥140	No mention of adjuvant treatment	N/A	55 (5-year survival)	N/A	Multivariate:<br>1.161(0.605-2.226)<br>p=0.654	Multivariate:<br>Diabetes, T staging, distant metastasis, LMR

40.	Sun et al[33] 2015	Retrospective	Gastric Cancer	China	632	PLR >140	395 patients received adjuvant chemotherapy	N/A	448	N/A	Multivariate: 1.190 (0.960-1.475) p=0.113	Multivariate: Age, respectability, distant metastasis, pathological stage, CEA, postoperative complications, PNI
41.	Choi et al[204] 2015	Retrospective	Colorectal Cancer	Canada	549	PLR≥295	147 patients received adjuvant therapy : chemotherapy, radiation or both	N/A	120 (5-year survival)	N/A	Univariate: 1.81 (1.06-3.06) p=0.028	Multivariate: Age>75, lymph nodes positive, ASA status, NLR
42.	Deng et a[205] 2015	Retrospective	Gastric Cancer	China	389	PLR≥132	No mention of adjuvant treatment	235	270	Multivariate: 0.96 (0.71-1.28) p=0.763	Multivariate: 1.03 (0.78-1.35) p=0.858	Multivariate: Age, tumour stage, lymph node, distant metastasis, dNLR
43.	Spolverato et al[206] 2015	Retrospective	Hepato-Pancreatico-Biliary Malignancies	US	452	PLR ≥190	189 patients received neoadjuvant chemotherapy.	N/A	192 (5-year survival)	N/A	Multivariate: 1.79 (1.05-3.04) p=0.032	Multivariate: Age, complications, NLR
44.	Han et al[207] 2015	Retrospective	Esophageal SCC	China	218	PLR<244	Adjuvant treatment: 17 received chemotherapy 41 received radiotherapy 24 received chemoradiotherapy	N/A	138	N/A	Multivariate: 1.014 (0.582-1.769) p=0.96	Multivariate: Tumour length, pTNM stage, LMR.
45.	Kim et al[208] 2015	Retrospective	Gastric Cancer	Korea	1986	PLR>126	No mention of adjuvant treatment	N/A	323 (5-year survival)	N/A	Multivariate: 1.035 (0.805-1.330) p=0.7888	Multivariate: Age, approach method, depth of invasion, node status, NLR
46.	Anthony et al[209] 2015	Retrospective	Hepatocellular Carcinoma	Hong Kong	324	PLR≥150	282 patients with chronic viral hepatitis received antiviral therapy	N/A	79 (5-year survival)	N/A	Univariate: 1.229 (0.756-1.998) p=0.405	Multivariate: Antiviral therapy, microvascular invasion, PNI.
47.	Kim et al[215] 2015	Retrospective	Upper Urinary Tract Urothelial Carcinoma	South Korea	277	PLR <150, 150-300, >300	71 patients received adjuvant chemotherapy	73	96	Univariate: PLR 150-300 1.460 (0.887-2.405)	N/A	Multivariate: Bladder cuff excision, pathologic T stage,

										p=0.137<br><br>PLR >300 1.202 (0.374-3.864) p=0.757		lymphovascular invasion, derived NLR
48.	Neofytou et al[192]<br><br>2015	Retrospective	Liver-Only Colorectal Metastases	UK	140	PLR (continuous variable)	All patients received neoadjuvant chemotherapy, 104 received adjuvant chemotherapy.	60	63	Univariate:<br>1.006 (1.002-1.009)<br>p<0.001	N/A	Multivariate:<br>Adjuvant chemotherapy, neoadjuvant LMR.
49.	Messager et al[244]<br><br>2015	Retrospective	Oeophageal and junctional carcinoma	UK	153	PLR >192	36.6% of patients received adjuvant chemotherapy after surgery	N/A	39	N/A	Multivariate:<br>2.47 (1.21-5.01)<br>p=0.012	Multivariate:<br>Differentiation, resection margin, ypN
50.	Pang et al[245]<br><br>2015	Retrospective	Gallbladder carcinoma	China	316	PLR≥ 117.7	No mention of adjuvant or neoadjuvant treatment	N/A	254	N/A	Multivariate:<br>2.021 (1.243-3.278)<br>p=0.005	Multivariate:<br>CA-125, CA-199,TNM
51.	Ozawa et al[246]<br><br>2015	Retrospective	Colorectal Cancer	Japan	234	PLR ≥25.4	15 patients excluded as underwent adjuvant chemotherapy	222	211	Multivariate:<br>3.61 (1.08-12.64)<br>p=0.038	N/A	Multivariate:<br>Nil else
52.	Shirai et al[220]<br><br>2015	Retrospective	Pancreatic cancer	Japan	131	PLR ≥150	No mention of adjuvant treatment	N/A	103 (5-year survival)	N/A	Multivariate:<br>1.688 (1.045-2.726)<br>p=0.032	Multivariate:<br>Tumour size, resection margin status, tumour differentiation
53.	Chen et al[221]<br><br>2015	Retrospective	Intrahepati c Cholangioc arcinoma	China	322	PLR ≥123	Adjuvant chemoradiotherapy used as well as radiofrequency ablation	N/A	197 (5-year survival)	N/A	Multivariate:<br>1.410 (1.026-1.938)<br>p=0.034	Multivariate:<br>CA19-9, tumour number, lymph node metastasis, NLR
54.	Neal et al[17]<br><br>2015	Retrospective	Colorectal Liver Metastases	UK	302	PLR <150, 150-300, >300	132 patients had systemic chemotherapy in the 6 months prior to liver resection, 126 patients received systemic chemotherapy following metastasectomy	204 (5-year survival)	214 (5-year survival)	Univariate:<br>1.244 (1.003-1.542) p=0.047	Univariate:<br>1.244 (1.015-1.525)<br>p=0.036	Multivariate:<br>Clinical risk score, NLR≥3

55.	Xu et al[22]<br><br>2015	Retrospective	Oesophageal SCC	China	468	PLR>147	196 patient received adjuvant chemo and radiotherapy	N/A	259	N/A	Univariate:<br>1.12 (0.87-1.43) p=0.39	Multivariate:<br>Lymph Node Mets, Venous/lymphatic invasion, CRP/Alb Ratio
56.	Kawashima et al[41]<br><br>2015	Retrospective	Lung Cancer	Japan	1043	PLR >300	No mention of adjuvant treatment	N/A	227	N/A	Univariate:<br>2.35 (1.45-3.82) p<0.01	Multivariate:<br>Age, smoking, neoadjuvant co-morbidity, CEA, pathological stage, histological tumour type, LVI, surgical procedure
57.	Cummings et al[19]<br><br>2015	Retrospective	Endometrial Cancer	UK	605	PLR. ≥240	33% of patients received adjuvant radiotherapy, 13% of patients received adjuvant chemotherapy	96	166	Multivariate:<br>1.76 (1.09-2.87) p=0.022	Multivariate:<br>1.89 (1.30-2.75) p=0.001	Multivariate:<br>NLR, Combined NLR + PLR, age, FIGO stage, grade, histopathological subtype, LVSI
58.	Lian et al[222]<br><br>2015	Retrospective	Gastric Cancer	China	162	PLR ≥208	No mention of adjuvant treatment	N/A	N/A (expressed in months)	N/A	Multivariate:<br>OR 2.55 (1.37-3.84) p=0.001	Multivariate:<br>Depth of invasion, lymph node metastasis, AJCC stage
59.	Saito et al[247]<br><br>2016	Retrospective	Perihilar cholangiocarcinoma	Japan	115	PLR >150	1 patient received neoadjuvant chemotherapy, 1 patient received neoadjuvant radiation, 1 patient received neoadjuvant chemotherapy and radiation, 21 patients received adjuvant therapy	N/A	59 (5-year survival)	Multivariate:<br>2.207 (1.200-4.060) p=0.011	N/A	Multivariate:<br>Preoperative factors (CEA, albumin, CRP), N category, portal vein invasion, surgical margin
60.	Xie et al[223]<br><br>2016	Retrospective	Oesophageal Squamous Cell Cancer	China	317	PLR >103	76 patients received adjuvant chemotherapy	147	152	Multivariate:<br>1.776 (1.224-2.578) p=0.003	N/A	Multivariate:<br>TNM stage
61.	Bhindi et al[237]<br><br>2016	Retrospective	Bladder Cancer	Canada	418	PLR per 100 units	28 received neo-adjuvant chemotherapy, 87 received adjuvant chemotherapy, 54	107	177	Univariate:<br>1.21 (1.05-1.41) p=0.01	Univariate:<br>1.16 (1.02-1.33) p=0.03	Multivariate:<br>T-stage, N-stage, haemoglobin, NLR, age, Charlson co-morbidity

							received salvage chemotherapy					index, lymphovascular invasion
62.	Ha et al[25] 2016	Retrospective	Ampulla of Vater Cancer	South Korea	227	PLR >192	Adjuvant treatments including chemotherapy, radiotherapy and concurrent chemoradiotherapy	N/A	105	N/A	Multivariate: 0.686 (0.35-1.34) p=0.268	Multivariate: Vascular invasion, CA19-9.
63.	Li et al[34] 2016	Retrospective	Colorectal Cancer	China	5336	PLR >219	5-Fu based adjuvant chemotherapy for stage 2/3 patients	588	611	N/A	Multivariate: 1.175 (0.946-1.460) p=0.144	Multivariate: Age, T stage, N stage, differentiation, venous invasion, NLR, LMR, AGR
64.	Chen et al[230] 2016	Retrospective	Esophageal Squamous Cell Carcinoma	China	323	PLR >150	No mention of adjuvant treatment	221 (5-year)	N/A	Multivariate : 1.440 (0.978-2.121) p=0.064	N/A	Multivariate : TNM stage, I stage
65.	Hodek et al[232] 2016	Retrospective	Rectal Carcinoma	Czech Republic	173	PLR (continuous)	All patients received neoadjuvant chemoradiotherapy	N/A	22	N/A	Univariate: RR : 1.01 (1.00-1.01) p=0.02	Univariate: Clinical T stage, circular vs semi-circular, stenotizing tumour, LVSI, angioinvasion, perineural invasion, R0 resection, positive lymph nodes, tumour stage, WBC, RBC, Hb, platelet count, neutrophils, NLR
66.	Wang et al[36] 2016	Retrospective	Ovarian Cancer	China	143	PLR >201	No mention of adjuvant treatment	N/A	51	N/A	Univariate: 1.76 (1.02-3.06) p=0.043	Multivariate: Metastasis, prognostic inflammation score

67.	Chan et al[112]<br><br>2016	Retrospective	Colorectal Cancer	Australia	1623	PLR >258	Patients with high-risk stage II and III colon cancer disease received adjuvant chemotherapy. Stage II or III rectal cancers received neoadjuvant chemoradiotherapy	N/A	941	N/A	Univariate:<br>1.592 (1.343-1.886)<br>p< 0.001	Multivariate:<br>Age, T stage, N stage, grade, LMR
68.	Toyokawa et al[202]<br><br>2016	Retrospective	Thoracic Oesophageal Squamous Cell Carcinoma	Japan	185	PLR >193	46 patients received neoadjuvant treatment (39 chemotherapy, 6 chemoradiotherapy, 1 radiotherapy)	N/A	77	N/A	Multivariate:<br>1.213 (0.696-2.115)<br>p=0.496	Multivariate:<br>Sex, performance status, ASA, cTNM stage, CONUT score

Table 4: Studies investigating the prognostic value of LMR in an unselected cohort of patients with operable cancer (Abbreviations: HER-2 (Herceptin 2 receptor)

**No: LMR**	**Study**	**Type of Study**	**Cancer**	**Country**	**Patients (n)**	**Measure of SIR**	**Additional Treatment**	**Cancer deaths (n)**	**Overall deaths (n)**	**Cancer survival (HR, 95%CI)**	**Overall survival (HR, 95%CI)**	**Independent Prognostic Factors**
1.	Stotz et al[248] 2013	Retrospective	Colon Cancer	Austria	372	LMR ≥ 2.14	230 patients received adjuvant chemotherapy	N/A	72	N/A	Multivariate: 0.51 (0.31-0.83) p=0.007	Multivariate: Tumour invasion depth, lymph node involvement, tumour stage
2.	Szkandera et al[160] 2014	Retrospective	Soft Tissue Sarcoma	Austria	340<br>Training set, n=170<br>Validatio n set, n=170	LMR ≥2.85	Training set: 16 received adjuvant chemotherapy, 102 received adjuvant radiotherapy<br>Validation set: 22 received adjuvant chemotherapy, 107 received adjuvant radiotherapy	Training set: 30 Validation set: 22	Training set: 53 Validation set: 51	Multivariate: Training set: 0.41 (0.18-0.97) p=0.043<br>Validation set: 0.33 (0.12-0.90) p=0.030	Multivariate: Training set: 0.72 (0.34-1.52) p=0.390<br>Validation set: 0.35 (0.17-0.75) p=0.007	Multivariate: Age, tumour grade, LMR, tumour size
3.	Hu et al[249] 2014	Retrospective	Lung Cancer	China	1453	LMR ≤3.68	No mention of adjuvant treatment	N/A	509	N/A	Multivariate: 1.510 (1.265-1.803) p<0.001	Multivariate: Age, TNM stage
4.	Zhou et al[250] 2014	Retrospective	Gastric Cancer	China	426	LMR ≥4.32	306 patients received adjuvant chemotherapy	N/A	250	N/A	Multivariate: 0.688 (0.521-0.908) p=0.008	Multivariate: Size, vascular/ nerve infiltration, TNM stage, adjuvant chemotherapy
5.	Hutterer et al[251] 2014	Retrospective	Clear Cell Renal Cell Carcinoma	Austria	678	LMR <3	No mention of adjuvant treatment	68	123	Multivariate: 2.332 (1.100-4.942) p=0.027	Multivariate: 1.373 (0.929-2.031) p=0.112	Multivariate: Age, pathologic T category, tumour grade, tumour necrosis
6.	Zhang et al[243] 2015	Retrospective	Bladder cancer	China	124	LMR ≥4	No mention of adjuvant treatment	N/A	55 (5-year survival)	N/A	Multivariate: 0.674 (0.412-0.890) p=0.003	Multivariate: Diabetes, T staging, distant metastasis, PLR

7.	Han et al[207]<br><br>2015	Retrospective	Esophageal SCC	China	218	LMR<2.57	Adjuvant treatment:<br>17 received chemotherapy<br><br>41 received radiotherapy<br><br>24 received chemoradiotherapy	N/A	138	N/A	Multivariate:<br>1.759 (1.201-2.576)<br>p=0.004	Multivariate:<br>Tumour length, pTNM stage.
8.	Deng et al[205]<br><br>2015	Retrospective	Gastric Cancer	China	389	LMR≥4.95	No mention of adjuvant treatment	235	270	Multivariate:<br>1.00 (0.71-1.40)<br>p=0.995	Multivariate: 1.00 (0.73-1.35) p=0.977	Multivariate:<br>Age, tumour stage, lymph node, distant metastasis, dNLR
9.	Neofytou et al[192]<br><br>2015	Retrospective	Liver-Only Colorectal Metastases	UK	140	Preoperative LMR ≤3	All patients received neoadjuvant chemotherapy, 104 received adjuvant chemotherapy.	60	63	Multivariate:<br>2.15 (1.13-4.10)<br>p=0.020	Multivariate:<br>2.43 (1.32-4.48)<br>p=0.004	Multivariate:<br>Adjuvant chemotherapy, preoperative
10.	Neal et al[17]<br><br>2015	Retrospective	Colorectal Liver Metastases	UK	302	LMR >2.35	132 patients had systemic chemotherapy in the 6 months prior to liver resection, 126 patients received systemic chemotherapy following metastasectomy	204 (5-year survival)	214 (5-year survival)	Univariate:<br>0.624 (0.455-0.855) p=0.003	Univariate:<br>0.638 (0.473-0.860)<br>p=0.003	Multivariate:<br>Clinical risk score
11.	Wen et al[252]<br><br>2015	Retrospective	Breast Cancer	China	2000	LMR cut-off 3.80 (low or high-LMR)	No mention of adjuvant therapy but likely triple negative cancers had chemo	N/A	326	N/A	Multivariate:<br>0.840 (0.629-1.121)<br>p=0.236	Multivariate:<br>Menstrual status, tumour size, lymph node status ER,HER-2, monocyte count
12.	Lin et al[253]<br><br>2015	Retrospective	HCC	China	210	LMR >3.23	Antiviral therapy for all patients after surgery	47	48	N/A	Multivariate:<br>0.398 (0.219-0.725)<br>p=0.003	Multivariate:<br>Liver cirrhosis, ALP, microvascular invasion, histological differentiation, BCLC stage

13.	Yoshida et al[254] 2015	Retrospective	Bladder Cancer	Japan	181	LMR <3.51	44 patients received adjuvant chemotherapy	58	70	N/A	Multivariate: 3.77 (2.19-6.48) p<0.001	Multivariate: pT-stage, pN-stage, positive margin
14.	Yamagishi et al[255] 2015	Retrospective	Malignant Pleural Mesothelio ma	Japan	44	LMR <2.74	Chemotherapy administered in 57.3% of people	N/A	28	N/A	Multivariate: 2.34 (1.58-3.47) p<0.0001	Multivariate: Histological subtype, ECOG, Stage, Surgery
15.	Ozawa et al[256] 2015	Retrospective	Colorectal Cancer	Japan	117	LMR <3	53 patients received adjuvant chemotherapy	24 (3-year death rate)	N/A	Multivariate: 2.75 (1.40-5.44) p=0.004	N/A	Multivariate: Nil Else
16.	Hutterer et al[257] 2015	Retrospective	Upper Tract Urothelial Carcinoma	Austria	182	LMR ≥2	No mention of adjuvant treatment	N/A	82	N/A	Multivariate: 0.56 (0.35-0.92) p=0.021	Multivariate: Age, pathological T stage
17.	Huang et al[258] 2015	Retrospective	Oesophage al Squamous Cell Carcinoma	China	348	LMR >2.93	105 patients received adjuvant therapy	129	N/A	Multivariate: 0.600 (0.407-0.885) p=0.010	N/A	Multivariate: Depth of invasion, nodal metastasis, lymphocyte count
18.	Chen et al[259] 2015	Retrospective	Cervical Cancer	China	485	LMR >2.87	63 patients received radiotherapy, 315 received chemoradiotherapy	N/A	64	N/A	Multivariate: 0.417 (0.244-0.714) p=0.001	Multivariate: Lymph node metastasis
19.	Bhindi et al[237] 2016	Retrospective	Bladder Cancer	Canada	418	LMR per 1-log unit	28 received neo-adjuvant chemotherapy, 87 received adjuvant chemotherapy, 54 received salvage chemotherapy	107	177	Univariate: 0.69 (0.53-0.91) p=0.009	Univariate: 0.70 (0.55-0.88) p=0.002	Multivariate: T-stage, N-stage, haemoglobin, NLR, age, Charlson co-morbidity index, lymphovascular invasion
20.	Li et al[34] 2016	Retrospective	Colorectal Cancer	China	5336	LMR >2.83	5-Fu based adjuvant chemotherapy for stage 2/3 patients	588	611	N/A	Multivariate: 0.761 (0.621-0.932) p=0.008	Multivariate: Age, T stage, N stage, differentiation, venous invasion, NLR, AGR
21.	Chan et al[112] 2016	Retrospective	Colorectal Cancer	Australia	1623	LMR >2.38	Patients with high-risk stage II and III colon cancer disease were generally offered standard	N/A	941	N/A	Multivariate: 0.569 (0.478-0.677) p< 0.001	Multivariate: Age, T stage, N stage, grade

							adjuvant chemotherapy, whereas those with stage II or III rectal cancers were usually treated with neoadjuvant chemoradiotherapy					

Table 5: Studies investigating the prognostic value of other scores in an unselected cohort of patients with operable cancer (Abbreviations: RFA (Radiofrequency ablation), TACE (Trans arterial chemoembolization)

No: Other	Study	Type of Study	Cancer	Country	Patients (n)	Measure of SIR	Additional Treatment	Cancer deaths (n)	Overall deaths (n)	Cancer survival (HR, 95%CI)	Overall survival (HR, 95%CI)	Independent Prognostic Factors
1.	Miyata et al[24] 2011	Retrospective	Esophageal Cancer	Japan	152	Systemic inflammation score (0-1 vs. 2-3) involving leucocyte count, serum albumin and haemoglobin level	All patients received pre-operative chemotherapy	N.A	92 (5-year survival)	N/A	Multivariate: 3.17 (1.74-5.78) p=0.0002	Multivariate: Clinical response, number of metastatic lymph nodes, operative complication
2.	Tomita et al[27] 2012	Retrospective	Non Small Cell Lung Cancer	Japan	301	NLR and CRP combined	No mention of adjuvant treatment	N/A	N/A (expressed in %)	N/A	Multivariate: Both low/ both high Risk ratio 0.403 (0.240-0.689) p=0.0012<br><br>Either high/ both high Risk ratio 0.452 (0.225-0.872) p=0.0177	Multivariate: pT status, pN status, CEA.
3.	Feng et al[18] 2013	Retrospective	Oesophageal Squamous Cell Carcinoma	China	483	CNP (1-2 vs. 0) involving NLR and PLR	No mention of adjuvant or neoadjuvant treatment	N/A	244	N/A	Multivariate: 1.964 (1.371-2.814) p<0.001	Multivariate: Differentiation, depth of invasion, node metastasis, PLR
4.	Ishizuka et al[14] 2013	Retrospective	Colorectal	Japan	481	COP-NLR (1, 2/0)	Patients with stage IV disease had chemotherapy	120	150	Multivariate: OR: 0.464 (0.267-0.807) p=0.007	N/A	Pathology, LN Mets, CRP, Albumin, CEA, GPS
5.	Peng et al[260] 2014	Retrospective	HCC	China	219	ΔPLR ≥2.875	No specific mention of neoadjuvant or adjuvant treatment	N/A	40	N/A	Multivariate: 5.929 (2.823-12.448) p<0.001	Multivariate: Vascular invasion
6.	Peng et al[261] 2014	Retrospective	Small hepatocellular carcinoma	China	189	ΔNLR (postoperative minus preoperative NLR)	68 patients received adjuvant therapy after operation (TACE, RFA, sorafenib)	N/A	37	N/A	Multivariate: 2.637 (1.356-5.128) p=0.004	Vascular invasion, postoperative NLR.
7.	Ishizuka et al[15] 2014	Retrospective	Gastric Cancer	Japan	544	COP-NLR (0, 1/2)	343 patients received adjuvant chemotherapy	55	108	N/A	Multivariate: 1.781 (1.094-2.899) p=0.020	Multivariate: Age, tumour type, lymph node metastasis, albumin, COP-NLR

8.	Hyun Hwan Sung[29]<br><br>2014	Retrospective	Upper Urinary Tract Urothelial Carcinoma	Korea	410	Inflammation risk score (none, I, II) involving NLR and ESR	91 patients received adjuvant chemotherapy post operation	67	118	Multivariate:<br>Score I 2.785 (1.343-5.776) p=0.006<br><br>Score II 4.367 (1.987-9.597) p<0.001	Multivariate:<br>Score I 2.513 (1.434-4.405)<br>p=0.001<br><br>Score II 3.521 (1.888-6.567) p<0.001	Multivariate:<br>Age, tumour stage, lymph node, margin, micropapillary variant
9.	East et al[30]<br><br>2014	Retrospective	Colon Cancer	United Kingdom	436<br>Training set, n=386<br><br>Test set, n=50	White cell count/ lymphocyte ratio (WLR) ≥3.4	26 patients received adjuvant chemotherapy	N/A	27	N/A	Multivariate:<br>Training set: 1.40 (1.04-1.89) p=0.03<br><br>Test set: 4.10 (3.13-7.42) p=0.03	Multivariate:<br>N stage, R0 resection, adjuvant treatment, T stage, NLR.
10.	Shen et al[31]<br><br>2014	Retrospective	HCC	China	332	AST-platelet ratio index (APRI) <0.62 vs. ≥0.62	No mention of adjuvant or neoadjuvant treatment	N/A	209	N/A	Multivariate:<br>1.508 (1.127-2.016)<br>p=0.006	Multivariate:<br>APRI, tumour size, noncapsulation, tumour number
11.	Aurello et al[32]<br><br>2014	Retrospective	Gastric Cancer	Italy	102	Prognostic index (PI) 0/1/2 involving C-reactive protein and white cell count<br><br>Prognostic nutrition index (PNI) 0/1 involving albumin and total lymphocyte count	68 patients received adjuvant chemotherapy after surgery	62	62	N/A	Multivariate:<br>PI 1:<br>0.04 (0.01-0.20)<br>p< 0.001<br><br>PI 2:<br>0.37 (0.16-0.82)<br>p=0.01<br><br>Univariate:<br>PNI 0/1:<br>0.52 (0.26-1.04)<br>p=0.06	Multivariate:<br>mGPS
12.	Takeno et al[42]<br><br>2014	Retrospective	Gastric	Japan	552	HS-mGPS (0/1/2)	No mention of adjuvant treatment	N/A	215	N/A	Multivariate:<br>1.6748 (1.2867-2.1314)<br><br>p= 0.0002	Multivariate:<br><br>HS-mGPS
13.	Cummings et al[19]<br><br>2015	Retrospective	Endometrial Cancer	UK	605	MLR <0.19 vs. ≥0.19	33% of patients received adjuvant radiotherapy, 13% of patients received adjuvant chemotherapy	96	166	Multivariate:<br>1.26 (0.73-2.15)<br>p=0.409	Multivariate:<br>1.23 (0.84-1.82)<br>p=0.294	Multivariate:<br>PLR, combined NLR + PLR, age, FIGO stage, grade, histopathological subtype, LVSI

14.	Shimizu et al[187]<br><br>2015	Retrospective	Non-small Cell Lung Cancer	Japan	334	Prognostic nutritional index <50 vs. ≥50	Neither radiotherapy nor chemotherapy administered prior to the surgery	N/A	95 (3-year survival)	N/A	Multivariate:<br>2.40 (1.39-4.14<br>p=0.002	Multivariate:<br>Age, nodal metastasis, NLR
15.	Qin Wang et al[194]<br><br>2015	Retrospective	Hepatitis B-Associated Hepatocellular Carcinoma	US	234	Prognostic nutritional index >50.5	170 patients had antiviral treatment	N/A	88 (5-year survival)	N/A	Multivariate:<br>1.3 (0.5-3.4)<br>p=0.5	Multivariate:<br>Tumour size, NLR
16.	Sun et al[33]<br><br>2015	Retrospective	Gastric Cancer	China	632	Prognostic nutritional index <48.2 vs. ≥48.2	395 patients received adjuvant chemotherapy	N/A	448	N/A	Multivariate:<br>1.668 (1.368-2.035)<br>p=0.656	Multivariate:<br>Age, respectability, distant metastasis, pathological stage, CEA, postoperative complications
17.	Sun et al[33]<br><br>2015	Retrospective	Gastric Cancer	China	632	Canton score (0/1/2/3)	395 patients received adjuvant chemotherapy	N/A	448	N/A	Multivariate:<br>Canton score 1<br>1.076 (0.796-1.454)<br>p=0.633<br><br>Canton score 2<br>1.554 (1.151-2.097)<br>p=0.004<br><br>Canton score 3<br>1.643 (1.142-2.364)<br>p=0.007	Multivariate:<br>Resectability,
18.	Zhang et al[16]<br><br>2015	Retrospective	Non-Small Cell Lung Cancer	China	1238	Combination of neoadjuvant platelet count and neutrophil-lymphocyte ratio<br><br>COP-NLR (0/1/2)	Adjuvant treatments including chemotherapy, radiotherapy and concurrent chemoradiotherapy	N/A	686	N/A	Multivariate:<br>1.810 (1.587-2.056)<br>p<0.001	Multivariate:<br>TNM stage, LDH, D-dimer, COP-NLR
19.	Chan et al[209]<br><br>2015	Retrospective	Hepatocellular Carcinoma	Hong Kong	324	Prognostic nutritional	282 patients with chronic viral hepatitis received antiviral therapy	N/A	79 (5-year survival)	N/A	Multivariate:<br>2.778 (1.630-4.813)	Multivariate:<br>Antiviral therapy, microvascular invasion

						index < 45					p<0.001	
20.	Kim et al[215]<br><br>2015	Retrospective	Upper Urinary Tract Urothelial Carcinoma	South Korea	277	PNI ≥45 vs. <45	71 patients received adjuvant chemotherapy	73	96	Multivariate:<br>0.947 (0.491-1.826) p=0.870	N/A	Multivariate:<br>Bladder cuff excision, pathologic T stage, lymphovascular invasion, derived NLR
21.	Neal et al[17]<br><br>2015	Retrospective	Colorectal Liver Metastases	UK	302	COP-NLR (2/1/0) :Combination of platelet count and neutrophil lymphocyte ratio	132 patients had systemic chemotherapy in the 6 months prior to liver resection, 126 patients received systemic chemotherapy following metastasectomy	204 (5-year survival)	214 (5-year survival)	Univariate:<br>1.243 (1.003-1.541) p=0.047	Univariate:<br>1.230 (1.005-1.505) p=0.045	Multivariate:<br>Clinical risk score
22.	Neal et al[17]<br><br>2015	Retrospective	Colorectal Liver Metastases	UK	302	Prognostic nutritional index (0/1)	132 patients had systemic chemotherapy in the 6 months prior to liver resection, 126 patients received systemic chemotherapy following metastasectomy	204 (5-year survival)	214 (5-year survival)	Univariate:<br>0.657 (0.437-0.988) p=0.043	Univariate:<br>0.707 (0.475-1.053) p=0.088	Multivariate:<br>Clinical risk score
23.	Cummings et al[19]<br><br>2015	Retrospective	Endometrial Cancer	UK	605	Combined NLR + PLR<br><br>(both low, either high, both high)	33% of patients received adjuvant radiotherapy, 13% of patients received adjuvant chemotherapy	96	166	Multivariate:<br>Either high:<br>1.46 (0.87-2.47)<br>p=0.156<br><br>Both high:<br>2.26 (1.24-4.13)<br>p=0.008	Multivariate:<br>Either high:<br>1.59 (1.08-2.35)<br>p=0.018<br><br>Both high:<br>2.54 (1.61-4.01)<br>p<0.001	Multivariate:<br>Age, FIGO stage, grade, histopathological subtype, LVSI
24.	Ishizuka et al[21]<br><br>2015	Retrospective	Colorectal Cancer	Japan	627	CRP/ albumin ratio (CAR) >0.038 vs. ≤0.038	No mention of adjuvant treatment	110	142	N/A	Multivariate:<br>2.613 (1.621-4.212)<br>p< 0.001	Multivariate:<br>Pathological differentiation, CEA, stage, GPS, NLR

25.	Chen et a[28]<br><br>2015	Retrospective	Gastric Carcinoma	China	1332<br><br>Training set: 888<br><br>Validation set: 444	Neoadjuvant haemoglobin, albumin, lymphocyte and platelet (HALP) <56.8 vs. ≥56.8	No mention of adjuvant treatment	N/A	581	N/A	Multivariate:<br>Training set:<br>0.782 (0.617-0.993)<br>p=0.043<br><br>Validation set:<br>0.700 (0.496-0.987)<br>p=0.042	Multivariate:<br>Age, longitudinal location, tumour size, N stage, M stage
26.	Okamura et al[107]<br><br>2015	Retrospective	Hepatocellular Carcinoma	Japan	256	Prognostic nutritional index <48.5 vs. ≥48.5	No mention of adjuvant treatment	N/A	86	N/A	Multivariate:<br>1.96 (1.21-3.18)<br>p=0.006	Multivariate:<br>AFP, des-gamma-carboxy prothrombin, high NLR
27.	Xu et al[22]<br><br>2015	Retrospective	Oesophageal SCC	China	468	CRP/Albumin Ratio >0.50	196 patient received adjuvant chemo and radiotherapy	N/A	259	N/A	Multivariate:<br>2.44 (1.82-3.26)<br>p<0.0001	Multivariate:<br>Lymph Node Mets, Venous/lymphatic invasion, CRP/Alb Ration
28.	Chuan Li et al[20]<br><br>2015	Retrospective	Hepatocellular Carcinoma	China	236	Postoperative NLR-PLR (0/1/2)<br><br>NLR> 2.3 and PLR>116 score 2, either 1 score 1, none score 0	Antiviral drug (entecavir or lamivudine) were given to patients with positive HBV-DNA	N/A	41	N/A	Multivariate:<br>2.894 (1.992-4.2)<br>p<0.001	Multivariate:<br>Microvascular invasion, transfusion
29.	Arigami et al[39]<br><br>2015	Retrospective	Oesophageal Squamous Cell Carcinoma	Japan	238	F-NLR (0-1/2)	Patients who have undergone neoadjuvant treatment were excluded	N/A	100	N/A	Multivariate:<br>1.94 (1.04-3.53)<br>p=0.037	Multivariate:<br>Depth of tumour invasion, lymph node metastasis
30.	Ha et al[25]<br><br>2016	Retrospective	Ampulla of Vater Cancer	South Korea	227	Systemic inflammatory index (≤780 vs. >780)	Adjuvant treatments including chemotherapy, radiotherapy and concurrent chemoradiotherapy	N/A	105	N/A	Multivariate:<br>0.924 (0.44-1.93)<br>p=0.833	Multivariate:<br>Vascular invasion, CA19-9.
31.	Li et al[34]<br><br>2016	Retrospective	Colorectal Cancer	China	5336	Albumin/ globulin ratio (<1.50 vs. ≥1.50)	5-Fu based adjuvant chemotherapy for stage 2/3 patients	588	611	N/A	Multivariate:<br>0.646 (0.543-0.767)<br>p<0.001	Multivariate:<br>Age, T stage, N stage, differentiation, venous invasion, LMR, NLR

32.	Christina et al[35]<br><br>2016	Retrospective	Oral cancer	Austria	144	CRP/ Neutrophils (low/high)	All patients received neoadjuvant radiotherapy in combination with systemic cytotoxic therapy	N/A	60 (5-year survival)	N/A	Multivariate:<br>2.7 (0.68-10.75)<br>p=0.16	Multivariate:<br>Regression grade
33.	Wang et al[36]<br><br>2016	Retrospective	Ovarian Cancer	China	143	Prognostic Inflammation Score (0/1/2)<br><br>involving NLR and serum albumin	No mention of adjuvant treatment	N/A	51	N/A	Multivariate:<br>PIS 1: 0.33 (0.16-0.67) p=0.002<br><br>PIS 2: 0.18 (0.09-0.38) p<0.001	Multivariate:<br>Metastasis, prognostic inflammation score
34.	Toyokawa et al[37]<br><br>2016	Retrospective	Thoracic Oesophageal Squamous Cell Carcinoma	Japan	185	CONUT score (≥3, ≤2) involving serum albumin concentration, total lymphocyte count, total cholesterol concentration	46 patients received neoadjuvant treatment (39 chemotherapy, 6 chemoradiotherapy, 1 radiotherapy)	N/A	77	N/A	Multivariate:<br>2.303 (1.191-4.455)<br>p=0.013	Multivariate:<br>Sex, performance status, ASA, cTNM stage
35.	Fu et al[110]<br><br>2016	Retrospective	Hepatocellular Carcinoma	China	Training: 772<br><br>Validation: 349	Inflammation-based score (IBS)	No mention of adjuvant treatment	N/A	377 (4-year survival)	N/A	Multivariate:<br>Training 4.247 (2.786-6.473) p<0.001	Multivariate:<br>GGT, mGPS, tumour number, microscopic vascular invasion, BCLC.

References:

1 Organization, W. H. *World Health Organization Cancer Fact Sheet*, <http://www.who.int/mediacentre/factsheets/fs297/en/> (2017).

2 Roxburgh, C. S. & McMillan, D. C. Role of systemic inflammatory response in predicting survival in patients with primary operable cancer. *Future oncology (London, England)* **6**, 149-163, doi:10.2217/fon.09.136 (2010).

3 Proctor, M. J. *et al.* A comparison of inflammation-based prognostic scores in patients with cancer. A Glasgow Inflammation Outcome Study. *European journal of cancer (Oxford, England : 1990)* **47**, 2633-2641, doi:10.1016/j.ejca.2011.03.028 (2011).

4 Bosanquet, N. & Sikora, K. The economics of cancer care in the UK. *The Lancet. Oncology* **5**, 568-574, doi:10.1016/s1470-2045(04)01569-4 (2004).

5 Coussens, L. M. & Werb, Z. Inflammation and cancer. *Nature* **420**, 860-867, doi:10.1038/nature01322 (2002).

6 Mantovani, A., Romero, P., Palucka, A. K. & Marincola, F. M. Tumour immunity: effector response to tumour and role of the microenvironment. *Lancet (London, England)* **371**, 771-783, doi:10.1016/s0140-6736(08)60241-x (2008).

7 Park, J. H., Watt, D. G., Roxburgh, C. S., Horgan, P. G. & McMillan, D. C. Colorectal Cancer, Systemic Inflammation, and Outcome: Staging the Tumor and Staging the Host. *Annals of surgery* **263**, 326-336, doi:10.1097/sla.0000000000001122 (2016).

8 McMillan, D. C., Crozier, J. E., Canna, K., Angerson, W. J. & McArdle, C. S. Evaluation of an inflammation-based prognostic score (GPS) in patients undergoing resection for colon and rectal cancer. *Int J Colorectal Dis* **22**, 881-886, doi:10.1007/s00384-006-0259-6 (2007).

9 Guthrie, G. J. *et al.* The systemic inflammation-based neutrophil-lymphocyte ratio: experience in patients with cancer. *Critical reviews in oncology/hematology* **88**, 218-230, doi:10.1016/j.critrevonc.2013.03.010 (2013).

10 Templeton, A. J. *et al.* Prognostic role of neutrophil-to-lymphocyte ratio in solid tumors: a systematic review and meta-analysis. *Journal of the National Cancer Institute* **106**, dju124, doi:10.1093/jnci/dju124 (2014).

11 McMillan, D. C. The systemic inflammation-based Glasgow Prognostic Score: a decade of experience in patients with cancer. *Cancer treatment reviews* **39**, 534-540, doi:10.1016/j.ctrv.2012.08.003 (2013).

12 Dolan, R. D., McSorley, S. T., Horgan, P. G., Laird, B. & McMillan, D. C. The role of the systemic inflammatory response in predicting outcomes in patients with advanced inoperable cancer: Systematic review and meta-analysis. *Critical Reviews in Oncology/Hematology*, doi:https://doi.org/10.1016/j.critrevonc.2017.06.002.

13 Higgins, J. G., S. Vol. 1 (ed J. Green Higgins, S.) 675 (John Wiley & Sons Ltd, London 2011).

14 Ishizuka, M., Nagata, H., Takagi, K., Iwasaki, Y. & Kubota, K. Combination of platelet count and neutrophil to lymphocyte ratio is a useful predictor of postoperative survival in patients with colorectal cancer. *British journal of cancer* **109**, 401-407, doi:10.1038/bjc.2013.350 (2013).

15 Ishizuka, M., Oyama, Y., Abe, A. & Kubota, K. Combination of platelet count and neutrophil to lymphocyte ratio is a useful predictor of postoperative survival in patients undergoing surgery for gastric cancer. *Journal of surgical oncology* **110**, 935-941, doi:10.1002/jso.23753 (2014).

16 Zhang, H. *et al.* Prognostic Significance of Combination of Preoperative Platelet Count and Neutrophil-Lymphocyte Ratio (COP-NLR) in Patients with Non-Small Cell Lung Cancer: Based on a Large Cohort Study. *PloS one* **10**, e0126496, doi:10.1371/journal.pone.0126496 (2015).

17 Neal, C. P. *et al.* Prognostic performance of inflammation-based prognostic indices in patients with resectable colorectal liver metastases. *Med Oncol* **32**, 144, doi:10.1007/s12032-015-0590-2 (2015).
18 Feng, J. F., Huang, Y. & Liu, J. S. Combination of neutrophil lymphocyte ratio and platelet lymphocyte ratio is a useful predictor of postoperative survival in patients with esophageal squamous cell carcinoma. *Onco Targets Ther* **6**, 1605-1612, doi:10.2147/ott.s52501 (2013).
19 Cummings, M. *et al.* Preoperative neutrophil:lymphocyte and platelet:lymphocyte ratios predict endometrial cancer survival. *Br J Cancer* **113**, 311-320, doi:10.1038/bjc.2015.200 (2015).
20 Li, C. *et al.* Postoperative neutrophil-to-lymphocyte ratio plus platelet-to-lymphocyte ratio predicts the outcomes of hepatocellular carcinoma. *J Surg Res* **198**, 73-79, doi:10.1016/j.jss.2015.05.003 (2015).
21 Ishizuka, M. *et al.* Clinical Significance of the C-Reactive Protein to Albumin Ratio for Survival After Surgery for Colorectal Cancer. *Annals of surgical oncology* **23**, 900-907, doi:10.1245/s10434-015-4948-7 (2016).
22 Xu, X. L., Yu, H. Q., Hu, W., Song, Q. & Mao, W. M. A Novel Inflammation-Based Prognostic Score, the C-Reactive Protein/Albumin Ratio Predicts the Prognosis of Patients with Operable Esophageal Squamous Cell Carcinoma. *PloS one* **10**, e0138657, doi:10.1371/journal.pone.0138657 (2015).
23 Ubukata, H. *et al.* Evaluations of interferon-gamma/interleukin-4 ratio and neutrophil/lymphocyte ratio as prognostic indicators in gastric cancer patients. *J Surg Oncol* **102**, 742-747, doi:10.1002/jso.21725 (2010).
24 Miyata, H. *et al.* Prognostic value of an inflammation-based score in patients undergoing pre-operative chemotherapy followed by surgery for esophageal cancer. *Exp Ther Med* **2**, 879-885, doi:10.3892/etm.2011.308 (2011).
25 Ha, H. R. *et al.* Survival Outcomes According to Adjuvant Treatment and Prognostic Factors Including Host Immune Markers in Patients with Curatively Resected Ampulla of Vater Cancer. *PloS one* **11**, e0151406, doi:10.1371/journal.pone.0151406 (2016).
26 Ilie, M. *et al.* Predictive clinical outcome of the intratumoral CD66b-positive neutrophil-to-CD8-positive T-cell ratio in patients with resectable nonsmall cell lung cancer. *Cancer* **118**, 1726-1737, doi:10.1002/cncr.26456 (2012).
27 Tomita, M., Shimizu, T., Ayabe, T., Nakamura, K. & Onitsuka, T. Elevated preoperative inflammatory markers based on neutrophil-to-lymphocyte ratio and C-reactive protein predict poor survival in resected non-small cell lung cancer. *Anticancer research* **32**, 3535-3538 (2012).
28 Chen, X. L. *et al.* Prognostic significance of the combination of preoperative hemoglobin, albumin, lymphocyte and platelet in patients with gastric carcinoma: a retrospective cohort study. *Oncotarget* **6**, 41370-41382, doi:10.18632/oncotarget.5629 (2015).
29 Sung, H. H. *et al.* Clinical significance of prognosis using the neutrophil-lymphocyte ratio and erythrocyte sedimentation rate in patients undergoing radical nephroureterectomy for upper urinary tract urothelial carcinoma. *BJU international* **115**, 587-594, doi:10.1111/bju.12846 (2015).
30 East, J. M. *et al.* Ratios derived from an array of standard haematological indices predict the oncological outcome in colon cancer. *Colorectal Dis* **16**, 442-449, doi:10.1111/codi.12561 (2014).
31 Shen, S. L. *et al.* Preoperative aspartate aminotransferase to platelet ratio is an independent prognostic factor for hepatitis B-induced hepatocellular carcinoma after hepatic resection. *Ann Surg Oncol* **21**, 3802-3809, doi:10.1245/s10434-014-3771-x (2014).

32 Aurello, P. *et al.* Value of preoperative inflammation-based prognostic scores in predicting overall survival and disease-free survival in patients with gastric cancer. *Annals of surgical oncology* **21**, 1998-2004, doi:10.1245/s10434-014-3533-9 (2014).

33 Sun, K. Y. *et al.* Novel immunological and nutritional-based prognostic index for gastric cancer. *World journal of gastroenterology* **21**, 5961-5971, doi:10.3748/wjg.v21.i19.5961 (2015).

34 Li, Y. *et al.* Nomograms for predicting prognostic value of inflammatory biomarkers in colorectal cancer patients after radical resection. *International journal of cancer* **139**, 220-231, doi:10.1002/ijc.30071 (2016).

35 Christina, E. C., Cornelia, C. & Edgar, S. Neoadjuvant radiotherapy plus radical surgery for locally advanced stage III/IV oral cancer: Analysis of prognostic factors affecting overall survival. *Oral oncology* **60**, 1-7, doi:10.1016/j.oraloncology.2016.06.014 (2016).

36 Wang, Y. Q. *et al.* A novel prognostic inflammation score predicts outcomes in patients with ovarian cancer. *Clinica chimica acta; international journal of clinical chemistry* **456**, 163-169, doi:10.1016/j.cca.2016.03.013 (2016).

37 Toyokawa, T. *et al.* The pretreatment Controlling Nutritional Status (CONUT) score is an independent prognostic factor in patients with resectable thoracic esophageal squamous cell carcinoma: results from a retrospective study. *BMC Cancer* **16**, 722, doi:10.1186/s12885-016-2696-0 (2016).

38 Nakamura, M. *et al.* New prognostic score for the survival of patients with esophageal squamous cell carcinoma. *Surg Today* **44**, 875-883, doi:10.1007/s00595-013-0628-z (2014).

39 Arigami, T. *et al.* Analysis of the Fibrinogen and Neutrophil-Lymphocyte Ratio in Esophageal Squamous Cell Carcinoma: A Promising Blood Marker of Tumor Progression and Prognosis. *Medicine (Baltimore)* **94**, e1702, doi:10.1097/md.0000000000001702 (2015).

40 Hirashima, K. *et al.* Prognostic significance of the modified Glasgow prognostic score in elderly patients with gastric cancer. *Journal of gastroenterology* **49**, 1040-1046, doi:10.1007/s00535-013-0855-5 (2014).

41 Kawashima, M. *et al.* Significance of the Glasgow Prognostic Score as a prognostic indicator for lung cancer surgery. *Interactive cardiovascular and thoracic surgery* **21**, 637-643, doi:10.1093/icvts/ivv223 (2015).

42 Takeno, S. *et al.* The high-sensitivity modified Glasgow prognostic score is superior to the modified Glasgow prognostic score as a prognostic predictor in patients with resectable gastric cancer. *Oncology* **87**, 205-214, doi:10.1159/000362601 (2014).

43 Ishizuka, M. *et al.* Usefulness of a modified inflammation-based prognostic system for predicting postoperative mortality of patients undergoing surgery for primary hepatocellular carcinoma. *J Surg Oncol* **103**, 801-806, doi:10.1002/jso.21857 (2011).

44 Bain, B., Seed, M. & Godsland, I. Normal values for peripheral blood white cell counts in women of four different ethnic origins. *Journal of clinical pathology* **37**, 188-193 (1984).

45 Bain, B. J. Ethnic and sex differences in the total and differential white cell count and platelet count. *Journal of clinical pathology* **49**, 664-666 (1996).

46 Azab, B., Camacho-Rivera, M. & Taioli, E. Average values and racial differences of neutrophil lymphocyte ratio among a nationally representative sample of United States subjects. *PloS one* **9**, e112361, doi:10.1371/journal.pone.0112361 (2014).

47 Guthrie, G. J., Roxburgh, C. S., Farhan-Alanie, O. M., Horgan, P. G. & McMillan, D. C. Comparison of the prognostic value of longitudinal measurements of systemic inflammation in patients undergoing curative resection of colorectal cancer. *British journal of cancer* **109**, 24-28, doi:10.1038/bjc.2013.330 (2013).

48 Wang, D. S. *et al.* Comparison of the prognostic values of various inflammation based factors in patients with pancreatic cancer. *Medical oncology (Northwood, London, England)* **29**, 3092-3100, doi:10.1007/s12032-012-0226-8 (2012).
49 Okuno, M. *et al.* Evaluation of inflammation-based prognostic scores in patients undergoing hepatobiliary resection for perihilar cholangiocarcinoma. *Journal of gastroenterology* **51**, 153-161, doi:10.1007/s00535-015-1103-y (2016).
50 Ishizuka, M., Nagata, H., Takagi, K., Horie, T. & Kubota, K. Inflammation-based prognostic score is a novel predictor of postoperative outcome in patients with colorectal cancer. *Annals of surgery* **246**, 1047-1051, doi:10.1097/SLA.0b013e3181454171 (2007).
51 Leitch, E. F. *et al.* Comparison of the prognostic value of selected markers of the systemic inflammatory response in patients with colorectal cancer. *British journal of cancer* **97**, 1266-1270, doi:10.1038/sj.bjc.6604027 (2007).
52 Kobayashi, T. *et al.* Inflammation-based prognostic score, prior to neoadjuvant chemoradiotherapy, predicts postoperative outcome in patients with esophageal squamous cell carcinoma. *Surgery* **144**, 729-735, doi:10.1016/j.surg.2008.08.015 (2008).
53 Roxburgh, C. S., Salmond, J. M., Horgan, P. G., Oien, K. A. & McMillan, D. C. Comparison of the prognostic value of inflammation-based pathologic and biochemical criteria in patients undergoing potentially curative resection for colorectal cancer. *Annals of surgery* **249**, 788-793, doi:10.1097/SLA.0b013e3181a3e738 (2009).
54 Ishizuka, M. *et al.* Systemic inflammatory response predicts postoperative outcome in patients with liver metastases from colorectal cancer. *J Surg Oncol* **100**, 38-42, doi:10.1002/jso.21294 (2009).
55 Crozier, J. E. *et al.* Relationship between emergency presentation, systemic inflammatory response, and cancer-specific survival in patients undergoing potentially curative surgery for colon cancer. *American journal of surgery* **197**, 544-549, doi:10.1016/j.amjsurg.2007.12.052 (2009).
56 Roxburgh, C. S., Wallace, A. M., Guthrie, G. K., Horgan, P. G. & McMillan, D. C. Comparison of the prognostic value of tumour- and patient-related factors in patients undergoing potentially curative surgery for colon cancer. *Colorectal Dis* **12**, 987-994, doi:10.1111/j.1463-1318.2009.01961.x (2010).
57 Richards, C. H. *et al.* The relationship between patient physiology, the systemic inflammatory response and survival in patients undergoing curative resection of colorectal cancer. *British journal of cancer* **103**, 1356-1361, doi:10.1038/sj.bjc.6605919 (2010).
58 Hefler-Frischmuth, K. *et al.* The inflammation-based modified Glasgow Prognostic Score in patients with vulvar cancer. *Eur J Obstet Gynecol Reprod Biol* **149**, 102-105, doi:10.1016/j.ejogrb.2009.12.027 (2010).
59 Kobayashi, T. *et al.* Inflammation-based prognostic score and number of lymph node metastases are independent prognostic factors in esophageal squamous cell carcinoma. *Dig Surg* **27**, 232-237, doi:10.1159/000276910 (2010).
60 Kobayashi, T. *et al.* Elevated C-reactive protein and hypoalbuminemia measured before resection of colorectal liver metastases predict postoperative survival. *Dig Surg* **27**, 285-290, doi:10.1159/000280021 (2010).
61 Moug, S. J. *et al.* Comparison of positive lymph node ratio with an inflammation-based prognostic score in colorectal cancer. *Br J Surg* **98**, 282-286, doi:10.1002/bjs.7294 (2011).
62 Dutta, S., Crumley, A. B., Fullarton, G. M., Horgan, P. G. & McMillan, D. C. Comparison of the prognostic value of tumour- and patient-related factors in patients undergoing potentially

curative resection of oesophageal cancer. *World J Surg* **35**, 1861-1866, doi:10.1007/s00268-011-1130-7 (2011).

63 Dutta, S., Al-Mrabt, N. M., Fullarton, G. M., Horgan, P. G. & McMillan, D. C. A comparison of POSSUM and GPS models in the prediction of post-operative outcome in patients undergoing oesophago-gastric cancer resection. *Annals of surgical oncology* **18**, 2808-2817, doi:10.1245/s10434-011-1676-5 (2011).

64 Crumley, A. B. *et al.* Interrelationships between tumor proliferative activity, leucocyte and macrophage infiltration, systemic inflammatory response, and survival in patients selected for potentially curative resection for gastroesophageal cancer. *Annals of surgical oncology* **18**, 2604-2612, doi:10.1245/s10434-011-1658-7 (2011).

65 Jamieson, N. B. *et al.* A prospective comparison of the prognostic value of tumor- and patient-related factors in patients undergoing potentially curative surgery for pancreatic ductal adenocarcinoma. *Annals of surgical oncology* **18**, 2318-2328, doi:10.1245/s10434-011-1560-3 (2011).

66 Roxburgh, C. S. *et al.* Relationship between preoperative comorbidity, systemic inflammatory response, and survival in patients undergoing curative resection for colorectal cancer. *Annals of surgical oncology* **18**, 997-1005, doi:10.1245/s10434-010-1410-8 (2011).

67 Vashist, Y. K. *et al.* Glasgow Prognostic Score is a predictor of perioperative and long-term outcome in patients with only surgically treated esophageal cancer. *Annals of surgical oncology* **18**, 1130-1138, doi:10.1245/s10434-010-1383-7 (2011).

68 Nozoe, T. *et al.* Significance of modified Glasgow prognostic score as a useful indicator for prognosis of patients with gastric carcinoma. *American journal of surgery* **201**, 186-191, doi:10.1016/j.amjsurg.2010.01.030 (2011).

69 Roxburgh, C. *et al.* Adjuvant chemotherapy for resected colon cancer: comparison of the prognostic value of tumour and patient related factors. *Int J Colorectal Dis* **26**, 483-492, doi:10.1007/s00384-010-1120-5 (2011).

70 Dutta, S. *et al.* The relationship between tumour necrosis, tumour proliferation, local and systemic inflammation, microvessel density and survival in patients undergoing potentially curative resection of oesophageal adenocarcinoma. *British journal of cancer* **106**, 702-710, doi:10.1038/bjc.2011.610 (2012).

71 Ishizuka, M. *et al.* Impact of an inflammation-based prognostic system on patients undergoing surgery for hepatocellular carcinoma: a retrospective study of 398 Japanese patients. *American journal of surgery* **203**, 101-106, doi:10.1016/j.amjsurg.2010.09.030 (2012).

72 Richards, C. H. *et al.* Prognostic value of tumour necrosis and host inflammatory responses in colorectal cancer. *Br J Surg* **99**, 287-294, doi:10.1002/bjs.7755 (2012).

73 Qayyum, T. *et al.* Prospective study of the role of inflammation in renal cancer. *Urol Int* **88**, 277-281, doi:10.1159/000334971 (2012).

74 Sugimoto, K. *et al.* Glasgow prognostic score as a prognostic factor in patients undergoing curative surgery for colorectal cancer. *Dig Surg* **29**, 503-509, doi:10.1159/000346002 (2012).

75 Kubota, T. *et al.* Significance of the inflammation-based Glasgow prognostic score for short- and long-term outcomes after curative resection of gastric cancer. *J Gastrointest Surg* **16**, 2037-2044, doi:10.1007/s11605-012-2036-x (2012).

76 Powell, A. G. *et al.* The relationship between tumour site, clinicopathological characteristics and cancer-specific survival in patients undergoing surgery for colorectal cancer. *Colorectal Dis* **14**, 1493-1499, doi:10.1111/j.1463-1318.2012.03048.x (2012).

77 La Torre, M. *et al.* The glasgow prognostic score as a predictor of survival in patients with potentially resectable pancreatic adenocarcinoma. *Annals of surgical oncology* **19**, 2917-2923, doi:10.1245/s10434-012-2348-9 (2012).

78 Dutta, S., Crumley, A. B., Fullarton, G. M., Horgan, P. G. & McMillan, D. C. Comparison of the prognostic value of tumour and patient related factors in patients undergoing potentially curative resection of gastric cancer. *American journal of surgery* **204**, 294-299, doi:10.1016/j.amjsurg.2011.10.015 (2012).

79 Wang, D. S. *et al.* Comparison of the prognostic value of various preoperative inflammation-based factors in patients with stage III gastric cancer. *Tumour biology : the journal of the International Society for Oncodevelopmental Biology and Medicine* **33**, 749-756, doi:10.1007/s13277-011-0285-z (2012).

80 Lamb, G. W., Aitchison, M., Ramsey, S., Housley, S. L. & McMillan, D. C. Clinical utility of the Glasgow Prognostic Score in patients undergoing curative nephrectomy for renal clear cell cancer: basis of new prognostic scoring systems. *British journal of cancer* **106**, 279-283, doi:10.1038/bjc.2011.556 (2012).

81 Ishizuka, M., Nagata, H., Takagi, K., Iwasaki, Y. & Kubota, K. Inflammation-based prognostic system predicts postoperative survival of colorectal cancer patients with a normal preoperative serum level of carcinoembryonic antigen. *Annals of surgical oncology* **19**, 3422-3431, doi:10.1245/s10434-012-2384-5 (2012).

82 Jiang, X. *et al.* Prognostic importance of the inflammation-based Glasgow prognostic score in patients with gastric cancer. *British journal of cancer* **107**, 275-279, doi:10.1038/bjc.2012.262 (2012).

83 Jamieson, N. B. *et al.* The relationship between tumor inflammatory cell infiltrate and outcome in patients with pancreatic ductal adenocarcinoma. *Annals of surgical oncology* **19**, 3581-3590, doi:10.1245/s10434-012-2370-y (2012).

84 Stotz, M. *et al.* Increased neutrophil-lymphocyte ratio is a poor prognostic factor in patients with primary operable and inoperable pancreatic cancer. *British journal of cancer* **109**, 416-421, doi:10.1038/bjc.2013.332 (2013).

85 Shiba, H. *et al.* Glasgow prognostic score predicts therapeutic outcome after pancreaticoduodenectomy for carcinoma of the ampulla of vater. *Anticancer research* **33**, 2715-2721 (2013).

86 Oshiro, Y. *et al.* Inflammation-based prognostic score is a useful predictor of postoperative outcome in patients with extrahepatic cholangiocarcinoma. *J Hepatobiliary Pancreat Sci* **20**, 389-395, doi:10.1007/s00534-012-0550-6 (2013).

87 Horino, K. *et al.* Glasgow Prognostic Score as a useful prognostic factor after hepatectomy for hepatocellular carcinoma. *Int J Clin Oncol* **18**, 829-838, doi:10.1007/s10147-012-0451-3 (2013).

88 Ishizuka, M., Nagata, H., Takagi, K., Iwasaki, Y. & Kubota, K. Inflammation-based prognostic system predicts survival after surgery for stage IV colorectal cancer. *American journal of surgery* **205**, 22-28, doi:10.1016/j.amjsurg.2012.04.012 (2013).

89 Son, H. J. *et al.* Preoperative plasma hyperfibrinogenemia is predictive of poor prognosis in patients with nonmetastatic colon cancer. *Annals of surgical oncology* **20**, 2908-2913, doi:10.1245/s10434-013-2968-8 (2013).

90 Nozoe, T., Matono, R., Ijichi, H., Ohga, T. & Ezaki, T. Glasgow Prognostic Score (GPS) can be a useful indicator to determine prognosis of patients with colorectal carcinoma. *Int Surg* **99**, 512-517, doi:10.9738/intsurg-d-13-00118.1 (2014).

91 Pinato, D. J. *et al.* Prognostic performance of inflammation-based prognostic indices in primary operable non-small cell lung cancer. *British journal of cancer* **110**, 1930-1935, doi:10.1038/bjc.2014.145 (2014).

92 Huang, J. *et al.* The inflammation-based scores to predict prognosis of patients with hepatocellular carcinoma after hepatectomy. *Medical oncology (Northwood, London, England)* **31**, 883, doi:10.1007/s12032-014-0883-x (2014).

93 Feng, J. F., Zhao, Q. & Chen, Q. X. Prognostic significance of Glasgow prognostic score in patients undergoing esophagectomy for esophageal squamous cell carcinoma. *Saudi J Gastroenterol* **20**, 48-53, doi:10.4103/1319-3767.126319 (2014).

94 Forrest, R. *et al.* Comparison of visual and automated assessment of tumour inflammatory infiltrates in patients with colorectal cancer. *European journal of cancer (Oxford, England : 1990)* **50**, 544-552, doi:10.1016/j.ejca.2013.11.003 (2014).

95 Wu, X. S. *et al.* Evaluation of two inflammation-based prognostic scores in patients with resectable gallbladder carcinoma. *Annals of surgical oncology* **21**, 449-457, doi:10.1245/s10434-013-3292-z (2014).

96 Sun, Z. Q. *et al.* Prognostic significance of preoperative fibrinogen in patients with colon cancer. *World J Gastroenterol* **20**, 8583-8591, doi:10.3748/wjg.v20.i26.8583 (2014).

97 Nakagawa, K. *et al.* The modified Glasgow prognostic score as a predictor of survival after hepatectomy for colorectal liver metastases. *Annals of surgical oncology* **21**, 1711-1718, doi:10.1245/s10434-013-3342-6 (2014).

98 Miyazaki, T. *et al.* Inflammation-based scoring is a useful prognostic predictor of pulmonary resection for elderly patients with clinical stage I non-small-cell lung cancer. *Eur J Cardiothorac Surg* **47**, e140-145, doi:10.1093/ejcts/ezu514 (2015).

99 Matsuda, S. *et al.* Cumulative prognostic scores based on plasma fibrinogen and serum albumin levels in esophageal cancer patients treated with transthoracic esophagectomy: comparison with the Glasgow prognostic score. *Annals of surgical oncology* **22**, 302-310, doi:10.1245/s10434-014-3857-5 (2015).

100 Farhan-Alanie, O. M., McMahon, J. & McMillan, D. C. Systemic inflammatory response and survival in patients undergoing curative resection of oral squamous cell carcinoma. *Br J Oral Maxillofac Surg* **53**, 126-131, doi:10.1016/j.bjoms.2014.10.007 (2015).

101 Ferro, M. *et al.* Modified Glasgow Prognostic Score is Associated With Risk of Recurrence in Bladder Cancer Patients After Radical Cystectomy: A Multicenter Experience. *Medicine (Baltimore)* **94**, e1861, doi:10.1097/md.0000000000001861 (2015).

102 Ni, X. C. *et al.* Prognostic Value of the Modified Glasgow Prognostic Score in Patients Undergoing Radical Surgery for Hepatocellular Carcinoma. *Medicine (Baltimore)* **94**, e1486, doi:10.1097/md.0000000000001486 (2015).

103 Hirahara, N. *et al.* Impact of inflammation-based prognostic score on survival after curative thoracoscopic esophagectomy for esophageal cancer. *Eur J Surg Oncol* **41**, 1308-1315, doi:10.1016/j.ejso.2015.07.008 (2015).

104 Shibutani, M. *et al.* The prognostic significance of a postoperative systemic inflammatory response in patients with colorectal cancer. *World J Surg Oncol* **13**, 194, doi:10.1186/s12957-015-0609-3 (2015).

105 Shiba, H. *et al.* Glasgow prognostic score predicts outcome after surgical resection of gallbladder cancer. *World J Surg* **39**, 753-758, doi:10.1007/s00268-014-2844-0 (2015).

106 Watt, D. G., Martin, J. C., Park, J. H., Horgan, P. G. & McMillan, D. C. Neutrophil count is the most important prognostic component of the differential white cell count in patients

undergoing elective surgery for colorectal cancer. *American journal of surgery* **210**, 24-30, doi:10.1016/j.amjsurg.2014.12.031 (2015).

107 Okamura, Y. *et al.* Preoperative neutrophil to lymphocyte ratio and prognostic nutritional index predict overall survival after hepatectomy for hepatocellular carcinoma. *World J Surg* **39**, 1501-1509, doi:10.1007/s00268-015-2982-z (2015).

108 Abe, T. *et al.* Prediction of long-term survival by using the Glasgow Prognostic Score in patients with hepatocellular carcinoma after liver transplantation. *Hepatol Res* **46**, 622-633, doi:10.1111/hepr.12597 (2016).

109 Park, J. H. *et al.* Mismatch repair status in patients with primary operable colorectal cancer: associations with the local and systemic tumour environment. *British journal of cancer* **114**, 562-570, doi:10.1038/bjc.2016.17 (2016).

110 Fu, Y. P. *et al.* A Novel and Validated Inflammation-Based Score (IBS) Predicts Survival in Patients With Hepatocellular Carcinoma Following Curative Surgical Resection: A STROBE-Compliant Article. *Medicine (Baltimore)* **95**, e2784, doi:10.1097/md.0000000000002784 (2016).

111 Fan, H. *et al.* Comparison of the Glasgow Prognostic Score (GPS) and the modified Glasgow Prognostic Score (mGPS) in evaluating the prognosis of patients with operable and inoperable non-small cell lung cancer. *J Cancer Res Clin Oncol* **142**, 1285-1297, doi:10.1007/s00432-015-2113-0 (2016).

112 Chan, J. C. *et al.* The Lymphocyte-to-Monocyte Ratio is a Superior Predictor of Overall Survival in Comparison to Established Biomarkers of Resectable Colorectal Cancer. *Annals of surgery*, doi:10.1097/sla.0000000000001743 (2016).

113 Walsh, S. M., Casey, S., Kennedy, R., Ravi, N. & Reynolds, J. V. Does the modified Glasgow Prognostic Score (mGPS) have a prognostic role in esophageal cancer? *J Surg Oncol* **113**, 732-737, doi:10.1002/jso.24225 (2016).

114 Otowa, Y. *et al.* Changes in modified Glasgow prognostic score after neoadjuvant chemotherapy is a prognostic factor in clinical stage II/III esophageal cancer. *Dis Esophagus* **29**, 146-151, doi:10.1111/dote.12316 (2016).

115 Melling, N. *et al.* Glasgow Prognostic Score may be a prognostic index for overall and perioperative survival in gastric cancer without perioperative treatment. *Surgery* **159**, 1548-1556, doi:10.1016/j.surg.2016.01.018 (2016).

116 Halazun, K. J. *et al.* Elevated preoperative neutrophil to lymphocyte ratio predicts survival following hepatic resection for colorectal liver metastases. *Eur J Surg Oncol* **34**, 55-60, doi:10.1016/j.ejso.2007.02.014 (2008).

117 Gomez, D., Morris-Stiff, G., Toogood, G. J., Lodge, J. P. & Prasad, K. R. Impact of systemic inflammation on outcome following resection for intrahepatic cholangiocarcinoma. *J Surg Oncol* **97**, 513-518, doi:10.1002/jso.21001 (2008).

118 Sarraf, K. M. *et al.* Neutrophil/lymphocyte ratio and its association with survival after complete resection in non-small cell lung cancer. *J Thorac Cardiovasc Surg* **137**, 425-428, doi:10.1016/j.jtcvs.2008.05.046 (2009).

119 Kishi, Y. *et al.* Blood neutrophil-to-lymphocyte ratio predicts survival in patients with colorectal liver metastases treated with systemic chemotherapy. *Annals of surgical oncology* **16**, 614-622, doi:10.1245/s10434-008-0267-6 (2009).

120 Cho, H. *et al.* Pre-treatment neutrophil to lymphocyte ratio is elevated in epithelial ovarian cancer and predicts survival after treatment. *Cancer Immunol Immunother* **58**, 15-23, doi:10.1007/s00262-008-0516-3 (2009).

121 Smith, R. A. *et al.* Preoperative platelet-lymphocyte ratio is an independent significant prognostic marker in resected pancreatic ductal adenocarcinoma. *American journal of surgery* **197**, 466-472, doi:10.1016/j.amjsurg.2007.12.057 (2009).
122 Halazun, K. J. *et al.* Negative impact of neutrophil-lymphocyte ratio on outcome after liver transplantation for hepatocellular carcinoma. *Annals of surgery* **250**, 141-151, doi:10.1097/SLA.0b013e3181a77e59 (2009).
123 Jagdev, S. P. *et al.* Improving the accuracy of pre-operative survival prediction in renal cell carcinoma with C-reactive protein. *British journal of cancer* **103**, 1649-1656, doi:10.1038/sj.bjc.6605973 (2010).
124 Shimada, H. *et al.* High preoperative neutrophil-lymphocyte ratio predicts poor survival in patients with gastric cancer. *Gastric Cancer* **13**, 170-176, doi:10.1007/s10120-010-0554-3 (2010).
125 Bhatti, I., Peacock, O., Lloyd, G., Larvin, M. & Hall, R. I. Preoperative hematologic markers as independent predictors of prognosis in resected pancreatic ductal adenocarcinoma: neutrophil-lymphocyte versus platelet-lymphocyte ratio. *American journal of surgery* **200**, 197-203, doi:10.1016/j.amjsurg.2009.08.041 (2010).
126 Mohri, Y. *et al.* Prognostic significance of host- and tumor-related factors in patients with gastric cancer. *World J Surg* **34**, 285-290, doi:10.1007/s00268-009-0302-1 (2010).
127 Liu, H. *et al.* The baseline ratio of neutrophils to lymphocytes is associated with patient prognosis in rectal carcinoma. *J Gastrointest Cancer* **41**, 116-120, doi:10.1007/s12029-009-9125-4 (2010).
128 Kao, S. C. *et al.* Low calretinin expression and high neutrophil-to-lymphocyte ratio are poor prognostic factors in patients with malignant mesothelioma undergoing extrapleural pneumonectomy. *J Thorac Oncol* **6**, 1923-1929, doi:10.1097/JTO.0b013e31822a3740 (2011).
129 Jung, M. R. *et al.* Elevated preoperative neutrophil to lymphocyte ratio predicts poor survival following resection in late stage gastric cancer. *J Surg Oncol* **104**, 504-510, doi:10.1002/jso.21986 (2011).
130 Sharaiha, R. Z. *et al.* Elevated preoperative neutrophil:lymphocyte ratio as a predictor of postoperative disease recurrence in esophageal cancer. *Annals of surgical oncology* **18**, 3362-3369, doi:10.1245/s10434-011-1754-8 (2011).
131 Tomita, M., Shimizu, T., Ayabe, T., Yonei, A. & Onitsuka, T. Preoperative neutrophil to lymphocyte ratio as a prognostic predictor after curative resection for non-small cell lung cancer. *Anticancer research* **31**, 2995-2998 (2011).
132 Hung, H. Y. *et al.* Effect of preoperative neutrophil-lymphocyte ratio on the surgical outcomes of stage II colon cancer patients who do not receive adjuvant chemotherapy. *Int J Colorectal Dis* **26**, 1059-1065, doi:10.1007/s00384-011-1192-x (2011).
133 Neal, C. P. *et al.* Preoperative systemic inflammation and infectious complications after resection of colorectal liver metastases. *Arch Surg* **146**, 471-478, doi:10.1001/archsurg.2011.50 (2011).
134 Asher, V., Lee, J., Innamaa, A. & Bali, A. Preoperative platelet lymphocyte ratio as an independent prognostic marker in ovarian cancer. *Clin Transl Oncol* **13**, 499-503, doi:10.1007/s12094-011-0687-9 (2011).
135 Wang, G. Y. *et al.* A scoring model based on neutrophil to lymphocyte ratio predicts recurrence of HBV-associated hepatocellular carcinoma after liver transplantation. *PloS one* **6**, e25295, doi:10.1371/journal.pone.0025295 (2011).

136 Bertuzzo, V. R. *et al.* Analysis of factors affecting recurrence of hepatocellular carcinoma after liver transplantation with a special focus on inflammation markers. *Transplantation* **91**, 1279-1285, doi:10.1097/TP.0b013e3182187cf0 (2011).

137 Idowu, O. K., Ding, Q., Taktak, A. F., Chandrasekar, C. R. & Yin, Q. Clinical implication of pretreatment neutrophil to lymphocyte ratio in soft tissue sarcoma. *Biomarkers* **17**, 539-544, doi:10.3109/1354750x.2012.699554 (2012).

138 Ishizuka, M. *et al.* Clinical significance of tumor pathology for postoperative survival of patients undergoing surgery for stage IV colorectal cancer. *Anticancer research* **32**, 3291-3297 (2012).

139 Gondo, T. *et al.* Prognostic value of neutrophil-to-lymphocyte ratio and establishment of novel preoperative risk stratification model in bladder cancer patients treated with radical cystectomy. *Urology* **79**, 1085-1091, doi:10.1016/j.urology.2011.11.070 (2012).

140 Kwon, H. C. *et al.* Clinical significance of preoperative neutrophil-lymphocyte versus platelet-lymphocyte ratio in patients with operable colorectal cancer. *Biomarkers* **17**, 216-222, doi:10.3109/1354750x.2012.656705 (2012).

141 Carruthers, R. *et al.* Systemic inflammatory response is a predictor of outcome in patients undergoing preoperative chemoradiation for locally advanced rectal cancer. *Colorectal Dis* **14**, e701-707, doi:10.1111/j.1463-1318.2012.03147.x (2012).

142 Wang, L. *et al.* Esophageal carcinosarcoma: a unique entity with better prognosis. *Annals of surgical oncology* **20**, 997-1004, doi:10.1245/s10434-012-2658-y (2013).

143 Choi, E. S., Kim, H. S. & Han, I. Elevated preoperative systemic inflammatory markers predict poor outcome in localized soft tissue sarcoma. *Annals of surgical oncology* **21**, 778-785, doi:10.1245/s10434-013-3418-3 (2014).

144 Szkandera, J. *et al.* Elevated preoperative neutrophil/lymphocyte ratio is associated with poor prognosis in soft-tissue sarcoma patients. *British journal of cancer* **108**, 1677-1683, doi:10.1038/bjc.2013.135 (2013).

145 Krane, L. S. *et al.* Preoperative neutrophil/lymphocyte ratio predicts overall survival and extravesical disease in patients undergoing radical cystectomy. *Journal of endourology* **27**, 1046-1050, doi:10.1089/end.2012.0606 (2013).

146 Pichler, M. *et al.* Validation of the pre-treatment neutrophil-lymphocyte ratio as a prognostic factor in a large European cohort of renal cell carcinoma patients. *British journal of cancer* **108**, 901-907, doi:10.1038/bjc.2013.28 (2013).

147 Jankova, L., Dent, O. F., Chan, C., Chapuis, P. & Clarke, S. J. Preoperative neutrophil/lymphocyte ratio predicts overall survival but does not predict recurrence or cancer-specific survival after curative resection of node-positive colorectal cancer. *BMC Cancer* **13**, 442, doi:10.1186/1471-2407-13-442 (2013).

148 Fu, S. J. *et al.* Prognostic value of preoperative peripheral neutrophil-to-lymphocyte ratio in patients with HBV-associated hepatocellular carcinoma after radical hepatectomy. *Medical oncology (Northwood, London, England)* **30**, 721, doi:10.1007/s12032-013-0721-6 (2013).

149 Shibutani, M. *et al.* A high preoperative neutrophil-to-lymphocyte ratio is associated with poor survival in patients with colorectal cancer. *Anticancer research* **33**, 3291-3294 (2013).

150 Forget, P. *et al.* Neutrophil:lymphocyte ratio and intraoperative use of ketorolac or diclofenac are prognostic factors in different cohorts of patients undergoing breast, lung, and kidney cancer surgery. *Annals of surgical oncology* **20 Suppl 3**, S650-660, doi:10.1245/s10434-013-3136-x (2013).

151 Absenger, G. *et al.* A derived neutrophil to lymphocyte ratio predicts clinical outcome in stage II and III colon cancer patients. *British journal of cancer* **109**, 395-400, doi:10.1038/bjc.2013.346 (2013).
152 Mano, Y. *et al.* Preoperative neutrophil-to-lymphocyte ratio is a predictor of survival after hepatectomy for hepatocellular carcinoma: a retrospective analysis. *Annals of surgery* **258**, 301-305, doi:10.1097/SLA.0b013e318297ad6b (2013).
153 Azuma, T. *et al.* Preoperative neutrophil-lymphocyte ratio as an independent prognostic marker for patients with upper urinary tract urothelial carcinoma. *Clinical genitourinary cancer* **11**, 337-341, doi:10.1016/j.clgc.2013.04.003 (2013).
154 Dumitrascu, T., Chirita, D., Ionescu, M. & Popescu, I. Resection for hilar cholangiocarcinoma: analysis of prognostic factors and the impact of systemic inflammation on long-term outcome. *J Gastrointest Surg* **17**, 913-924, doi:10.1007/s11605-013-2144-2 (2013).
155 Perisanidis, C. *et al.* High neutrophil-to-lymphocyte ratio is an independent marker of poor disease-specific survival in patients with oral cancer. *Medical oncology (Northwood, London, England)* **30**, 334, doi:10.1007/s12032-012-0334-5 (2013).
156 Noh, H., Eomm, M. & Han, A. Usefulness of pretreatment neutrophil to lymphocyte ratio in predicting disease-specific survival in breast cancer patients. *J Breast Cancer* **16**, 55-59, doi:10.4048/jbc.2013.16.1.55 (2013).
157 Liao, Y., Ni, Y., He, R., Liu, W. & Du, J. Clinical implications of fibroblast activation protein-alpha in non-small cell lung cancer after curative resection: a new predictor for prognosis. *J Cancer Res Clin Oncol* **139**, 1523-1528, doi:10.1007/s00432-013-1471-8 (2013).
158 Bambury, R. M. *et al.* The association of pre-treatment neutrophil to lymphocyte ratio with overall survival in patients with glioblastoma multiforme. *J Neurooncol* **114**, 149-154, doi:10.1007/s11060-013-1164-9 (2013).
159 Toiyama, Y. *et al.* C-reactive protein as predictor of recurrence in patients with rectal cancer undergoing chemoradiotherapy followed by surgery. *Anticancer research* **33**, 5065-5074 (2013).
160 Szkandera, J. *et al.* The lymphocyte/monocyte ratio predicts poor clinical outcome and improves the predictive accuracy in patients with soft tissue sarcomas. *Int J Cancer* **135**, 362-370, doi:10.1002/ijc.28677 (2014).
161 Dalpiaz, O. *et al.* Validation of pretreatment neutrophil-lymphocyte ratio as a prognostic factor in a European cohort of patients with upper tract urothelial carcinoma. *BJU international* **114**, 334-339, doi:10.1111/bju.12441 (2014).
162 Luo, H. L. *et al.* Subclassification of upper urinary tract urothelial carcinoma by the neutrophil-to-lymphocyte ratio (NLR) improves prediction of oncological outcome. *BJU international* **113**, E144-149, doi:10.1111/bju.12582 (2014).
163 Zhang, T. *et al.* Evaluation of preoperative hematologic markers as prognostic factors and establishment of novel risk stratification in resected pN0 non-small-cell lung cancer. *PloS one* **9**, e111494, doi:10.1371/journal.pone.0111494 (2014).
164 Ying, H. Q. *et al.* The prognostic value of preoperative NLR, d-NLR, PLR and LMR for predicting clinical outcome in surgical colorectal cancer patients. *Medical oncology (Northwood, London, England)* **31**, 305, doi:10.1007/s12032-014-0305-0 (2014).
165 Linton, A. *et al.* Factors associated with survival in a large series of patients with malignant pleural mesothelioma in New South Wales. *British journal of cancer* **111**, 1860-1869, doi:10.1038/bjc.2014.478 (2014).

166 Kubo, T. *et al.* Impact of the perioperative neutrophil-to-lymphocyte ratio on the long-term survival following an elective resection of colorectal carcinoma. *International journal of colorectal disease* **29**, 1091-1099, doi:10.1007/s00384-014-1964-1 (2014).
167 Viers, B. R. *et al.* Preoperative neutrophil-lymphocyte ratio predicts death among patients with localized clear cell renal carcinoma undergoing nephrectomy. *Urologic oncology* **32**, 1277-1284, doi:10.1016/j.urolonc.2014.05.014 (2014).
168 Koh, Y. W., Lee, H. J., Ahn, J. H., Lee, J. W. & Gong, G. Prognostic significance of the ratio of absolute neutrophil to lymphocyte counts for breast cancer patients with ER/PR-positivity and HER2-negativity in neoadjuvant setting. *Tumour biology : the journal of the International Society for Oncodevelopmental Biology and Medicine* **35**, 9823-9830, doi:10.1007/s13277-014-2282-5 (2014).
169 Hermanns, T. *et al.* Pre-treatment neutrophil-to-lymphocyte ratio as predictor of adverse outcomes in patients undergoing radical cystectomy for urothelial carcinoma of the bladder. *British journal of cancer* **111**, 444-451, doi:10.1038/bjc.2014.305 (2014).
170 Tanaka, N. *et al.* A multi-institutional validation of the prognostic value of the neutrophil-to-lymphocyte ratio for upper tract urothelial carcinoma treated with radical nephroureterectomy. *Annals of surgical oncology* **21**, 4041-4048, doi:10.1245/s10434-014-3830-3 (2014).
171 Jiang, N. *et al.* The role of preoperative neutrophil-lymphocyte and platelet-lymphocyte ratio in patients after radical resection for gastric cancer. *Biomarkers : biochemical indicators of exposure, response, and susceptibility to chemicals* **19**, 444-451, doi:10.3109/1354750x.2014.926567 (2014).
172 Yuan, D. *et al.* The preoperative neutrophil-lymphocyte ratio predicts recurrence and survival among patients undergoing R0 resections of adenocarcinomas of the esophagogastric junction. *Journal of surgical oncology* **110**, 333-340, doi:10.1002/jso.23651 (2014).
173 Ozdemir, Y., Akin, M. L., Sucullu, I., Balta, A. Z. & Yucel, E. Pretreatment neutrophil/lymphocyte ratio as a prognostic aid in colorectal cancer. *Asian Pacific journal of cancer prevention : APJCP* **15**, 2647-2650 (2014).
174 Feng, J. F., Huang, Y. & Chen, Q. X. Preoperative platelet lymphocyte ratio (PLR) is superior to neutrophil lymphocyte ratio (NLR) as a predictive factor in patients with esophageal squamous cell carcinoma. *World journal of surgical oncology* **12**, 58, doi:10.1186/1477-7819-12-58 (2014).
175 Viers, B. R. *et al.* Pretreatment neutrophil-to-lymphocyte ratio is associated with advanced pathologic tumor stage and increased cancer-specific mortality among patients with urothelial carcinoma of the bladder undergoing radical cystectomy. *European urology* **66**, 1157-1164, doi:10.1016/j.eururo.2014.02.042 (2014).
176 McNamara, M. G. *et al.* Neutrophil/lymphocyte ratio as a prognostic factor in biliary tract cancer. *European journal of cancer (Oxford, England : 1990)* **50**, 1581-1589, doi:10.1016/j.ejca.2014.02.015 (2014).
177 Malietzis, G. *et al.* A preoperative neutrophil to lymphocyte ratio of 3 predicts disease-free survival after curative elective colorectal cancer surgery. *Annals of surgery* **260**, 287-292, doi:10.1097/sla.0000000000000216 (2014).
178 Grivas, N. *et al.* Clinico-pathological prognostic factors of renal cell carcinoma: A 15-year review from a single center in Greece. *Urology annals* **6**, 116-121, doi:10.4103/0974-7796.130552 (2014).

179 Shen, L. *et al.* Baseline neutrophil-lymphocyte ratio (>/=2.8) as a prognostic factor for patients with locally advanced rectal cancer undergoing neoadjuvant chemoradiation. *Radiation oncology (London, England)* **9**, 295, doi:10.1186/s13014-014-0295-2 (2014).

180 Neofytou, K. *et al.* Elevated platelet to lymphocyte ratio predicts poor prognosis after hepatectomy for liver-only colorectal metastases, and it is superior to neutrophil to lymphocyte ratio as an adverse prognostic factor. *Medical oncology (Northwood, London, England)* **31**, 239, doi:10.1007/s12032-014-0239-6 (2014).

181 Song, Y. *et al.* Preoperative neutrophil-to-lymphocyte ratio as prognostic predictor for hypopharyngeal squamous cell carcinoma after radical resections. *J Craniofac Surg* **26**, e137-140, doi:10.1097/scs.0000000000001235 (2015).

182 Takahashi, Y. *et al.* Prognostic Significance of Preoperative Neutrophil-Lymphocyte Ratios in Patients with Stage I Non-small Cell Lung Cancer After Complete Resection. *Annals of surgical oncology* **22 Suppl 3**, S1324-1331, doi:10.1245/s10434-015-4735-5 (2015).

183 Tu, X. P. *et al.* Preoperative neutrophil-to-lymphocyte ratio is an independent prognostic marker in patients with laryngeal squamous cell carcinoma. *BMC cancer* **15**, 743, doi:10.1186/s12885-015-1727-6 (2015).

184 Shin, J. S., Suh, K. W. & Oh, S. Y. Preoperative neutrophil to lymphocyte ratio predicts survival in patients with T1-2N0 colorectal cancer. *Journal of surgical oncology* **112**, 654-657, doi:10.1002/jso.24061 (2015).

185 Que, Y. *et al.* Preoperative platelet-lymphocyte ratio is superior to neutrophil-lymphocyte ratio as a prognostic factor for soft-tissue sarcoma. *BMC cancer* **15**, 648, doi:10.1186/s12885-015-1654-6 (2015).

186 Hsu, J. T. *et al.* Prognostic Value of the Preoperative Neutrophil to Lymphocyte Ratio in Resectable Gastric Cancer. *Medicine* **94**, e1589, doi:10.1097/md.0000000000001589 (2015).

187 Shimizu, K. *et al.* Preoperative neutrophil/lymphocyte ratio and prognostic nutritional index predict survival in patients with non-small cell lung cancer. *World journal of surgical oncology* **13**, 291, doi:10.1186/s12957-015-0710-7 (2015).

188 Han, S. *et al.* Pre-treatment neutrophil-to-lymphocyte ratio is associated with neutrophil and T-cell infiltration and predicts clinical outcome in patients with glioblastoma. *BMC cancer* **15**, 617, doi:10.1186/s12885-015-1629-7 (2015).

189 Liao, R. *et al.* Preoperative neutrophil-to-lymphocyte ratio predicts recurrence of patients with single-nodule small hepatocellular carcinoma following curative resection: a retrospective report. *World journal of surgical oncology* **13**, 265, doi:10.1186/s12957-015-0670-y (2015).

190 Aldemir, M. N. *et al.* Prognostic Value of Baseline Neutrophil-Lymphocyte and Platelet-Lymphocyte Ratios in Local and Advanced Gastric Cancer Patients. *Asian Pacific journal of cancer prevention : APJCP* **16**, 5933-5937 (2015).

191 Kadota, K. *et al.* Prognostic Impact of Immune Microenvironment in Lung Squamous Cell Carcinoma: Tumor-Infiltrating CD10+ Neutrophil/CD20+ Lymphocyte Ratio as an Independent Prognostic Factor. *Journal of thoracic oncology : official publication of the International Association for the Study of Lung Cancer* **10**, 1301-1310, doi:10.1097/jto.0000000000000617 (2015).

192 Neofytou, K. *et al.* The Preoperative Lymphocyte-to-Monocyte Ratio is Prognostic of Clinical Outcomes for Patients with Liver-Only Colorectal Metastases in the Neoadjuvant Setting. *Annals of surgical oncology* **22**, 4353-4362, doi:10.1245/s10434-015-4481-8 (2015).

193 Bagante, F. *et al.* Neutrophil-lymphocyte and platelet-lymphocyte ratio as predictors of disease specific survival after resection of adrenocortical carcinoma. *Journal of surgical oncology* **112**, 164-172, doi:10.1002/jso.23982 (2015).

194 Wang, Q. *et al.* The Severity of Liver Fibrosis Influences the Prognostic Value of Inflammation-Based Scores in Hepatitis B-Associated Hepatocellular Carcinoma. *Annals of surgical oncology* **22 Suppl 3**, S1125-1132, doi:10.1245/s10434-015-4598-9 (2015).
195 Pine, J. K. *et al.* Systemic neutrophil-to-lymphocyte ratio in colorectal cancer: the relationship to patient survival, tumour biology and local lymphocytic response to tumour. *British journal of cancer* **113**, 204-211, doi:10.1038/bjc.2015.87 (2015).
196 Li, J., Lin, J., Luo, Y., Kuang, M. & Liu, Y. Multivariate Analysis of Prognostic Biomarkers in Surgically Treated Endometrial Cancer. *PloS one* **10**, e0130640, doi:10.1371/journal.pone.0130640 (2015).
197 Zhang, H. *et al.* Clinical significance of preoperative neutrophil-lymphocyte vs platelet-lymphocyte ratio in primary operable patients with non-small cell lung cancer. *American journal of surgery* **210**, 526-535, doi:10.1016/j.amjsurg.2015.03.022 (2015).
198 Zhang, Y., Jiang, C., Li, J., Sun, J. & Qu, X. Prognostic significance of preoperative neutrophil/lymphocyte ratio and platelet/lymphocyte ratio in patients with gallbladder carcinoma. *Clinical & translational oncology : official publication of the Federation of Spanish Oncology Societies and of the National Cancer Institute of Mexico* **17**, 810-818, doi:10.1007/s12094-015-1310-2 (2015).
199 Qu, J. L. *et al.* Prognostic Model Based on Systemic Inflammatory Response and Clinicopathological Factors to Predict Outcome of Patients with Node-Negative Gastric Cancer. *PloS one* **10**, e0128540, doi:10.1371/journal.pone.0128540 (2015).
200 Zhang, W. W., Liu, K. J., Hu, G. L. & Liang, W. J. Preoperative platelet/lymphocyte ratio is a superior prognostic factor compared to other systemic inflammatory response markers in ovarian cancer patients. *Tumour biology : the journal of the International Society for Oncodevelopmental Biology and Medicine* **36**, 8831-8837, doi:10.1007/s13277-015-3533-9 (2015).
201 Yu, L., Lv, C. Y., Yuan, A. H., Chen, W. & Wu, A. W. Significance of the preoperative neutrophil-to-lymphocyte ratio in the prognosis of patients with gastric cancer. *World journal of gastroenterology* **21**, 6280-6286, doi:10.3748/wjg.v21.i20.6280 (2015).
202 Duan, H. *et al.* Prognostic role of neutrophil-lymphocyte ratio in operable esophageal squamous cell carcinoma. *World journal of gastroenterology* **21**, 5591-5597, doi:10.3748/wjg.v21.i18.5591 (2015).
203 Wen, R. M. *et al.* Preoperative Neutrophil to Lymphocyte Ratio as a Prognostic Factor in Patients with Non-metastatic Renal Cell Carcinoma. *Asian Pacific journal of cancer prevention : APJCP* **16**, 3703-3708 (2015).
204 Choi, W. J. *et al.* Preoperative Neutrophil-to-Lymphocyte Ratio is a Better Prognostic Serum Biomarker than Platelet-to-Lymphocyte Ratio in Patients Undergoing Resection for Nonmetastatic Colorectal Cancer. *Annals of surgical oncology* **22 Suppl 3**, S603-613, doi:10.1245/s10434-015-4571-7 (2015).
205 Deng, Q. *et al.* Prognostic value of pre-operative inflammatory response biomarkers in gastric cancer patients and the construction of a predictive model. *Journal of translational medicine* **13**, 66, doi:10.1186/s12967-015-0409-0 (2015).
206 Spolverato, G. *et al.* Neutrophil-lymphocyte and platelet-lymphocyte ratio in patients after resection for hepato-pancreatico-biliary malignancies. *Journal of surgical oncology* **111**, 868-874, doi:10.1002/jso.23900 (2015).
207 Han, L. H. *et al.* Prognostic significance of preoperative lymphocyte-monocyte ratio in patients with resectable esophageal squamous cell carcinoma. *Asian Pacific journal of cancer prevention : APJCP* **16**, 2245-2250 (2015).

208 Kim, E. Y., Lee, J. W., Yoo, H. M., Park, C. H. & Song, K. Y. The Platelet-to-Lymphocyte Ratio Versus Neutrophil-to-Lymphocyte Ratio: Which is Better as a Prognostic Factor in Gastric Cancer? *Annals of surgical oncology* **22**, 4363-4370, doi:10.1245/s10434-015-4518-z (2015).

209 Chan, A. W. *et al.* Prognostic Nutritional Index (PNI) Predicts Tumor Recurrence of Very Early/Early Stage Hepatocellular Carcinoma After Surgical Resection. *Annals of surgical oncology* **22**, 4138-4148, doi:10.1245/s10434-015-4516-1 (2015).

210 Choi, J. E. *et al.* Perioperative neutrophil:lymphocyte ratio and postoperative NSAID use as predictors of survival after lung cancer surgery: a retrospective study. *Cancer medicine* **4**, 825-833, doi:10.1002/cam4.428 (2015).

211 Lee, S. K. *et al.* Immediate postoperative inflammation is an important prognostic factor in breast cancer. *Oncology* **88**, 337-344, doi:10.1159/000368985 (2015).

212 Chen, Z. Y. *et al.* Cytokine profile and prognostic significance of high neutrophil-lymphocyte ratio in colorectal cancer. *British journal of cancer* **112**, 1088-1097, doi:10.1038/bjc.2015.61 (2015).

213 Wuxiao, Z. J. *et al.* A prognostic model to predict survival in stage III colon cancer patients based on histological grade, preoperative carcinoembryonic antigen level and the neutrophil lymphocyte ratio. *Asian Pacific journal of cancer prevention : APJCP* **16**, 747-751 (2015).

214 Chen, Q. *et al.* The elevated preoperative neutrophil-to-lymphocyte ratio predicts poor prognosis in intrahepatic cholangiocarcinoma patients undergoing hepatectomy. *Tumour biology : the journal of the International Society for Oncodevelopmental Biology and Medicine* **36**, 5283-5289, doi:10.1007/s13277-015-3188-6 (2015).

215 Kim, M. *et al.* Prognostic value of systemic inflammatory responses in patients with upper urinary tract urothelial carcinoma. *World journal of urology* **33**, 1439-1457, doi:10.1007/s00345-015-1484-9 (2015).

216 Szkandera, J. *et al.* The derived neutrophil/lymphocyte ratio predicts poor clinical outcome in soft tissue sarcoma patients. *American journal of surgery* **210**, 111-116, doi:10.1016/j.amjsurg.2014.10.021 (2015).

217 Ben, Q. *et al.* Validation of the pretreatment neutrophil-lymphocyte ratio as a predictor of overall survival in a cohort of patients with pancreatic ductal adenocarcinoma. *Pancreas* **44**, 471-477, doi:10.1097/mpa.0000000000000271 (2015).

218 Graziosi, L. *et al.* Prognostic value of preoperative neutrophils to lymphocytes ratio in patients resected for gastric cancer. *American journal of surgery* **209**, 333-337, doi:10.1016/j.amjsurg.2014.06.014 (2015).

219 Takahashi, R. *et al.* Prognostic significance of systemic neutrophil and leukocyte alterations in surgically treated endometrial cancer patients: a monoinstitutional study. *Gynecologic oncology* **137**, 112-118, doi:10.1016/j.ygyno.2015.02.006 (2015).

220 Shirai, Y. *et al.* Preoperative platelet to lymphocyte ratio predicts outcome of patients with pancreatic ductal adenocarcinoma after pancreatic resection. *Surgery* **158**, 360-365, doi:10.1016/j.surg.2015.03.043 (2015).

221 Chen, Q. *et al.* Negative impact of preoperative platelet-lymphocyte ratio on outcome after hepatic resection for intrahepatic cholangiocarcinoma. *Medicine* **94**, e574, doi:10.1097/md.0000000000000574 (2015).

222 Lian, L. *et al.* Application of platelet/lymphocyte and neutrophil/lymphocyte ratios in early diagnosis and prognostic prediction in patients with resectable gastric cancer. *Cancer biomarkers : section A of Disease markers* **15**, 899-907, doi:10.3233/cbm-150534 (2015).

223 Xie, X., Luo, K. J., Hu, Y., Wang, J. Y. & Chen, J. Prognostic value of preoperative platelet-lymphocyte and neutrophil-lymphocyte ratio in patients undergoing surgery for esophageal squamous cell cancer. *Dis Esophagus* **29**, 79-85, doi:10.1111/dote.12296 (2016).

224 Mohri, Y. *et al.* Impact of Preoperative Neutrophil to Lymphocyte Ratio and Postoperative Infectious Complications on Survival After Curative Gastrectomy for Gastric Cancer: A Single Institutional Cohort Study. *Medicine* **95**, e3125, doi:10.1097/md.0000000000003125 (2016).

225 Takahashi, Y. *et al.* Neutrophil-Lymphocyte Ratio as a Prognostic Marker for Lung Adenocarcinoma After Complete Resection. *World journal of surgery* **40**, 365-372, doi:10.1007/s00268-015-3275-2 (2016).

226 Cheng, Y. C. *et al.* The Prognostic Significance of Inflammation-Associated Blood Cell Markers in Patients with Upper Tract Urothelial Carcinoma. *Annals of surgical oncology* **23**, 343-351, doi:10.1245/s10434-015-4781-z (2016).

227 Turner, N. *et al.* Analysis of local chronic inflammatory cell infiltrate combined with systemic inflammation improves prognostication in stage II colon cancer independent of standard clinicopathologic criteria. *International journal of cancer* **138**, 671-678, doi:10.1002/ijc.29805 (2016).

228 Fu, Y., Liu, W., OuYang, D., Yang, A. & Zhang, Q. Preoperative Neutrophil-to-lymphocyte Ratio Predicts Long-term Survival in Patients Undergoing Total Laryngectomy With Advanced Laryngeal Squamous Cell Carcinoma: A Single-center Retrospective Study. *Medicine* **95**, e2689, doi:10.1097/md.0000000000002689 (2016).

229 Lu, S. D. *et al.* Preoperative Ratio of Neutrophils to Lymphocytes Predicts Postresection Survival in Selected Patients With Early or Intermediate Stage Hepatocellular Carcinoma. *Medicine* **95**, e2722, doi:10.1097/md.0000000000002722 (2016).

230 Chen, P. C. & Feng, J. F. A Novel Inflammation-Based Stage (I Stage) in Patients with Resectable Esophageal Squamous Cell Carcinoma. *Mediators of inflammation* **2016**, 5396747, doi:10.1155/2016/5396747 (2016).

231 Wang, S. C. *et al.* Pretreatment Neutrophil to Lymphocyte Ratio Independently Predicts Disease-specific Survival in Resectable Gastroesophageal Junction and Gastric Adenocarcinoma. *Annals of surgery* **263**, 292-297, doi:10.1097/sla.0000000000001189 (2016).

232 Hodek, M. *et al.* Neoadjuvant chemoradiotherapy of rectal carcinoma : Baseline hematologic parameters influencing outcomes. *Strahlentherapie und Onkologie : Organ der Deutschen Rontgengesellschaft ... [et al]* **192**, 632-640, doi:10.1007/s00066-016-0988-6 (2016).

233 Morizawa, Y. *et al.* Neutrophil-to-lymphocyte ratio as a detection marker of tumor recurrence in patients with muscle-invasive bladder cancer after radical cystectomy. *Urologic oncology* **34**, 257.e211-257, doi:10.1016/j.urolonc.2016.02.012 (2016).

234 Kosumi, K. *et al.* Neutrophil/lymphocyte ratio predicts the prognosis in esophageal squamous cell carcinoma patients. *Surgery today* **46**, 405-413, doi:10.1007/s00595-015-1197-0 (2016).

235 Kawahara, T. *et al.* Neutrophil-to-lymphocyte ratio is a prognostic marker in bladder cancer patients after radical cystectomy. *BMC cancer* **16**, 185, doi:10.1186/s12885-016-2219-z (2016).

236 Kang, M., Jeong, C. W., Kwak, C., Kim, H. H. & Ku, J. H. The Prognostic Significance of the Early Postoperative Neutrophil-to-Lymphocyte Ratio in Patients with Urothelial Carcinoma of the Bladder Undergoing Radical Cystectomy. *Annals of surgical oncology* **23**, 335-342, doi:10.1245/s10434-015-4708-8 (2016).

237 Bhindi, B. *et al.* Identification of the best complete blood count-based predictors for bladder cancer outcomes in patients undergoing radical cystectomy. *British journal of cancer* **114**, 207-212, doi:10.1038/bjc.2015.432 (2016).

238 Raungkaewmanee, S., Tangjitgamol, S., Manusirivithaya, S., Srijaipracharoen, S. & Thavaramara, T. Platelet to lymphocyte ratio as a prognostic factor for epithelial ovarian cancer. *Journal of gynecologic oncology* **23**, 265-273, doi:10.3802/jgo.2012.23.4.265 (2012).

239 Feng, J. F., Huang, Y., Zhao, Q. & Chen, Q. X. Clinical significance of preoperative neutrophil lymphocyte ratio versus platelet lymphocyte ratio in patients with small cell carcinoma of the esophagus. *ScientificWorldJournal* **2013**, 504365, doi:10.1155/2013/504365 (2013).

240 Baranyai, Z. *et al.* The comparison of thrombocytosis and platelet-lymphocyte ratio as potential prognostic markers in colorectal cancer. *Thrombosis and haemostasis* **111**, 483-490, doi:10.1160/th13-08-0632 (2014).

241 Krenn-Pilko, S. *et al.* The elevated preoperative platelet-to-lymphocyte ratio predicts poor prognosis in breast cancer patients. *British journal of cancer* **110**, 2524-2530, doi:10.1038/bjc.2014.163 (2014).

242 Szkandera, J. *et al.* The elevated preoperative platelet to lymphocyte ratio predicts decreased time to recurrence in colon cancer patients. *American journal of surgery* **208**, 210-214, doi:10.1016/j.amjsurg.2013.10.030 (2014).

243 Zhang, G. M. *et al.* Preoperative lymphocyte-monocyte and platelet-lymphocyte ratios as predictors of overall survival in patients with bladder cancer undergoing radical cystectomy. *Tumour biology : the journal of the International Society for Oncodevelopmental Biology and Medicine* **36**, 8537-8543, doi:10.1007/s13277-015-3613-x (2015).

244 Messager, M., Neofytou, K., Chaudry, M. A. & Allum, W. H. Prognostic impact of preoperative platelets to lymphocytes ratio (PLR) on survival for oesophageal and junctional carcinoma treated with neoadjuvant chemotherapy: A retrospective monocentric study on 153 patients. *European journal of surgical oncology : the journal of the European Society of Surgical Oncology and the British Association of Surgical Oncology* **41**, 1316-1323, doi:10.1016/j.ejso.2015.06.007 (2015).

245 Pang, Q. *et al.* Platelet to lymphocyte ratio as a novel prognostic tool for gallbladder carcinoma. *World journal of gastroenterology* **21**, 6675-6683, doi:10.3748/wjg.v21.i21.6675 (2015).

246 Ozawa, T. *et al.* The preoperative platelet to lymphocyte ratio is a prognostic marker in patients with stage II colorectal cancer. *International journal of colorectal disease* **30**, 1165-1171, doi:10.1007/s00384-015-2276-9 (2015).

247 Saito, H. *et al.* A new prognostic scoring system using factors available preoperatively to predict survival after operative resection of perihilar cholangiocarcinoma. *Surgery* **159**, 842-851, doi:10.1016/j.surg.2015.10.027 (2016).

248 Stotz, M. *et al.* The preoperative lymphocyte to monocyte ratio predicts clinical outcome in patients with stage III colon cancer. *British journal of cancer* **110**, 435-440, doi:10.1038/bjc.2013.785 (2014).

249 Hu, P. *et al.* Prognostic significance of systemic inflammation-based lymphocyte- monocyte ratio in patients with lung cancer: based on a large cohort study. *PloS one* **9**, e108062, doi:10.1371/journal.pone.0108062 (2014).

250 Zhou, X. *et al.* The preoperative lymphocyte to monocyte ratio predicts clinical outcomes in patients with stage II/III gastric cancer. *Tumour biology : the journal of the International Society for Oncodevelopmental Biology and Medicine* **35**, 11659-11666, doi:10.1007/s13277-014-2504-x (2014).

251 Hutterer, G. C. *et al.* Low preoperative lymphocyte-monocyte ratio (LMR) represents a potentially poor prognostic factor in nonmetastatic clear cell renal cell carcinoma. *Urologic oncology* **32**, 1041-1048, doi:10.1016/j.urolonc.2014.04.001 (2014).

252 Wen, J. *et al.* Prognostic Significance of Preoperative Circulating Monocyte Count in Patients With Breast Cancer: Based on a Large Cohort Study. *Medicine* **94**, e2266, doi:10.1097/md.0000000000002266 (2015).
253 Lin, Z. X. *et al.* Lymphocyte-to-monocyte ratio predicts survival of patients with hepatocellular carcinoma after curative resection. *World journal of gastroenterology* **21**, 10898-10906, doi:10.3748/wjg.v21.i38.10898 (2015).
254 Yoshida, T. *et al.* A novel risk stratification model, involving preoperative lymphocyte-monocyte ratio and standard pathological factors, for overall survival in patients with bladder cancer undergoing radical cystectomy. *Japanese journal of clinical oncology* **45**, 1162-1167, doi:10.1093/jjco/hyv146 (2015).
255 Yamagishi, T. *et al.* Prognostic significance of the lymphocyte-to-monocyte ratio in patients with malignant pleural mesothelioma. *Lung cancer (Amsterdam, Netherlands)* **90**, 111-117, doi:10.1016/j.lungcan.2015.07.014 (2015).
256 Ozawa, T. *et al.* Impact of a lymphocyte to monocyte ratio in stage IV colorectal cancer. *The Journal of surgical research* **199**, 386-392, doi:10.1016/j.jss.2015.06.014 (2015).
257 Hutterer, G. C. *et al.* Pretreatment lymphocyte-monocyte ratio as a potential prognostic factor in a cohort of patients with upper tract urothelial carcinoma. *Journal of clinical pathology* **68**, 351-355, doi:10.1136/jclinpath-2014-202658 (2015).
258 Huang, Y. & Feng, J. F. Low preoperative lymphocyte to monocyte ratio predicts poor cancer-specific survival in patients with esophageal squamous cell carcinoma. *OncoTargets and therapy* **8**, 137-145, doi:10.2147/ott.s73794 (2015).
259 Chen, L., Zhang, F., Sheng, X. G. & Zhang, S. Q. Decreased pretreatment lymphocyte/monocyte ratio is associated with poor prognosis in stage Ib1-IIa cervical cancer patients who undergo radical surgery. *OncoTargets and therapy* **8**, 1355-1362, doi:10.2147/ott.s82174 (2015).
260 Peng, W. *et al.* Prognostic value of the platelet to lymphocyte ratio change in liver cancer. *The Journal of surgical research* **194**, 464-470, doi:10.1016/j.jss.2014.12.021 (2015).
261 Peng, W. *et al.* Neutrophil to lymphocyte ratio changes predict small hepatocellular carcinoma survival. *The Journal of surgical research* **192**, 402-408, doi:10.1016/j.jss.2014.05.078 (2014).

Search Details, Dates and Personnel:

- Keywords per database
    - US National Library of Medicine (MEDLINE): Cancer, GPS, Glasgow Prognostic Score, mGPS, modified Glasgow Prognostic Score, NLR, Neutrophil Lymphocyte Ratio, LMR, Leucocyte Monocyte Ratio, PLR, Platelet Lymphocyte Ratio
    - Excerpta Medica database (EMBASE): Cancer, GPS, Glasgow Prognostic Score, mGPS, modified Glasgow Prognostic Score, NLR, Neutrophil Lymphocyte Ratio, LMR, Leucocyte Monocyte Ratio, PLR, Platelet Lymphocyte Ratio
    - Cochrane Database of Systematic Reviews (CDSR): Cancer, GPS, Glasgow Prognostic Score, mGPS, modified Glasgow Prognostic Score, NLR, Neutrophil Lymphocyte Ratio, LMR, Leucocyte Monocyte Ratio, PLR, Platelet Lymphocyte Ratio
- Search Dates: Until December 2016
- Search Personnel: Mr Ross Dolan and Mr Jason Lim

PRISMA Checklist:

Section/topic	#	Checklist item	Reported on page #
**TITLE**			
Title	1	Identify the report as a systematic review, meta-analysis, or both.	1
**ABSTRACT**			
Structured summary	2	Provide a structured summary including, as applicable: background; objectives; data sources; study eligibility criteria, participants, and interventions; study appraisal and synthesis methods; results; limitations; conclusions and implications of key findings; systematic review registration number.	2
**INTRODUCTION**			
Rationale	3	Describe the rationale for the review in the context of what is already known.	3-4
Objectives	4	Provide an explicit statement of questions being addressed with reference to participants, interventions, comparisons, outcomes, and study design (PICOS).	4
**METHODS**			
Protocol and registration	5	Indicate if a review protocol exists, if and where it can be accessed (e.g., Web address), and, if available, provide registration information including registration number.	NA
Eligibility criteria	6	Specify study characteristics (e.g., PICOS, length of follow-up) and report characteristics (e.g., years considered, language, publication status) used as criteria for eligibility, giving rationale.	4-5
Information sources	7	Describe all information sources (e.g., databases with dates of coverage, contact with study authors to identify additional studies) in the search and date last searched.	4-5
Search	8	Present full electronic search strategy for at least one database, including any limits used, such that it could be repeated.	4-5
Study selection	9	State the process for selecting studies (i.e., screening, eligibility, included in systematic review, and, if applicable, included in the meta-analysis).	4-5
Data collection process	10	Describe method of data extraction from reports (e.g., piloted forms, independently, in duplicate) and any processes for obtaining and confirming data from investigators.	4-5
Data items	11	List and define all variables for which data were sought (e.g., PICOS, funding sources) and any assumptions and simplifications made.	4-5

Risk of bias in individual studies	12	Describe methods used for assessing risk of bias of individual studies (including specification of whether this was done at the study or outcome level), and how this information is to be used in any data synthesis.	4-5
Summary measures	13	State the principal summary measures (e.g., risk ratio, difference in means).	4-5
Synthesis of results	14	Describe the methods of handling data and combining results of studies, if done, including measures of consistency (e.g., I^2) for each meta-analysis.	4-5
**Section/topic**	**#**	**Checklist item**	**Reported on page #**
Risk of bias across studies	15	Specify any assessment of risk of bias that may affect the cumulative evidence (e.g., publication bias, selective reporting within studies).	4-5
Additional analyses	16	Describe methods of additional analyses (e.g., sensitivity or subgroup analyses, meta-regression), if done, indicating which were pre-specified.	4-5
**RESULTS**			
Study selection	17	Give numbers of studies screened, assessed for eligibility, and included in the review, with reasons for exclusions at each stage, ideally with a flow diagram.	5-4
Study characteristics	18	For each study, present characteristics for which data were extracted (e.g., study size, PICOS, follow-up period) and provide the citations.	5-4
Risk of bias within studies	19	Present data on risk of bias of each study and, if available, any outcome level assessment (see item 12).	19<br>Fig 2-28
Results of individual studies	20	For all outcomes considered (benefits or harms), present, for each study: (a) simple summary data for each intervention group (b) effect estimates and confidence intervals, ideally with a forest plot.	5-19<br>(Fig 2-28)
Synthesis of results	21	Present results of each meta-analysis done, including confidence intervals and measures of consistency.	5-19<br>(Fig 2-28)
Risk of bias across studies	22	Present results of any assessment of risk of bias across studies (see Item 15).	19<br>Fig 2-28
Additional analysis	23	Give results of additional analyses, if done (e.g., sensitivity or subgroup analyses, meta-regression [see Item 16]).	5-19<br>(Fig 2-28)
**DISCUSSION**			
Summary of evidence	24	Summarize the main findings including the strength of evidence for each main outcome; consider their relevance to key groups (e.g., healthcare providers, users, and policy makers).	20-23

Limitations	25	Discuss limitations at study and outcome level (e.g., risk of bias), and at review-level (e.g., incomplete retrieval of identified research, reporting bias).	22
Conclusions	26	Provide a general interpretation of the results in the context of other evidence, and implications for future research.	23
**FUNDING**			
Funding	27	Describe sources of funding for the systematic review and other support (e.g., supply of data); role of funders for the systematic review.	N/A

From: Moher D, Liberati A, Tetzlaff J, Altman DG, The PRISMA Group (2009). Preferred Reporting Items for Systematic Reviews and Meta-Analyses: The PRISMA Statement. PLoS Med 6(6): e1000097. doi:10
